# Revision of the Neotropical hoverfly genus *Peradon* Reemer (Diptera, Syrphidae, Microdontinae)

**DOI:** 10.3897/zookeys.896.36493

**Published:** 2019-12-05

**Authors:** Menno Reemer, Jeffrey H. Skevington, Scott Kelso

**Affiliations:** 1 Naturalis Biodiversity Center, P.O. Box 9517, 2300 RA Leiden, the Netherlands Naturalis Biodiversity Center Leiden Netherlands; 2 Canadian National Collection of Insects, Arachnids and Nematodes, Agriculture and Agri-Food Canada, 960 Carling Avenue, K.W. Neatby Building, Ottawa, ON, K1A 0C6, Canada Agriculture and Agri-Food Canada Ottawa Canada

**Keywords:** COI barcodes, identification key, morphology, new species, new synonyms

## Abstract

The species of the Neotropical hoverfly genus *Peradon* Reemer, 2013 are revised, based on morphological characters with aid of mitochondrial DNA barcodes. The resulting number of valid species is increased to 31, of which the following seven are described as new: *P.ballux* Reemer, **sp. nov.**, *P.brevis* Reemer, **sp. nov.**, *P.costaricensis* Reemer, **sp. nov.**, *P.notialus* Reemer, **sp. nov.**, *P.palpator* Reemer, **sp. nov.**, *P.pompiloides* Reemer, **sp. nov.**, and *P.surinamensis* Reemer, **sp. nov.** Two new synonymies are established: *Microdonlangi* Curran, 1925, **syn. nov.** and *Microdonflavomarginatum* Curran, 1925, **syn. nov.** are both junior synonyms of *Muliobidens* Fabricius, 1805. A neotype is designated for *Microdondiaphanus* Sack, 1921. This neotype, which has been reared from an ant nest, also represents the first case of a larval record for this genus. In some species, most notably in *P.bidens* (Fabricius) and *P.normalis* (Curran), discrete and distinct colour morphs are recognized, with strongly differing colouration of wings and abdomen.

## Introduction

The genus *Peradon* Reemer, 2013 (type species: *Muliobidens* Fabricius, 1805) was erected to accommodate several Neotropical hoverfly species formerly included in *Microdon* Meigen, 1803. The morphological diversity of the species makes for a colourful genus (Figs 1, 2, 97–148). Reemer and Ståhls (2013a) distinguished three morphological species groups in this genus, i.e., *bidens* group, *flavofascium* group, and *trivittatus* group. Representatives of these species groups were recovered together with high support values in a phylogenetic clade based on an analysis of combined molecular and morphological characters (Reemer and Ståhls 2013b). Based on these findings, *Peradon* is considered to be a monophyletic group restricted to the Neotropics.

Very little is known about the biology of *Peradon* species. Until now, no larval records are known (Reemer 2013a), but the present paper provides the first record of larvae of *P.diaphanus* (Sack, 1921) from a colony of an unidentified ant. Observations on adult *P.bidens* (Fabricius, 1805) and *P.trivittatus* (Curran, 1925) in Suriname as noted by Reemer (2014) suggest that males behave in a fashion arguably interpretable as territorial, flying off and on, and resting at the same patch of ground intermittently.

Although a few *Peradon* species are included in identification keys to Neotropical Microdontinae by Curran (1934, 1940, 1941), there is no key to the 24 species listed by Reemer and Ståhls (2013a). Additionally, two species were described recently (Reemer 2014) and several additional species are awaiting description. Thus, a species revision is our main objective in order to make possible reliable species identification. Moreover, Reemer (2014) noted that specimens of *Peradonlangi* (Curran, 1925) are morphologically identical to specimens of *P.bidens* (Fabricius), and only differ from those in the colouration of the abdomen: black in *P.langi*, red in *P.bidens*. Reemer (2014) considered the possibility of these taxa being colour morphs of the same species, but this has not yet been resolved. A similar case of possible colour morphs between *Peradon* SUR-17a and *Peradon* SUR-17b was presented by Reemer (2014). Both cases are addressed in the present paper with aid of mitochondrial DNA barcodes.

**Figures 1, 2.**
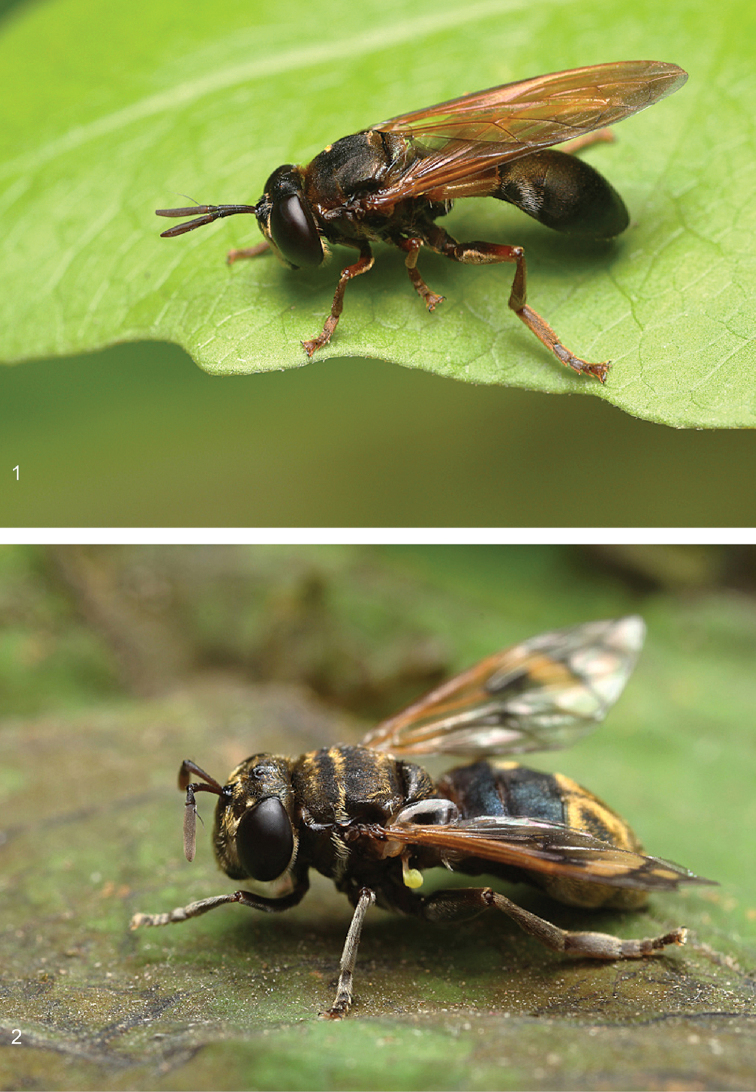
Field images of *Peradon* species. Both photos were taken in Peru, San Martin, around San Ruque de Cumbaza, 15-31.I.2015, by Tim Faasen **1***P.oligonax* male **2***P.aurigaster* female.

## Materials and methods

### Examined collections

Specimens from the following institutional and private collections have been examined:

**AMNH**American Museum of Natural History, New York (USA);

**CAS**California Academy of Sciences, San Francisco (USA);

**CNC**Canadian National Collection of Insects, Arachnids and Nematodes, Ottawa (Canada);

**CSCA**California State Collection of Arthropods, Sacramento (USA);

**CU**Cornell University, Ithaca (USA);

**DEBU**University of Guelph Insect Collection, Guelph (Canada);

**DEI**Senckenberg Deutsches Entomologisches Institut, Müncheberg (Germany);

**INBIO**Instituto Nacional de Biodiversidad, Heredia (Costa Rica);

**INPA**National Institute of Amazonian Research, Manaus (Brazil);

**JTS** J.T. Smit, Utrecht (Netherlands);

**LACM**Natural History Museum of Los Angeles County, Los Angeles (USA);

**MCZ**Museum of Comparative Zoology, Harvard (USA);

**MRSN**Museu Regionale di Scienze Naturali, Turin (Italy);

**MNHN**Muséum national d’Histoire naturelle, Paris (France);

**MZH**Finnish Museum of Natural History, Helsinki (Finland);

**NHMUK**British Museum of Natural History, London (United Kingdom);

**RMNH**Naturalis Biodiversity Center, Leiden (Netherlands);

**SEMC**Snow Entomological Collections, University of Kansas (USA);

**SMF**Forschungsinstitut und Naturmuseum Senckenberg, Frankfurt-am-Main (Germany);

**SNSD** Senckenberg Naturhistorische Sammlungen, Dresden (Germany);

**UFLA**Universidade Federal de Lavras (Brazil);

**UFPR**Universidade Federal do Paraná, Curitiba (Brazil);

**USNM**United States National Museum, Smithsonian Institution, Washington DC (USA);

**UZMC**Zoological Museum University of Copenhagen, Copenhagen (Denmark);

**ZFMK**Zoologisches Forschungsmuseum Alexander Koenig, Bonn (Germany).

### Morphology

Dissected male genitalia were macerated in cold lactic acid (70–75%) for 24 hours and were subsequently stored in glycerol. Drawings were made using a drawing tube attached to a Wild M20 microscope. Morphological terminology follows Cumming and Wood (2017), and its application to Microdontinae derives from Reemer and Ståhls (2013b). In the measurements of ratios of head width to vertex width in dorsal view, head width is measured at the widest point, whereas vertex width is measured at the narrowest point (Fig. 19). Length ratios of the antennal segments are given as scape:pedicel:basoflagellomere, but sometimes the pedicel is omitted as this is only very short compared to the other two segments. Some of the figures in the wing plates have been mirrored for the sake of comparability.

### DNA extraction and sequencing

Table 1 lists all specimens used in the present study, depository institution, and database details together with BOLD process IDs and GenBank accession numbers. For some *Peradon* species DNA barcodes were published in BOLD and GenBank from previous projects. For the *Peradon* specimens newly sequenced for this study (Table 1, BOLD Ids starting with CNC or PERAD), DNA extraction was attempted using the front or mid legs of a selection of *Peradon* specimens collected relatively recently (2004–2015). These specimens were processed using the methodology here detailed.

**Table 1.** *Peradon* and outgroup specimens used for barcode analysis. Unique identifiers: MR codes are used in Figure 8 and refer to the personal database of the author; CNC codes can be found in the online database of the CNC (https://cnc.agr.gc.ca); INBIO codes are used by INBIO; Y codes are used in a MZH lab database managed by Gunilla Ståhls. BOLD, GenBank and ENA (European Nucleotide Archive) are online databases for DNA and RNA sequences, in which the barcodes used in the present analysis are stored.

**Taxon**	**Unique identifier / Lab code**	**Locality and year of collection**	**Collector**	**Deposition**	**BOLD process ID**	**GenBank / ENA accession**
**Outgroup**						
*Paragodonparagoides* ♀	MR0341 / Y1314	Costa Rica, Alajuela, 2010	Porras, W. and A. Rojas	RMNH	–	LR699066
*Menidonfalcatus* ♂	MR0351 / Y1324	Costa Rica, Heredia	Reemer, M.	RMNH	–	LR699065
*Microdonmajor* ♂	MR0350 / Y1323	Netherlands, Kootwijk	Reemer, M.	RMNH	–	LR699064
**Ingroup**						
*Peradonangustiventris* ♂	MR1033 / CNC870143	Suriname, Colakreek, 2006	Reemer, M.	RMNH	PERAD017-17	–
*Peradonaurigaster* ♀	MR0563 / CNC464836	Peru, San Martin, 2015	Faasen, T.	RMNH	CNCFF788-16	–
*Peradonbidens* red morph ♂	MR1289 / Y0578	Suriname, Peperpot, 2006	Reemer, M.	MZH	–	HF547911.1
*Peradonbidens* red morph ♂	MR0037 / CNC870147	Suriname, Zanderij, 2006	Reemer, M.	RMNH	PERAD021-17	–
*Peradonbidens* black morph ♂	MR0038 / CNC870145	Suriname, Colakreek, 2006	Reemer, M.	RMNH	PERAD019-17	–
*Peradonbidens* black morph ♂	MR1036 / CNC870146	Suriname, Mopentibo, 2006	Reemer, M.	RMNH	PERAD020-17	–
*Peradonchrysopygus* ♀	MR1285 / INB0003861150	Costa Rica, Heredia, 2004	Porras Vega, W.	INBIO	ASIND073-12	–
*Peradonchrysopygus* ♂	MR1286 / INB0003478737	Costa Rica, Guanacaste, 2002	Cardenas, Y.	INBIO	ASIND081-12	–
*Peradonchrysopygus* ♀	MR1287 / INB0004273155	Costa Rica, Alajuela, 2010	Azofeifa Zuniga, J.A.	INBIO	ASIND2431-12	–
*Peradonchrysopygus* ♀	MR1288 / INB0004304009	Costa Rica, Puntarenas, 2011	Haber, W.	INBIO	ASIND2586-12	–
*Peradonchrysopygus* ♂	MR0344 / Y1317	Costa Rica, Heredia	Reemer, M.	RMNH	–	HF585642
*Peradoncostaricensis* ♀	MR1028 / CNC870131	Costa Rica, Heredia, 2004	Stuke, J.-H.	ZFMK	PERAD005-17	–
*Peradonluridescens* ♂	MR1030 / CNC870133	Peru, Madre de Dios, 2011	Smit, J.T.	Smit, J.T.	PERAD007-17	–
*Peradonluridescens* ♂	MR1007 / CNC870138	Brazil, Acre, 2008	Melo, G.	UFPR	PERAD012-17	–
*Peradonluridescens* ♂	MR1034 / CNC870144	Suriname, Nassau Mts., 2006	Reemer, M.	RMNH	PERAD018-17	–
*Peradonnormalis* typical morph ♀	MR1032 / CNC870140	Peru, San Martin, 2005	Irwin, M.E. and J.D. Vasquez	CSCA	PERAD014-17	–
*Peradonnormalis* typical morph ♀	MR1158 / CNC870136	Suriname, Brownsberg, 2006	Reemer, M.	RMNH	PERAD10-17	–
*Peradonnormalis* SUR-17b morph♀	MR1001 / CNC870135	Suriname, Brownsberg, 2006	Reemer, M.	RMNH	PERAD09-17	–
*Peradonsciarus* ♂	MR0566 / CNC464839	Peru, San Martin, 2015	Faasen, T.	RMNH	CNCFF791-16	–
*Peradonsurinamensis*	MR0385 / CNC870141	Suriname, Colakreek, 2006	Reemer, M.	RMNH	PERAD015-17	–
*Peradontrivittatus* ♂	MR0887 / CNC102829	Colombia, Amazonas	Ward, D. and A. Forsyth	CNC	CNCDB1893-11	–
*Peradontrivittatus*	MR0564 / CNC464837	Peru, San Martin, 2015	Faasen, T.	RMNH	CNCFF789-16	–

Total DNA was extracted from single specimen legs using the DNeasy Tissue kit (Qiagen Inc., Santa Clara, CA, USA) following the manufacturer’s protocol. A 658-nucleotide fragment of the 5’ end of the mitochondrial gene cytochrome c oxidase subunit I (COI) was amplified using the primer pair LCO1490 and COI-Dipt-2183R (Gibson et al. 2011). 25 μl PCR reactions were performed with 15.7 μl ddH2O, 2.5 μl 10X Ex- Taq PCR buffer (containing 20 mM MgCl2), 0.65 μl 25 mM MgCl2, 1 μl of each 10 μM primer, 2 μl 10 mM dNTPs, 0.15 μl ExTaq HS DNA polymerase (Takara Bio USA, Madison, WI, USA), and 2 μl total DNA. Amplification cycles were performed on an Eppendorf ep Gradient S Mastercycler (Eppendorf AG, Hamburg, Germany). Amplification products and negative controls were visualized on 1% agarose electrophoresis gels and purified for sequencing using Clone-Well 0.8% Egels (Invitrogen, Carlsbad, CA, USA). Sequencing reactions were carried out in a volume of 10 μl using an ABI BigDye Terminator v3.1 Cycle Sequencing kit (PE Applied Biosystems, Foster City, CA, USA) for forward and reverse primers. Sequencing reactions were purified using the ABI ethanol/EDTA/sodium acetate precipitation protocol and analyzed on an ABI 3500xl Genetic Analyzer (PE Applied Biosystems, Foster City, CA, USA). Sequencing of purified PCR products was performed at the Canadian National Collection of Insects, Arachnids, and Nematodes, Agriculture & Agri-Food Canada (Ottawa, ON, Canada). All sequence chromatograms were edited and contigs formed using Sequencher 5.4.6 (Gene Codes Corp., Ann Arbor, MI, USA). Resulting contigs were hand-aligned using Mesquite 3.6 (Maddison and Maddison 2018).

### Barcode analysis

Three outgroup microdontine taxa were chosen: *Menidonfalcatus* (Williston, 1887), *Microdonmajor* Andries, 1912, and *Paragodonparagoides* Thompson, 1969. The tree topology was rooted on *Paragodonparagoides*. A maximum likelihood (ML) tree (with 100 bootstrap replicates) for a single concatenated matrix was estimated using RAxML v7.2.6 (Stamatakis 2006), with the GTR + G substitution model under default parameters. A parsimony analysis was conducted with the DNA barcodes using TNT software, version 1.5 (Goloboff and Catalano 2016). Most parsimonious trees were found by using the ‘implicit enumeration’ option in this program, which provides an exhaustive search of all possible trees. Branch lengths were calculated by the same program. Bootstrap support values and GC frequency differences (Goloboff et al. 2003) were calculated using TNT with 1,000 replicates in implicit numeration (exhaustive search). In addition, a Neighbor-Joining analysis was performed using MEGA7 software (Kumar et al. 2016).

## Results

### Barcode results

Full barcodes with 658 bp in length were obtained from 22 specimens belonging to 16 *Peradon* taxa (including colour forms), including the already available sequences in different repositories. Maximum Likelihood analysis of these DNA sequences yielded the tree shown in Fig. 8. Parsimony analysis resulted in 10 equally parsimonious trees, the strict consensus of which is identical in topology to the ML tree (Suppl. material 1: Figure S1). In the results of the Neighbor Joining analysis (Suppl. material 2: Figure S2), the major clades were identical, but there were small differences in the placement of species within the *bidens* species group.

Even though the focus of this analysis was to explore the relationships of the obtained sequences at the species level, it is interesting to see that representatives of the three recognized species groups were recovered in separate lineages, with the *trivittatus* group as sister to the *bidens* + *flavofascium* groups. However, many species were not included in the present survey.

The *trivittatus* group is represented by only one species, so nothing can be said about intraspecific relationships within this group. The results for the *flavofascium* group show clear differences between the three included species. Within the *bidens* clade, a relatively long branch separates the Central American species *P.costaricensis* from the other included species, which are all from South America. The taxa within the remaining part of the *bidens* clade are separated by very short branches (supported by low bootstrap and GC values in the parsimony tree, Suppl. material 1: Fig. S1). Several of the branches within the *bidens* species group have very low support, a fact corroborated by the difference of only one or two nucleotides between sequences of different species.

Although three specimens of *P.luridescens* (Walker) and one of *P.angustiventris* (Macquart) were included in order to explore the taxonomic status of these two very similar taxa relative to each other, results did not provide any evidence to help on this matter.

Remarkably, two pairs of specimens each consisting of one *P.bidens* and one *P.langi* specimen (included in the ML tree as red and black morph of *P.bidens*, respectively) are recovered in separate parts of the *bidens* clade (this is also the case in the parsimony and NJ trees, Suppl. material 1: Fig. S1 and Suppl. material 2: Fig. S2). Within these pairs, the specimens have 100% identical barcodes. However, between the pairs there are only a few differences, which result in separate clades. As these specimens are morphologically identical, and have been collected at localities in close proximity, they are here considered to be conspecific, with *P.langi* considered as a black colour form of *P.bidens* (for more on this see account of *P.bidens* below). Apparently, at least two haplotypes occur which are not correlated to the colour morph (red or black abdomen).

There is another pair of specimens in the *bidens* species group with 100% identical barcodes: *Peradonnormalis* (Curran) typical morph (= *Peradon* SUR-17a of Reemer 2014) and *P.normalis* SUR-17b morph. These specimens differ strongly in their wing colouration but are otherwise morphologically identical. Moreover, they have been collected at the same locality at the same date. Based on the identical morphological characters and the identical molecular sequences, we conclude that the specimens are colour forms of the same species. See species account of *P.normalis* for further discussion.

### *Peradon* Reemer, 2013

*Peradon* species range in body length from 6 to 19.5 mm. Their body is more or less elongate, with an abdomen that is at most slightly wider than the thorax and is basally constricted in some species. Body colouration varies from entirely dull black to largely brown, red or yellowish. The wings are often partly yellow, brown or blackish. In some species, the pile on the thorax and/or abdomen is thick and golden to silvery. The colour patterns of several species seem to mimic those of certain Neotropical aculeates of the families Pompilidae and Vespidae (Hymenoptera).

**Figures 3–7.**
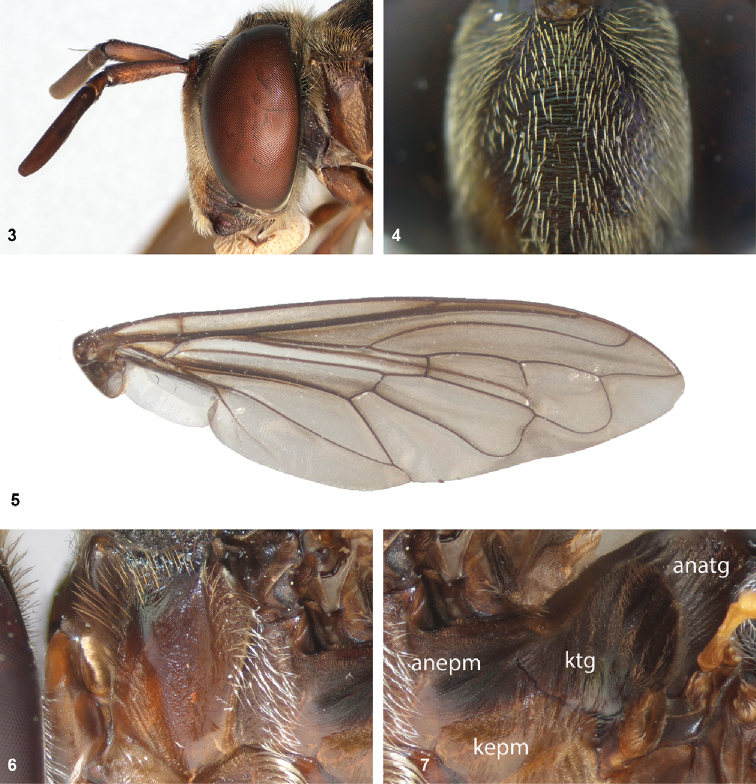
**3** Head of *Peradoncostaricensis* sp. nov. in lateral view. Note ventrally produced gena and oral margin **4** Face of *Peradonbidens* in frontal view. Note transversely wrinkled median vitta, a character found in most *Peradon* species **5** Wing of *Peradonbidens*. Note posterior appendix of vein R_4+5_ and widely rounded postero-apical corner of cell r_4+5_**6** Anepisternum of *Peradonbidens*. Note extensively bare median area **7** Thoracic sclerites of *Peradonbidens* in lateral view. Note flat and bare katepimeron with wrinkled texture (the wrinkles extend from similar wrinkles om the katatergum). Abbreviations: Anepm = anepimeron; anatg = anatergum; ktg = katatergum; kepm = katepimeron.

The head shape is quite characteristic due to the ventrally produced gena and oral margin (Fig. 3). The face is pilose, except for a narrow median bare vitta, which has a characteristic transversely wrinkled texture (Fig. 4), only lacking in some smaller species of the *flavofascium*-group and in *P.aurifascia* (Hull, 1944). The antennae are longer than the distance between the antennal fossa and the anterior oral margin, and in most species the basoflagellomere is longer than the scape.

In most *Peradon* species the postpronotum is pilose, as in most other Microdontinae. In the following species, however, the postpronotum is bare: *P.aurigaster* (Hull, 1941), *P.ballux* sp. nov., *P.brevis* sp. nov., *P.flavofascium* (Curran, 1925), and *P.surinamensis* sp. nov. Sternite 1 is always bare. In the male genitalia, the phallus is not or little projecting beyond the apex of the hypandrium, and it is shallowly furcate, with both processes approximately equally long (Figs 256–283).

Intraspecific variation in morphology and colouration in *Peradon* is wide, as demonstrated by the fact that the genus is keyed out in six different couplets in the keys to genera of Microdontinae by Reemer and Ståhls (2013a) and Reemer (2014). Despite this, the genus can be recognized by the following unique combination of five external characters:

• wing vein R_4+5_ with posterior appendix (Fig. 5);

• postero-apical corner of cell r_4+5_ widely rounded (Fig. 5);

• anepisternum extensively bare medially (Fig. 6);

• katepimeron flat and bare with wrinkled texture (Fig. 7);

• vertex more or less flat (Fig. 3).

Some *Peradon* species with a constricted abdomen (such as *P.diaphanus* (Sack) and *P.oligonax* (Hull)) may be confused with species of *Rhopalosyrphus* Giglio-Tos s.l. as defined by Reemer and Ståhls (2013a). In the *Rhopalosyrphus* species involved, however (especially *R.oreokawensis* Reemer, 2013 and two undescribed species from Brazil and Mexico), the basoflagellomere is shorter than the scape, a character which in the genus *Peradon* is only known from *P.notialus* sp. nov., a small species with an oval abdomen. Besides, the very long and slender dorsal process of the male phallus clearly distinguishes all *Rhopalosyrphus* from all *Peradon*. In case of doubt, the structure of the male genitalia is always decisive.

The three species groups defined by Reemer and Ståhls (2013a) can be identified using the characters mentioned in the key in the present paper. In addition, the male genitalia are useful in distinguishing between the *bidens* and *flavofascium* groups, in case of doubt. In the species of the *bidens* group, the surstylus is narrow with a rounded apex (Figs 265–275), whereas in the species of the *flavofascium* group it is wider and more or less rectangular at the apex (Figs 276–279). This character was decisive for assigning *P.palpator* sp. nov. to the *flavofascium* group, even though the golden or silvery pile on tergite 4 characteristic of the other species in this group is missing in that species.

See Reemer and Ståhls (2013a) for a key to genera of Microdontinae of the world or Reemer (2014) for a key to the Neotropical genera. This genus is also included in the key to genera of Syrphidae of the Brazilian Amazon by Miranda (2017).

### Identification key to *Peradon* species

**Table:** 

1	Wing vein R_4+5_ with posterior appendix (Fig. 5), posteroapical corner of cell r_4+5_ widely rounded (Fig. 5), anepisternum extensively bare (Fig. 6), katepimeron flat, bare and with wrinkled texture (Fig. 7), vertex flat (Fig. 3)	***Peradon* 2**
–	Other combination of characters	**other genera of Microdontinae**
2	Abdomen constricted basally: widest point beyond posterior margin of tergite 2, usually around transition between tergites 3 and 4 (Figs 97–111)	***trivittatus* group 5**
–	Abdomen more or less oval: widest point at posterior margin of tergite 2, sometimes with tergite 3 ca. as wide (Figs 112–148)	**3**
3	Basoflagellomere more than twice as long as scape (Fig. 9). Smaller species: body length 7–10 mm	***flavofascium* group (*P.palpator*) 31**
–	Basoflagellomere less than twice as long as scape (Fig. 10). Small or large species	**4**
4	Tergites without conspicuous golden or silvery white pilosity. If with short, inconspicuous golden pile on abdomen, then face entirely yellow. Body length usually >10 mm	***bidens* group 14**
–	Tergites 3 and 4 (and 5 in females) at least laterally or posteriorly with conspicuous long golden or silvery white pilosity, which may be visible only under certain viewing angles (Figs 11, 12). Face always at least partly black or brown. Body length usually <11 mm	***flavofascium* group 31**
***trivittatus* group**		
5	Mesoscutum posteromedially with triangular area of thick, golden, appressed pile (Figs 13–15)	**6**
–	Mesoscutum without triangular area of thick golden, appressed pile	**10**
6	Pile on scutellum erect, much thinner and more yellowish than the pile on the triangular area on mesoscutum (Fig. 16). Male genitalia as in Fig. 256	***P.fenestratus* (Hull)**
–	Pile on scutellum appressed, thick and golden, similar to golden triangular area on mesoscutum (Figs 13–15)	**7**
7	Lateral and anterior margins of mesoscutum without golden pile (at most with inconspicuous silvery pile) (Figs 13, 14). Posterior margin of tergites 2 and 3 without fasciae of golden pile (Fig. 18)	**8**
–	Lateral and anterior margins of mesoscutum with golden pile (Fig. 15). Posterior margin of tergites 2 and 3 with fascia of golden pile (Fig. 17)	**9**
8	Triangular area of golden pile on mesoscutum wider than long, restricted to posterior half of mesoscutum (Fig. 13). Male: ratio of width of vertex to width of head approximately 1:4.3 (Fig. 19); tergite 2 with lateral margins rather evenly diverging from anterior margin towards posterior margin (Fig. 21); genitalia as in Fig. 257. Female: unknown	***P.aureoscutus* (Hull)**
–	Triangle of golden pile on mesoscutum longer than wide, reaching anterior half of mesoscutum (Fig. 14). Male: ratio of width of vertex to width of head approximately 1:3.3 (Fig. 20); tergite 2 with lateral margins in anterior half more or less parallel and diverging in posterior 1/2 to 1/3 (Fig. 22); genitalia as in Fig. 258. Female: Figs 102, 153–156	***P.aureus* (Hull)**
9	Male: tergite 2 parallel-sided (Fig. 23); sternite 4 on anterior 1/3 with bulging median tubercle on anterior 1/3 with long pile (Fig. 25). Female: tergite 2 slightly and evenly diverging towards posterior margin (Fig. 101); sternite 3 strongly arched posteriorly, exposing a wide yellowish membrane between its posterior margin and the straight anterior margin of sternite 4 (Fig. 27)	***P.trilinea* (Hull)**
–	Male: tergite 2 constricted or clearly widened posteriorly (Fig. 24); sternite 4 evenly convex and with short pile (Fig. 26). Female: tergite 2 more or less parallel-sided on anterior 3/4, and abruptly diverging in posterior 1/4 (Fig. 98); sternite 3 with posterior margin set closely to anterior margin of tergite 4, no transitional membrane visible	***P.trivittatus* (Curran)**
10	Wing partly infuscated dark brown to black (Figs 105–109). Mesonotal transverse suture without fascia of golden pile (Figs 105–109, 209)	**11**
–	Wing not infuscate, only partly yellow coloured on anterior half (Figs 30, 110, 111). Mesonotal transverse suture with narrow fascia of golden pile, widely interrupted medially (Fig. 31). Male genitalia as in Fig. 264	***P.oligonax* (Hull)**
11	Tergite 2 as wide as long or wider than long (Figs 33–35)	**12**
–	Tergite 2 longer than wide (Fig. 32)	***P.diaphanus* (Sack)**
12	Vena spuria yellow. Female: costal cells and anterobasal veins yellow (Figs 28, 29)	***P.elongatus* (Hull)**
–	Wing without yellow veins or cells (Fig. 105, 106)	**13**
13	Tergite 2 clearly wider than long (Fig. 34). Cell bm entirely clear (Figs 105, 206)	***P.hermetia* (Curran)**
–	Tergite 2 ca. as wide as long (Fig. 35). Cell bm blackish in posterior half (Figs 106, 211)	***P.hermetoides* (Curran)**
***bidens* group**		
14	Tergites entirely black or dark brown (Figs 112, 113, 116, 119, 122–128)	**15**
–	At least tergite 2 partly pale coloured (Figs 114, 115, 118, 121, 129–135)	**25**
15	Wings without any trace of yellow, entirely clear or more less uniformly smoky black (Figs 36, 112–117)	**16**
–	Wings with (sometimes very) small or larger yellow or whitish macula or fascia, which may be confined to small part of cell r_4+5_ or anterobasal cells (view against dark background) (Figs 37–47, 120–128)	**19**
16	Vertex produced dorsally (Fig. 48). Short and sturdy species with thick hind femur and tibia (Fig. 49). Mimic of *Trigona* bees	***P.satyricus* Reemer**
–	Vertex not produced (Fig. 3). More slender species, hind femur and tibia not so thick (Fig. 50)	**17**
17	Face entirely yellow (Fig. 51). Mesoscutum with fascia of golden pile along transverse suture (can be very narrow, look closely under at least 20× magnification) (Figs 114–118). Body length usually 12.5–14 mm, but dwarf specimens may occur	**18**
–	Face broadly black medially (Fig. 52). Mesoscutum without fascia of golden pile along transverse suture (Fig. 113). Body length 10–12 mm. Male genitalia as in Fig. 265	***P.sciarus* Reemer**
18	Wing cell bm almost entirely microtrichose, except narrowly bare at base (Fig. 61). Fascia of golden pile along transverse suture widely interrupted medially (check under different viewing angle to be certain) (Fig. 64). Wing never with any traces of yellow (Figs 36, 116)	***P.bidens* (black morph)**
–	Wing cell bm bare on basal 3/4, except for narrow median strip of microtrichia over most of the length of cell (Fig. 62). Fascia of golden pile along transverse suture uninterrupted (check under different viewing angle to be certain) (Fig. 64). Wing either without any traces of yellow, or only with yellow veins around cell br and yellow vena spuria (Figs 117, 118)	***P.costaricensis* (dark specimens)**
19	Pale wing marks extensive, present also on basal half of wing (Figs 41, 42)	**20**
–	Pale wing marks confined to apical half of wing (Figs 37, 43–46)	**21**
20	Wing cell _2+3_ with yellow mark reaching beyond spur in vein r_4+5_ (Fig. 41). Dark wing cloud situated at apex of cell r_1_ (Fig. 41)	***P.flavipennis* (Curran)**
–	Wing cell r_2+3_ with yellow mark not reaching spur in vein r_4+5_ (Fig. 42). Dark wing cloud situated well before apex of cell r1 (Fig. 42)	***P.normalis* (Curran) (SUR-17b morph)**
21	Alula entirely microtrichose	***P.niger* (Williston)**
–	Alula largely bare medially, microtrichose only along margins	**22**
22	Wing cell br partly bare posteriad of vena spuria (Fig. 66)	**23**
–	Wing cell br entirely microtrichose (Fig. 65)	**24**
23	Mesonotal transverse suture with interrupted fascia of golden pile (as in Fig. 63). Female: tergite 4 without patch of greyish pruinescence (view from frontal angle) (Fig. 53); tergite 3 on basal 1/3 with greyish pruinescence (view from frontal angle) (Figs 57, 59)	***P.normalis* (Curran)**
–	Mesonotal transverse suture without fascia of golden pile (as in Fig. 113). Female: tergite 4 with large basomedian patch of greyish pruinescence (view from frontal angle) (Fig. 56); tergite 3 without greyish pruinescence basally	***P* . cf.sciarus Curran female**
24	Yellow on wing confined to narrow part along vena spuria on basal half of cell r_4+5_ (Fig. 37). Female unknown	***P.bispina* (Hull)**
–	Yellow on wing mostly on apical half of cell r_4+5_ and often extending into cells r_2+3_ and r_1_ (Figs 44, 45). Female with large basomedian patch of grey pruinescence on tergite 4 (Fig. 55)	***P.pompiloides* sp. nov.**
25	Tergite 3 almost entirely black or dark brown, at most lateral margins yellowish (Figs 79–81, 132–134)	**26**
–	Tergite 3 largely pale coloured (Figs 114, 115, 121, 129–131)	**28**
26	Tergite 2 yellow only along lateral margins (Fig. 79). Wing cell bm extensively bare (Fig. 62)	***P.costaricensis* sp. nov.**
–	Tergite 2 more extensively yellow (Fig. 80, 81). Wing cell bm with small bare area basally (as in Fig. 61)	**27**
27	Mesoscutum without fascia of golden pile along transverse suture (Figs 134, 135). Tergite 4 reddish yellow along apical margin (Fig. 71). Bare median facial vitta smooth and shining (Fig. 70). Male genitalia as in Fig. 273	***P.aurifascia* (Hull)**
–	Mesoscutum with narrow fascia of golden pile along transverse suture, sometimes medially interrupted (Figs 132, 133). Tergite 4 entirely dark brown (Figs 132, 133). Bare median facial vitta with transversely wrinkled texture (as in Fig. 4). Male genitalia as in Fig. 274	***P.luridescens* (Walker)**
28	Wings entirely greyish to blackish, without yellow areas (Figs 114, 115)	***P.bidens* (Fabricius) (red morph)**
–	Wings partly yellowish (Figs 121, 129–131)	**29**
29	Wing with yellow areas anterobasally (Figs 129–131)	**30**
–	Wing with yellow areas subapically (Fig. 121)	***P.normalis* (Curran) (red morph)**
30	Median fascia of golden pile along mesonotal transverse suture widely interrupted medially (as in Fig. 63). Face darkened medially (as in Fig. 52). Scutellum blackish, except yellow along apical margin (Fig. 73)	***P.angustus* (Macquart)**
–	Median fascia of golden pile along mesonotal transverse suture complete (as in Fig. 64). Face entirely yellow (as in Fig. 51). Scutellum entirely yellow (Fig. 72)	***P.angustiventris* (Macquart)**
***flavofascium* group**		
31	Basoflagellomere more than twice as long as scape (Fig. 9). Smaller species: body length 7–10 mm	***P.palpator* sp. nov.**
–	Basoflagellomere less than twice as long as scape (Fig. 10). Small or large species	**32**
32	Tergite 4 silvery white pilose (Figs 12, 138–140). Wing cell c bare on posterobasal 2/5 to 1/2. Wing sooty grey with yellow macula in basal half of cell r_4+5_, extending anteriorly into cell r_2+3_ and posteriorly into cell dm (more extensive in female than in male) (Figs 74–77)	**33**
–	Tergite 4 silvery or golden pilose (Figs 11, 143–148). Wing cell c bare on posterobasal 1/4 or less. Yellow markings on wing (if present) in most cases not as described above, except in *Peradonaurigaster* with similar markings	**34**
33	Length of scutellar calcars clearly less than 1/5 of length of scutellum (Fig. 90). Male: yellow wing mark extends anteriorly to cell r_1_ (Fig. 74); alula entirely microtrichose, although medially less densely than along margins; genitalia as in Fig. 277 (but see notes in species account). Female: face convex in profile (Fig. 88); mesonotum with wide posterior fascia of golden pile, which is medially connected with the fascia on the transverse suture (Fig. 92); alula medially, at most, with 30% of surface bare	***P.flavofascium* (Curran)**
–	Length of scutellar calcars at least 1/5 of length of scutellum (Fig. 91). Male: yellow wing marking does not reach cell r1 (Fig. 76); alula widely bare medially, only narrowly microtrichose along margins; genitalia as in Fig. 278 (but see notes in species account). Female: face flat in profile (Fig. 89); mesonotum with narrow posterior fascia of golden pile, which is not medially connected with the fascia on the transverse suture (Fig. 93); alula only narrowly microtrichose along margins	***P.surinamensis* sp. nov.**
34	Basoflagellomere as long as scape (male) or shorter than scape (female). Alula entirely microtrichose. Mesonotum with narrow median fascia of silvery white pile (Fig. 94). Postpronotum pilose. Male: vena spuria pale yellow, costal veins orange brown, other veins blackish (Fig. 82). Female: wing colouration similar to male, but with wider yellow areas around veins (Fig. 83)	***P.notialus* sp. nov.**
–	Basoflagellomere longer than scape. Alula largely bare, only microtrichose along margins. Mesonotum with fasciae of golden pile (Figs 95, 96). Postpronotum pilose or bare	**35**
35	Mesonotum with anterior and median fascia of golden pile fused into a single wide fascia, which is connected to the posterior fascia by a wide median vitta (Fig. 96). Postpronotum bare. Small and sturdy species	***P.brevis* sp. nov.**
–	Mesonotum with anterior and median fascia separated by fascia of black pile; without median vitta (Fig. 95). Postpronotum bare or pilose	**36**
36	Males	**37**
–	Females	**39**
37	Wing entirely dusky grey, without yellow parts (view against a dark background) (Fig. 84); basal thickened part of costal vein dark brown to blackish. Postpronotum pilose	***P.chrysopygus* (Giglio-Tos) male**
–	Wing with small or large yellow macula, at least on basal half of cell r_2+3_ (Figs 78, 80); basal thickened part of costal vein yellow. Postpronotum bare	**38**
38	Yellow wing markings confined to cells sc, r_1_ and r_2+3_ (Fig. 80)	*P.ballux* sp. nov. male
–	Yellow wing markings include cell sc, basal half of cells r_1_, r_2+3_ and r_4+5_ and apical half of cell dm (Fig. 78)	***P.aurigaster* (Hull) male**
39	Wing as in Fig. 85: with large yellow part basally; cell r_2+3_ dark medially and with yellow parts basally and proximally; cell r_4+5_ largely dark. Postpronotum pilose. Body length 8–11 mm	***P.chrysopygus* (Giglio-Tos) female**
–	Wing cell r_2+3_ with yellow marking medially; sometimes also r_4+5_ partly yellow (Fig. 79). Postpronotum bare. Body length < 9 mm	**40**
40	Tergite 2 with conspicuous golden pile laterally. Wing cell r_4+5_ partly yellow	***P.aurigaster* (Hull) female**
–	Tergite 2 with inconspicuous silvery pile laterally. Wing cell r_4+5_ without yellow colouration	*P.ballux* sp. nov. female^*^

### Species accounts

#### Peradon angustiventris

Taxon classificationAnimaliaDipteraSyrphidae

(Macquart)

6FED6896-3AB9-5AB6-8B1D-9CA5F54615E6

Figs 39 , 130 , 131 , 149 , 150 , 275

Aphritis angustiventris Macquart, 1855: 105. Holotype ♂: South America (OUMNH) [examined].
Microdon angustiventris (Macquart): Thompson et al. 1976: 63.
Peradon angustiventris (Macquart): Reemer and Ståhls 2013a: 145; Reemer 2014: 42.

##### Studied type specimens.

South America • 1 ♂, holotype of *Aphritisangustiventris* Macquart; OUMNH.

Label 1 (small, round, red-bordered): “Holo- / type”; label 2: “A. angustiventris / Ex coll. Bigot”; label 3: “Aphritis / angustiventris / [male sign] Macq.”. Coll. OUMNH.

##### Additional specimens.

Brazil • 1 ♂; Amapá, Oiapoque Rancho Km-9, Varredura; 03°47'53"N, 51°48'03"W; 15 Nov. 2014; J.A. Rafael & F.F. Xavier leg.; INPA • 1 ♀; Amazonas, Barcelos, Rio Demeni, Pirico; 0°19'30"S, 62°47'21"W; Aug. 2008; A. Silva & R. Machado leg.; INPA • 1 ♂; Manaus; Aug. 1959; C. Elias leg.; UFPR • 1 ♂; R. Ducke; 23 Mar. 1982; J.A. Rafael leg.; INPA • 1 ♀; Amazonas, Manaus, AM010 km 50; 02°44'13"S, 59°54'32"W; 5–8 Feb. 2005; F.F. Xavier & G.M. Lourido leg.; (INPA) • 1 ♂; Novo Airão, AM 352, Ramal Km 10; 02°42'56.5"S, 60°56'26.7"W; 28–29 Aug. 2011; J.A. Rafael, D. Takiya & J.T. Câmara leg.; INPA • 1 ♂; Novo Airão, AM 352, Ramal Km 10; 02°42'56.5"S, 60°56'26.7"W; 29 Aug. 2011; J.A. Rafael, D. Takiya & J.T. Câmara leg.; INPA • 1 ♂; Novo Airão, AM 352, Ramal Km 10; 02°42'56.5"S, 60°56'26.7"W; 29–30 Aug. 2011; J.A. Rafael, D. Takiya & J.T. Câmara leg. (INPA) • 1 ♂; Faz. Taperinha, prox. Santarem, PA; 29 Dec. 1967–9 Jan. 1968; Exp. Perm. Amaz. leg.; MZUSP • 1 ♂; Rondonia, 62 km SE Ariquemes; 5–16 Nov. 1996; W.J. Hanson leg.; LACM.

Ecuador • 1 ♂; Napo, Tena.; 500 m a.s.l.; 11–28 Apr. 1976; M. Cooper leg.; NHMUK (“B.M. 1976-290”).

Guyana • 1 ♂; Bartica; 11 May 1901; CNC • 1 ♂; Kartabo; Sep. 1922; M.D. Haviland leg.; NHMUK.

Suriname • 1 ♀; Republiek; 30 May 1963; P.H. van Doesburg Jr. leg.; RMNH • 1 ♂; Zanderij; 11 May 1963; P.H. van Doesburg Jr. leg.; RMNH • 1 ♂; Colakreek; 9 Mar. 2006; M. Reemer leg.; RMNH • 1 ♂; Colakreek; 30 Mar. 2006; M. Reemer leg.; RMNH.

##### Diagnosis.

Body length: male 12–15 mm, female 14–16 mm. A large species with elongate, not constricted abdomen. Tergites 2 and 3 are yellowish brown, while tergite 4 may be orange brown or dark brown. The wings are yellow anterobasally, the fascia of golden pile along the mesonotal transverse suture is complete (not interrupted medially), and the face is entirely yellow. Male genitalia as in Fig. 275.

##### Notes.

In the present concept, this species only differs from *P.luridescens* by tergite 3 being largely yellowish instead of dark brown. Several specimens of both *P.angustiventris* and *P.luridescens* are represented in material from Suriname in the RMNH collection (see images in Reemer 2014), and these specimens fall into two discrete groups based on this character (tergites 3 and 4 entirely yellowish brown in *P.angustiventris*, tergites 3 and 4 blackish brown in *P.luridescens*). In Suriname, *P.angustiventris* and *P.luridescens* do not seem to co-occur, with *P.angustiventris* found in the savannah areas of the old coastal plain (localities Colakreek, Republiek, Zanderij), and *P.luridescens* in the bauxite containing plateaus in the interior at higher elevation (Brownsberg, Nassau Mts., Lely) (Reemer 2014). However, specimens which could be considered intermediates are known from other countries. In these specimens, tergite 3 is yellowish brown whereas tergite 4 is blackish brown. Here, such specimens are preliminarily assigned to *P.angustiventris*, because they agree with the type specimen in colouration of tergite 3. This preliminary solution is unsatisfactory, as colouration of the abdomen is variable in these intermediate specimens. Possibly, the intermediates represent a third species or even a species complex, but there are too few specimens available to make a decision on this. Another possibility is that *P.angustiventris* and *P.luridescens* are the extreme ends of one highly variable species, but the fact that specimens from within one country (Suriname) can clearly be assigned to two different groups without significant character variation is here taken as a clue that this is not the case. The results of analyses of the barcodes of a few specimens do not shed light on this matter.

##### Distribution.

Known from the Brazilian Amazon region, Ecuador, Guyana, and Suriname.

#### Peradon angustus

Taxon classificationAnimaliaDipteraSyrphidae

(Macquart)

C6F167AD-F0B9-5CB1-B3F2-C21D1F1599C8

Figs 40 , 129

Aphritis angustus Macquart, 1846: 250. Type locality: French Guiana, Cayenne (type lost). Neotype ♀: Surinam, Mopentibo (RMNH) [examined].
Microdon angustus (Macquart): Thompson et al. 1976: 63.
Peradon angustus (Macquart): Reemer and Ståhls 2013a: 145; Reemer 2014: 42 [neotype designation].

##### Studied type specimens.

Suriname • 1 ♀, neotype of *Aphritisangustus* Macquart [designated by Reemer 2014]; Commewijne, Mopentibo, near Meerzorg; 05°47'57"N, 55°07'05"W; 19 Apr. 2006; M. Reemer leg.; RMNH.

##### Additional specimens.

Suriname • 1 ♀; Peperpot; 21–28 Mar. 2006; M. Reemer leg.; RMNH.

##### Diagnosis.

Body length: female 14 mm. A large species with elongate, not constricted abdomen. The tergites are reddish with a median blackish vitta on tergites 3 and 4. The wings are yellow anterobasally and blackish along the margins. The fascia of golden pile along the transverse suture of the mesonotum is widely interrupted medially. The face is darkened medially. This species is morphologically very similar to *P.bidens*, from which it differs by the partly yellow wings and the dark median vitta on tergites 3 and 4. It differs from *P.angustiventris* and *P.luridescens* by the widely interrupted fascia of golden pile along the mesonotal transverse suture.

##### Notes.

The type of this taxon is considered lost, and Reemer (2014) designated a neotype.

##### Distribution.

Known from French Guiana and Suriname.

#### Peradon aureoscutus

Taxon classificationAnimaliaDipteraSyrphidae

(Hull)

8D43E316-D5BE-5F3F-9975-EFF483C58886

Figs 13 , 19 , 21 , 104 , 151 , 152 , 257

Microdon aureoscutus Hull, 1943: 709. Holotype ♂: Amazon (NHMUK) [examined]; Thompson et al. 1976: 63.
Peradon aureoscutus (Hull): Reemer and Ståhls 2013a: 146.

##### Studied type specimens.

Amazon Region • 1 ♂, holotype of *Microdonaureoscutus* Hull; Amazon; NHMUK. Label 1 (small, round, red-bordered): “Holo- / type”; label 2: “Amazon / 6653”; label 3 (red): “Holotype / Microdon / aureoscutum / Hull”. According to Hull (1943) this specimen was collected by H.W. Bates.

##### Diagnosis.

Body length: male 11 mm. The triangle of golden pile on the mesoscutum places this species in a group together with *P.aureus*, *P.trilinea* and *P.trivittatus*. From the latter two species, *P.aureoscutus* differs by the absence of golden pile along the anterior and lateral margins of the mesoscutum. The species differs from *P.aureus* by the triangle of golden pile on the mesoscutum being wider than long and restricted to the posterior half of the mesoscutum. In the male, the ratio vertex width:head width is approximately 1:4.3 (Fig. 19), and the lateral margins of tergite 2 are rather evenly diverging posteriad. Male genitalia as in Fig. 257. The female is unknown.

##### Notes.

See *P.aureus*.

##### Distribution.

Known from the Brazilian Amazon region.

#### Peradon aureus

Taxon classificationAnimaliaDipteraSyrphidae

(Hull)

A4F28091-462C-52EF-8195-341AC3DC8D13

Figs 14 , 20 , 22 , 102 , 103 , 153–156 , 258

Microdon aureus Hull, 1944b: 35. Holotype ♀: Ecuador (MCZ) [examined]; Thompson et al. 1976: 63.
Peradon aureus (Hull): Reemer and Ståhls 2013a: 146; Marín-Armijos et al. 2017: 175.

##### Studied type specimens.

Ecuador • 1 ♀, holotype of *Microdonaureus* Hull; Jatun Yacu, Rio Napu watershed, Oriente; 700 m a.s.l.; Jan.-Jul. 2003; C. McIntyre leg.; MCZ.

Label 1: “Jatun Yacu / Rio Napu / watershed / 700 m”; label 2: “Oriente / Ecuador”; label 3: “Wm. C. / Mcintyre”; label 4 (red): “M.C.Z. / Type / 23807”; label 5: “Microdon aureus Hull n. sp. holotype”; label 6: “Jan.-July 2003 / MCZ Image / database”; label 7: “MCZ-ENT /00023807”.

##### Additional specimens.

Ecuador • 3 ♂; Pompeya, Napo R., Pastaza; 14–22 May 1965; L. Pena leg.; CNC • 1 ♀; Coca, Napo R., Napo; 250 m a.s.l.; May 1965; L. Pena leg.; CNC • 1 ♂; Sucumbios, Sacha Lodge; 00.5S, 76.5W; 290 m a.s.l.; Jul. 1994; P. Hibbs leg.; LACM • 1 ♂; Napo Prov., Limoncocha; 16 Jun. 1977; D.L. Vincent leg.; USNM.

##### Diagnosis.

Body length: male 11–14 mm, female 16–19.5 mm. The triangle of golden pile on the mesoscutum places this species in a group together with *P.aureoscutus*, *P.trilinea* and *P.trivittatus*. From the latter two species, *P.aureus* differs by the absence of golden pile along the anterior and lateral margins of the mesoscutum. The species differs from *P.aureoscutus* in the triangle of golden pile on the mesoscutum being longer than wide and reaching the anterior half of the mesoscutum. In the male, the ratio vertex width:head width is approximately 1:3.3 (Fig. 14), and the lateral margins of tergite 2 are more or less parallel in the anterior half and diverging in the posterior 1/2 to 1/3. The female is figured in Fig. 102. As the female of *P.aureoscutus* is unknown, it is uncertain whether the characters described for the males of both species also apply to the females.

##### Notes.

This taxon is very similar to *P.aureoscutus*. Although both taxa were described by F.M. Hull, he apparently was unaware of this similarity, as Hull (1944b) only noted that it was ‘related distantly to *beebei*’ (a species nowadays placed in the genus *Pseudomicrodon* Hull, 1937).

##### Distribution.

Known only from Ecuador.

#### Peradon aurifascia

Taxon classificationAnimaliaDipteraSyrphidae

(Hull)

DFD7CF93-8EE0-5630-A8EE-1D0FCC3D3AD8

Figs 38 , 70 , 71 , 134 , 135 , 157 , 160 , 273

Microdon aurifascia Hull, 1944c: 245. Holotype ♂: Brazil, São Paulo (NHMUK) [examined]; Thompson et al. 1976: 63.
Peradon aurifascia (Hull): Reemer and Ståhls 2013a: 145.

##### Studied type specimens.

Brazil • 1 ♂, holotype of *Microdonaurifascia* Hull; Sao Paulo, Alto da Serra; 12 Mar. 1912; G.E. Bryant leg.; NHMUK.

Label 1 (small, round, red-bordered): “Holo- / type”; label 2: “Alto da Serra, / Sao Paulo, / Brazil. / G.E. Bryant. / 12.III.1912.”; label 3 (red): “Holotype / Microdon / aurifascia / Hull”. Coll. NHMUK.

##### Additional specimens.

Argentina • 1 ♀; Misiones, Leandro N. Alem; 24 Feb. 1951; Duret leg.; MNHN.

Brazil • 1♀; Nova Teutonia; 14 Oct. 1940; F. Plaumann leg.; CNC • 1 ♂; São Paulo, M. das Cruses; Aug. 1939; M. Carrera leg.; CNC • 1 ♂; Est. Biol. Boracéia, Salesópolis, S.P.; 16 Sep. 1993; W. Wilms leg.; CSCA • 1 ♀; Est. Biol. Boracéia, Salesópolis, S.P.; 19 Sep. 1965; Rabello leg.; MZUSP • 1 ♀; Floresta da Tijuca; Mar. 1964; C.A.C. Seabra leg.; MZUSP.

##### Diagnosis.

Body length: male 12.5–13 mm, female 14–15 mm. A large species with elongate, unconstricted abdomen. Male with wing yellowish (Fig. 38, 134), female with wing yellow only anterobasally and with grey subapical cloud (Fig. 135). The abdomen is dark brown, except for a pair of yellow maculae on tergite 2. The species resembles *P.angustiventris* and especially *P.luridescens* but differs from those by the absence of a fascia of golden pile along the mesonotal transverse suture. It also differs from most other *Peradon* species (except for some small species of the *flavofascium* group) in the smooth and shining bare median facial vitta (Fig. 70). Male genitalia as in Fig. 273.

##### Distribution.

Known from the Misiones province in northeastern Argentina and from the Santa Catarina and São Paulo states in southern Brazil.

#### Peradon aurigaster

Taxon classificationAnimaliaDipteraSyrphidae

(Hull)

39D1F01A-79B6-5F81-B3C8-991A24EB03E7

Figs 2 , 11 , 78 , 79 , 143 , 144 , 161 , 164 , 283

Microdon aurigaster Hull, 1941: 160. Holotype ♂: Bolivia (MCZ) [examined]; Thompson et al. 1976: 63.
Peradon aurigaster (Hull): Reemer and Ståhls (2013a: 160).

##### Studied type specimens.

Bolivia • 1 ♂, holotype of *Microdonaurigaster* Hull; Prov. Sara, Steinbach; MCZ.

Label 1: “Bolivia / Prov. Sara / Steinbach”; label 2: “M.C.Z. / Type / 23795”; label 3: “Microdon/ aurigaster / Hull n. sp.”; label 4: “Jan. –July 2003 / MCZ Image / database”; label 5: “MCZ-ENT / 00023795”. Coll. MCZ.

##### Additional specimens.

Bolivia • 1 ♂; Mapiri; RMNH.

Brazil • 1 ♀; Pará, Belem; 20 Apr. 1967; Y. Sedman leg.; CNC • 1 ♂; Rondônia, Vilhena; 12°65'55"S, 60°22'18"W; 25 Apr. 2006; J.A. Rafael & F.F. Xavier leg.; INPA • 1 ♂; Dist. Fed. Planaltina, cerrado; 1000 m a.s.l.; 24 Sep.–6 Oct. 1985; Scott E. Miller leg.; USNM (USNMENT01371107) • 1 ♀; Dist. Fed. Planaltina, cerrado; 1000 m a.s.l.; 6–25 Oct. 1985; Scott E. Miller leg.; USNM (USNMENT01371106) • 1 ♀; Mato Grosso; 12°50'S, 51°47'W; 26 Feb. 1968; O.W. Richards leg., R.S. & R.G.S. Exped. B.M. 1968-260; NHMUK • 1 ♀; West border Mato Grosso; May 1931; R.C. Shannon leg.; USNM (red label “Paratype”, see Notes).

Peru • 1 ♀; Tingo Maria, Rio Huallaga; 700 m a.s.l.; Nov. 1947; Weyrauch leg.; CNC • 1 ♀; San Martin, around San Ruque de Cumbaza; 6°23'4.96"S, 76°25'53.47"W; 15–31 Jan. 2015; T. Faasen leg.; RMNH.

##### Diagnosis.

Body length: male 7.5–9.5 mm, female 9.5–10 mm. The conspicuous golden pile on tergite 4 places this species in the *flavofascium* species group. Within this group, this species is distinguished by the combination of the following characters: basoflagellomere longer than scape, postpronotum bare, wing cell c bare on posterobasal 1/4 or less, alula largely bare, wing cell r_4+5_ partly yellow. Male genitalia as in Fig. 283.

##### Notes.

A Brazilian female specimen from the USNM collection carries a red label stating “Paratype” (see Additional specimens). As Hull (1941) based his description on a single specimen and does not mention additional material, this specimen cannot be considered a paratype. Variation in colouration in this species is considerable. Based on the present material, it is not possible to assess the taxonomic relevance of this variability, but it seems possible that *P.aurigaster* as defined here represents a species complex.

##### Distribution.

Known from Bolivia, Brazil (Pará, Mato Grosso, Rondônia), and Peru.

#### Peradon ballux

Taxon classificationAnimaliaDipteraSyrphidae

Reemer sp. nov.

DFF7C780-1E8A-5457-820C-8B63FEFD22BF

http://zoobank.org/39E2E7FC-5D83-4F86-8792-70FB8B1E1043

Figs 80 , 95 , 145 , 165–167 , 282

##### Type material.

***Holotype*.** Argentina • 1 ♂, holotype of *Peradonballux* sp. nov.; Misiones, 5 km E Puerto Iguazo, behind Hotel Orquidaes; 6 Feb. 1992; S.A. Marshall leg.; DEBU.

Label 1: “Argentina: Misiones / 5 km E Puerto Iguazo / behind Hotel Orquidaes / 1–6.ii.1992 / S.A. Marshall”; label 2: “Peradon sp. 02 / det. M. Reemer 2016 / Voucher code MR715”.

##### Additional specimens.

Brazil • 1 ♂; Paraná, Piraquara, Mananciais da Serra, Mar; 25°29'46"S, 48°58'54"W; 6 Dec. 2007; J.A. Rafael leg.; INPA (INPA-DT0000058).

##### Description

**(based on holotype). Adult male.** Body size: 8 mm.

***Head*.** Face occupying 0.47 of head width in frontal view; black, except for pale yellow, oblique vittae laterally on ventral half; white pilosity, more golden medially. Gena black with white pilosity. Lateral oral margin weakly produced; black with white pilosity. Frons and vertex black with golden pilosity, except ocellar triangle with black pilosity. Occiput black with golden pilosity dorsally, white pilosity ventrally. Eye bare. Antennal fossa approx. as wide as high. Antenna brown. Ratio scape:basoflagellomere approximately 1:1. Basoflagellomere parallel-sided with rounded apex. Arista slender, ca. 3/5 of length of basoflagellomere.

***Thorax*.** Mesoscutum dull black; short, appressed black pile, except for narrow fasciae of pale golden pile along anterior margin, transverse suture, and posterior margin. Postpronotum brown; bare. Postalar callus brown; golden pilosity. Scutellum black with faint metallic green hue, posterior margin brownish; short yellow pilosity; with two apical calcars of ca. 1/6 of length of scutellum, with mutual distance slightly more than 1/2 length of scutellum. Pleura blackish brown. Anterior and posterior part of anepisternum separated by deep sulcus; golden pilosity anteriorly, whitish pile posteriorly, with wide bare area in between. Anepimeron entirely whitish pilose. Katepisternum white pile dorsally, bare ventrally. Other pleura bare (except for microtrichiae). Calypter whitish with yellow margin. Halter yellow.

***Wing*.** Hyaline, except for pale yellow macula in basal half of cell r_2+3_, with dark veins, except veins c and R_1_ yellow between wing base and stigmal crossvein. Wing microtrichose, except bare on posterobasal 5/6 of br, basal 3/4 of bm, anterobasal 1/4 of cup, and most of alula (only microtrichose along margins).

***Legs*.** Brownish black with faint metallic hues; white pilosity, except tarsi and tibiae ventrally dark golden pilosity.

***Abdomen*.** Elongate, widest at middle of tergite 3, although this tergite almost parallel-sided; blackish brown, except pale brown on lateral margins of tergites 3 and 4, and largely golden metallic medially on tergite 4; tergite 1 white pilosity; tergites 2 and 3 short black pilosity, except short golden to white pilosity along lateral margins; tergite 4 golden pilosity with narrow median vitta of short black pile. Sternites blackish brown; white pilosity, except sternite 1 bare.

**Female.** Unknown.

##### Diagnosis.

Body length: male 8–9 mm. The conspicuous golden pile on tergite 4 places this species in the *flavofascium* species group. Within this group, this species is distinguished by the combination of the following characters: basoflagellomere longer than scape, postpronotum bare, wing cell c bare on posterobasal 1/4 or less, alula largely bare, wing cell r_4+5_ without yellow colouration. Male genitalia as in Fig. 282.

##### Notes.

The additionally studied male specimen from Brazil (Paraná) differs from the holotype in its larger body size (9 mm), slightly smoky wings, and the presence of a pair of submedian vittae of black pile on tergite 4. Possibly, this specimen belongs to another species, which is why it is not designated as paratype. However, the number of available specimens is too low to make a decision on this.

##### Distribution.

Known from the Argentinian province Misiones and the Brazilian state of Paraná.

##### Etymology.

The Latin word *ballux* means gold-dust, and this was chosen as specific epithet in reference to the golden pilosity of tergite 4. It is to be treated as a noun in apposition.

#### Peradon bidens

Taxon classificationAnimaliaDipteraSyrphidae

(Fabricius)

C0E365F3-A285-5A14-B239-AA578304716F

Figs 4–7 , 10 , 36 , 50 , 61 , 63 , 114–116 , 168–170 , 267

Mulio bidens Fabricius, 1805: 185. Holotype ♂: South America (UZMC [examined].
Ceratophya bicolor Walker, 1857: 151. Holotype ♀: Brazil, Pará (NHMUK) [examined].
Microdon flavomarginatum Curran, 1925: 245. Holotype ♀: Peru (CU) [examined] syn. nov.
Microdon langi Curran, 1925: 341. Holotype ♂: Guyana, Kumakusa (AMNH) [examined]; Thompson et al. 1976: 65 [type deposition erroneously stated as CU] syn. nov.
Microdon bidens (Walker): Thompson et al. 1976: 63.
Microdon flavomarginatus Curran: Thompson et al. 1976: 65.
Peradon bidens (Fabricius): Reemer and Ståhls 2013a: 145; Reemer 2014: 43.
Peradon flavomarginatum (Curran): Reemer and Ståhls 2013a: 145.
Peradon langi (Curran): Reemer and Ståhls 2013a: 145; Reemer 2014: 43.

##### Studied type specimens.

South America • 1 ♂, holotype of *Muliobidens* Fabricius; UZMC. Label 1 (red): “TYPE”; label 2: “M: bidens / ‘Am:mer:Schmid”. Coll. UZMC.

Brazil • 1 ♀, holotype of *Ceratophyabicolor* Walker; Para; NHMUK. Female. Label 1 (round, red-bordered): “Holo- / type”; label 2 (round, green-bordered): “ Type”; label 3: “Para”; label 4: “Ceratophya / bicolor / Wlk.”; label 5: “bicolor Wlk”.

Peru • 1 ♀, holotype of *Microdonflavomarginatum* Curran; La Sombra to El Encanto, Putumayo Dist.; 23 Aug. 1923; Cornell University Expedition Lot 569 leg.; CU. Label 1: “La Sombra to El Encanto, Putumayo Dist., PERU, Aug. 23 ‘20”; label 2: “Cornell Univ. Expedition Lot 569”; label 3 (red): “Holotype Cornell U. No. 1732”; label 4 (red): “Type Microdon flavomarginatum Curran”; label 5: “Microdon flavomarginatum Curran Det. C.H. Curran”.

Guyana • 1 ♂, holotype of *Microdonlangi* Curran; Kumakusa; Sept. 1922; H. Lang leg.; AMNH. Label 1: “Kumakusa, Brit. Guiana, IX-1922. H. Lang”; label 2 (red): “Type *Microdonlangi* Curran”; label 3: “C.H. Curran collection, Acc. 31144”; label 4: *Microdonlangi* Curran, Det. C.H. Curran”.

##### Additional specimens red morph.

Brazil • 1 ♂; Serra do Navio, Terr. Amapá; 20 Oct. 1957; J. Lane leg.; USNM • 1 ♀; Itaituba; MZH (Frey collection).

Peru • 1 ♀; Chanchamayo; USNM • 1 ♂; Pará, Belém; 8 May 1967; Y. Sedman leg.; USNM [dwarf specimen, see Notes] • 1 ♀; Pará, Belém; 12 Aug. 1962; K. Lenko leg.; USNM.

French Guiana • 1 ♀; Alicoto-Oyapock; 14 Nov. 1969; Balachowsky-Gruner leg.; MNHN. GUYANA • 1 ♀; Georgetown; H.W.B. Moore leg.; USNM.

Peru • 1 ♀; Marcapota; RMNH.

Suriname • 1 ♀; Paramaribo; 15 Jul. 1944; D.C. Geijskes leg.; RMNH • 1 ♀; Paramaribo; 28 Jan. 1960; D.C. Geijskes leg.; RMNH • 1♂; Lely, 29 Oct.1979; G.F. Mees; RMNH • 1 ♂; Peperpot; 4 Mar. 2006; M. Reemer leg.; RMNH • 1 ♂; Peperpot; 14–21 Mar. 2006; M. Reemer leg.; RMNH.

##### Additional specimens black morph.

Brazil • 1 ♀; Amazonas, Resex Unini, Rio Unini, Lago 03 Bocas; 01°34'13"S, 62°58'54"W; 14–28 Jul. 2004; M.L. Oliveira, A. Silva F., L. Aquino leg.; INPA • 1 ♂; Serra do Navio, Terr. Amapá; 21 Oct. 1957; K. Lenko leg.; MZUSP.

Suriname • 1 ♂; Colakreek; 30 Mar. 2006; M. Reemer leg.; RMNH • 1 ♂; Mopentibo; 19 Apr. 2006; M. Reemer leg.; RMNH.

##### Diagnosis.

Body size: male (9–)12–14 mm (single dwarf specimen of 9 mm known, see Notes), female 15–16 mm. Two colour morphs are known of this species. In the red morph (corresponding with the type of *Muliobidens*) tergites 2–4 are entirely red. From other species with an entirely reddish abdomen, the red morph of *P.bidens* differs by the uniformly greyish wings, lacking any pale areas or veins. In the black morph (corresponding with the type of *Microdonlangi*) the abdomen is entirely black. The following combination of characters distinguishes it from other *Peradon* species with a black abdomen: wings uniformly greyish to blackish, vertex more or less flat, face yellow, mesoscutum with medially interrupted fascia of golden pile along transverse suture, wing cell bm entirely microtrichose, cell br largely bare. In both colour morphs, the wings of the females are generally darker than those of the males.

##### Notes.

The holotype of *Microdonflavomarginatum* Curran was studied in order to find differences with *Peradonbidens*. In this type specimen the face is widely dark brown medially, and tergite 1 is orange brown. In most specimens of *P.bidens* the face is entirely yellow and tergite 1 is blackish. However, intermediate character states occur in certain specimens. Besides these characters, no differences of taxonomic importance could be found. Therefore, *Microdonflavomarginatum* Curran syn. nov. is here considered a junior synonym of *Muliobidens* Fabricius. *Ceratophyabicolor* Walker was already synonymized with *Peradonbidens* by Reemer and Ståhls (2013a) (see also Reemer 2014).

A dwarf male specimen from Brazil (Pará, Belém, coll. USNM) measures only 9 mm. However, this specimen does not differ in any other morphological character from the other studied males.

**Figure 8.**
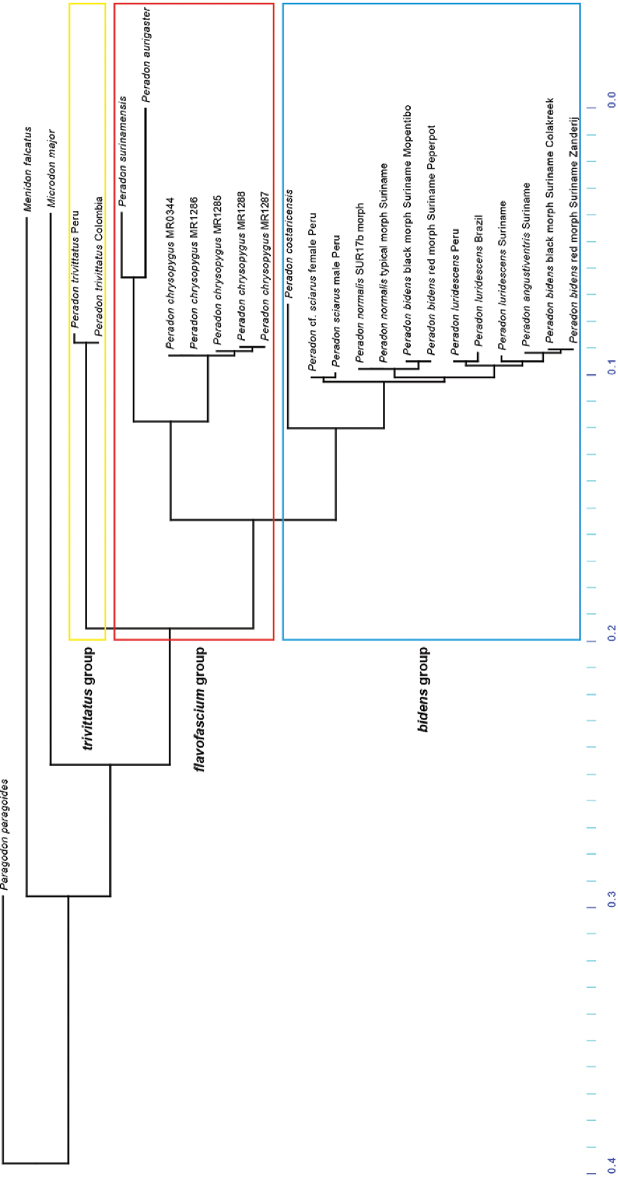
Maximum Likelihood tree of COI barcodes of *Peradon* species. The scale at the bottom is a distance scale for the number of substitutions per site. Information on the barcoded specimens can be found in Table 1.

As noted by Reemer (2014), specimens of *P.bidens* (red abdomen) are morphologically identical to specimens of *P.langi* Curran (black abdomen). Two Surinamese specimens of both taxa were included in the barcode dataset in order to find molecular clues for their taxonomic status. All three analyses (ML, parsimony and NJ, Fig. 8, Suppl. material 1: Figure S1, Suppl. material 2: Figure S2) of these data resolved two clades, both consisting of one specimen of *P.bidens* and one of *P.langi* with 100% identical barcodes. This is puzzling, but here the view is taken that all four specimens represent the same species, and two haplotypes are involved, which both happen to be represented by both a red and a black specimen. Therefore, *Microdonlangi* Curran syn. nov. is here considered as a junior synonym of *Muliobidens* Fabricius.

##### Distribution.

Amazonian. Known from northern states of Brazil (Amapá, Pará), French Guiana, Guyana, Peru and Suriname.

#### Peradon bispina

Taxon classificationAnimaliaDipteraSyrphidae

(Hull)

32DCF471-8984-5C3B-AC6C-A1D841BAD00F

Figs 37 , 128 , 171–173 , 271

Microdon bispina Hull, 1943: 707. Holotype ♂: Brazil, São Paulo (NHMUK) [examined]; Thompson et al. 1976: 64.
Peradon bispina (Hull): Reemer and Ståhls 2013a: 145.

##### Studied type specimens.

Brazil • 1 ♂, holotype of *Microdonbispina* Hull; São Paulo; H.W. Bates leg.; NHMUK. Label 1 (small, round, red-bordered): “Holo- / type”; label 2: “S. / Paulo”; label 3: “Holotype / Microdon / bispina / Hull”. Hull (1943) notes that the specimen was collected by H.W. Bates.

##### Additional specimens.

Brazil • 1 ♂; Manaus, Amazonas; 13 May 1967; Y. Sedman leg.; CNC.

##### Diagnosis.

Body length 8.5–11.5 mm. A small, slender, black species of the *bidens* group, of which only males are known. The wings are infuscate and there is a narrow yellow marking on cell r_4+5_ between the apical part of the vena spuria and vein M posterior to it (Fig. 37). This separates the species from similar species, in which the yellow wing marking is either absent or situated in a different part of the wing.

##### Notes.

In the holotype, the head of a species belonging to a different microdontine genus has been glued to the specimen: it is too large and of uncharacteristic shape for *Peradon* (lateral oral margin not produced). The specimen from Manaus is considerably smaller (8.5 mm) than the holotype from Sao Paulo (11.5 mm). The specimens are considered conspecific because of the otherwise striking similarities in morphology and colouration.

##### Distribution.

Known from Manaus and São Paulo in Brazil.

#### Peradon brevis

Taxon classificationAnimaliaDipteraSyrphidae

Reemer sp. nov.

8CBBA9D9-FC9B-5537-A3FD-75FCEECB54FF

http://zoobank.org/0C3BC2E1-DF2F-4E72-931C-1F99A157A151

Figs 81 , 96 , 148 , 174–176 , 280

##### Type material.

***Holotype*.** Ecuador • 1 ♂, holotype of Peradonbrevis sp. nov.; “Pr. Mor. -S.”; 900 m a.s.l.; 18 Aug. 1982; R. Hensen & A. Aptroot leg.; RMNH. Label 1: “ECUADOR; Pr. Mor. -S.; / Palora; 900 m; / Leg. R. Hensen et A. / Aptroot; 18-8-1982”; label 2: “Peradon sp. 04 [male symbol] / Det. M. Reemer 2016 / Voucher code MR717”; label 3: “HOLOTYPE / Peradon brevis / M. Reemer”.

##### Description

**(based on holotype). Adult male.** Body size: 7 mm.

***Head*.** Face occupying 0.5 of head width in frontal view; black, except for pale yellow, oblique yellow maculae laterally on ventral half; golden yellow pilose, with narrow bare mid line. Gena black; white pilose. Lateral oral margin clearly produced; black; white pilosity. Frons and vertex black, golden pilose. Occiput black; golden pile dorsally, white pile ventrally. Eye bare. Antennal fossa approx. as wide as high. Antenna dark brown, basal half of scape pale brown. Ratio scape:basoflagellomere approximately 1:1.2. Basoflagellomere parallel-sided with rounded apex. Arista slender, ca. 3/5 of length of basoflagellomere.

***Thorax*.** Mesoscutum dull blackish brown; golden yellow pilose, except for mediolateral patches of black pile. Postpronotum pale brown; bare. Postalar callus brown; golden yellow pilosity. Scutellum dark brown with faint metallic green hue, posterior margin paler; short golden yellow pilosity; with two apical calcars of ca. 1/6 of length of scutellum, with mutual distance approximately equal to length of scutellum. Pleura blackish brown. Anterior and posterior part of anepisternum separated by deep sulcus; golden pile anteriorly and posteriorly, with wide bare area in between. Anepimeron entirely whitish pilosity. Katepisternum white pilosity dorsally, bare ventrally. Other pleura bare (except for microtrichiae). Calypter whitish with yellow margin. Halter yellow.

***Wing*.** Hyaline with yellow veins, except for dark veins in apical 1/3 of wing and around apical 1/2 of cell br, wing membrane also somewhat infuscate around apical 1/2 of br. Wing microtrichose, except bare on posterobasal 1/4 of br, posterobasal 1/3 of bm, anterobasal 1/6 of cup, and most of alula (only microtrichose along margins).

***Legs*.** Brownish black, except ‘knees’ of mid leg (narrow part of apex of mid femur and base of mid tibia), hind tibia and basal three tarsomeres of hind leg yellowish brown; white pile on dark brown parts and yellow pile on yellowish brown parts.

***Abdomen*.** Oval, widest at posterior margin of tergite 2, with tergite 3 approximately parallel-sided; blackish brown, except pale brown on posterior margin of tergite 4; tergite 1 white pilose; tergite 2 short black pilose except longer yellow pilosity along anterior and lateral margins, tergite 3 short black pilose except golden yellow pilose along lateral and posterior margins, tergite 4 entirely thick golden pile. Sternites dark brown; white pilose, except sternite 1 bare. Genitalia as in Fig. 280.

**Female.** Unknown.

##### Diagnosis.

Body length: male 7 mm (female unknown). A small species with densely golden pilose tergite 4. Distinguished from similar species by: basoflagellomere longer than scape, mesonotum with anterior and median fascia of golden pile fused into a single wide fascia, which is connected to the posterior fascia by a wide median vitta (Fig. 96). Postpronotum bare.

##### Distribution.

The species is only known from the type locality in Ecuador.

##### Etymology.

The specific epithet *brevis* (Latin) means short, which refers to the sturdy appearance of this species.

#### Peradon chrysopygus

Taxon classificationAnimaliaDipteraSyrphidae

(Giglio-Tos)

95E999F1-BAC0-5D14-91F8-4669F56C4997

Figs 84 , 85 , 141 , 142 , 177–179 , 281

Ubristes chrysopygus Giglio-Tos, 1892: 1. Holotype ♀: Mexico (MRSN). [photographs studied]
Microdon chrysopygus (Giglio-Tos): Thompson et al. 1976: 61.
Peradon chrysopygus (Giglio-Tos): Reemer and Ståhls 2013a; Reemer 2013: 85.

##### Studied type specimens.

Mexico • 1 ♀, holotype of *Ubristeschrysopyga* Giglio-Tos; Orizaba; MRSN. Label 1: “836.”; label 2 (green): “Orizaba”; label 3: “*Ubristes chrysopyga* / Giglio-Tos”. Only photographs of the holotype were studied.

##### Additional specimens.

Belize • 1 ♀; Mtn. Pine Ridge; 14–15 Jan. 1991; MZH.

Costa Rica • 1 ♀; Guanacaste, 3 m SE R. Naranjo; 20–29 Nov. 1991; F.D. Parker leg.; LACM • 1 ♂ 3 ♀; same as previous except date Apr. 1992 • 2 ♀; same as previous except date 1–15 Apr. 1992 • 1 ♀; same as previous except date 16–20 Apr. 1992 • 1 ♂ 2 ♀; same as previous except date 20–30 Apr. 1992 • 2 ♂ 4 ♀; same as previous except date May 1992 • 1 ♀; same as previous except date 11 May 1992 • 1 ♀; same as previous except date 16–31 May 1992 • 1 ♀; same as previous except date 24–31 May 1992 • 1 ♀; same as previous except date 1–5 Jun. 1992 • 1 ♂ 1 ♀; same as previous except date 1–15 Jun. 1992 • 1 ♂; same as previous except date 21–30 Sep. 1992 • 1 ♀; same as previous except date 1–9 Oct. 1992 • 1 ♀; same as previous except date 1–10 Oct. 1992 • 1 ♀; same as previous except date 15–30 Apr. 1993 • 1 ♂ 1 ♀; same as previous except date May 1993 • 2 ♀; same as previous except date 12–14 May 1993 • 1 ♀; same as previous except date 17 May 1993 • 1 ♂ 1 ♀; same as previous except date 1–15 Jun. 1993 • 1 ♀; same as previous except date 10–14 Jun. 1993 • 1 ♂; same as previous except date 18–23 Jun. 1993 • 1 ♂; same as previous except date 22 Jun. 1993 • 1 ♀; same as previous except date 13–31 Jul. 1993 • 1 ♀; same as previous except date 29–31 Jul. 1993 • 3 ♀; same as previous except date 14–20 Aug. 1993 • 2 ♀; same as previous except date 1–14 Sep. 1993 • 1 ♀; same as previous except date 7 Sep. 1993 • 1 ♂; Alajuela, 20 km S Upala; 1–10 May 1990; F.D. Parker leg.; LACM • 1 ♀; same as previous except date 11–15 May 1990 • 1 ♀; same as previous except data 17 Sep. 1990 • 1 ♀; same as previous except date 11–21 Sep. 1991 • 1 ♀; same as previous except date 1–10 Oct. 1992 • 1 ♂; Puntarenas, Cordillera de Tilarán, Monteverde; 17 Aug. 2010; M. Hauser leg.; RMNH (genitalia in fig. 281 drawn after this specimen).

El Salvador • 1 ♂ 1 ♀; Los Chorros National Park; 13 Jul. 1961; M.E. Irwin leg.; CNC.

Mexico • 1 ♀; Oaxaca, El Camaron; 9.VI.1987; T. Taylor leg.; LACM • 1 ♀; Veracruz 24, 1 mi E Jaltipan; 2 Sep. 1972; leg. Byers & Thornhill; SEMC • 1 ♂; Chiapas, Montebello Nat. Pk.; 1 Jun.1969; J.W. Boyes leg.; CNC.

##### Diagnosis.

Body length male 8–10 mm, female 10–12 mm. *Peradonchrysopygus* belongs to the *flavofascium* species group. Within this group, it is the only species in which the male has no yellow wing markings, the wing being entirely dusky grey. In the female there is a large yellow part in the wing basally, whereas cells r_2+3_ and r_4+5_ are entirely dark. The postponotum is pilose.

##### Distribution.

Known from Belize, Costa Rica, El Salvador, and Mexico. This is the only known species of the *flavofascium*-group in Central America.

#### Peradon costaricensis

Taxon classificationAnimaliaDipteraSyrphidae

Reemer sp. nov.

5BD2FAB2-6624-593E-81DA-B988BAC1C7EF

http://zoobank.org/9B180C5E-1C71-44B4-B1F1-6277D133C9EE

Figs 3 , 51 , 62 , 64 , 117 , 118 , 180–185 , 268

##### Type material.

***Holotype*.** Costa Rica • 1 ♂, holotype of *Peradoncostaricensis* sp. nov.; Braulio Carillo National Park; 10°10'N, 84°07'W; 500 m a.s.l.; 10 Apr. 1985; H. Goulet-L. Masner leg.; CNC.

Label 1: “COSTA RICA / B. Carrillo N.P. / 10°10'N, 84°07'W / 10.IV.85; 500 m. / H. Goulet-L. Masner”.

***Paratypes*.** Costa Rica • 1 ♂ 1 ♀; National Park Braulio Carillo, Quebrada Gonzales, 30 km NNE San José; 10°09'N, 83°55'W; 7 Apr. 2004; J.-H. Stuke leg.; ZFMK.

##### Description

**(based on holotype). Adult male.** Body size: 13.5 mm.

***Head*.** Face occupying 0.44 of head width in frontal view; yellow; pale golden yellow pile. Gena black; white pilosity. Lateral oral margin weakly produced; black; white pilosity. Frons and vertex black; pale golden yellow pilosity, except black pilosity at ocellar triangle. Occiput black; pale golden yellow pile dorsally, white pilose ventrally. Eye bare. Antennal fossa approx. as wide as high. Antenna brown. Ratio scape:basoflagellomere approximately 1:1.6. Basoflagellomere parallel-sided with rounded apex. Arista slender, ca. 3/4 of length of basoflagellomere.

***Thorax*.** Mesoscutum dull black; short black pilosity, except for narrow uninterrupted fasciae of golden yellow pile along anterior and posterior margins and transverse suture. Postpronotum brown; golden yellow pile. Postalar callus blackish brown; golden yellow pilosity. Scutellum blackish brown with faint metallic shine, posterior margin yellow; white pilosity; with two apical calcars of ca. 1/4 of length of scutellum, with mutual distance approximately equal to length of scutellum. Pleura brown. Anterior and posterior part of anepisternum separated by deep sulcus; golden yellow pile anteriorly and posteriorly, with wide bare area in between. Anepimeron entirely pale golden yellow pile. Katepisternum white pilose dorsally, bare ventrally. Other pleura bare (except for microtrichiae). Calypter pale yellowish grey, halter yellow.

***Wing*.** Hyaline, slightly brownish in anterior cells; veins around cell br and vena spuria yellow. Wing microtrichose, except bare on posterobasal 1/2 of cell br, basal 3/4 of cell bm (but with for narrow median strip of microtrichiae over entire length of cell), and most of alula (only microtrichose along margins).

***Legs*.** Pale brown; yellow pilosity, except coxae silvery white pilose.

***Abdomen*.** Elongate, widest at posterior 1/2 of tergite 2, tergite 3 slightly tapering distally. Tergites dark brown, except tergite 2 yellowish brown along lateral margins. Tergite 2 with relatively long golden pilosity, except for long white pile anterolaterally. Tergite 3 with very short dark golden yellow pile, except for longer appressed silvery white pile along lateral margins. Tergite 4 with very short blackish pilosity, although under certain angles the pile may seem to have a golden sheen. Sternites brown; yellow pile, except sternite 1 bare. Genitalia as in Fig. 268.

**Female.** As male, except for the following differences: body length 14 mm. Face dark brown. Fascia of golden pile along transverse suture narrowly interrupted medially. Wing yellow on anterior half. Tergite 3 short black pile, except for longer whitish pile laterally. Tergite 5 short black pile.

##### Diagnosis.

Body length: male 12.5–13.5 mm, female 14 mm. Pale specimens are readily recognizable by the colour pattern of the abdomen: entirely dark brown except for yellowish lateral margins of tergite 2 (Fig. 67). Dark specimens differ from the black morph of *P.bidens* by the partly bare wing cell bm, and the uninterrupted fascia of golden pile along the transverse suture (widely interrupted in *P.bidens*). Dark specimens also resemble *P.sciarus* but differ by the presence of a fascia of golden pile along the transverse suture (absent in *P.sciarus*).

##### Notes.

The male paratype is considerably darker in colouration than the holotype and the female paratype. All wing veins are dark and the lateral margins of tergite 2 are only slightly paler than the rest of this tergite. In other characters, however, the specimens are more or less identical.

##### Distribution.

The species is only known from Costa Rica.

##### Etymology.

The specific epithet is an adjective referring to Costa Rica, the country of origin of the type specimens.

#### Peradon diaphanus

Taxon classificationAnimaliaDipteraSyrphidae

(Sack)

5DFD13DF-BF2E-57B4-82BD-2FD7FB055509

Figs 32 , 109 , 186

Microdon diaphanus Sack, 1921: 146. Holotype ♂: Paraguay, St. Trinidad (lost); Thompson et al. 1976: 64.
Peradon diaphanus (Sack): Reemer and Ståhls 2013a: 146.

##### Studied type specimens.

Paraguay • 1 ♀, neotype of *Microdondiaphanus* Sack (new designation, see notes); Encarnacion; 15 Jun. 1927; Shannon & Del Ponte leg.; USNM.

Label 1: “ex ant colony / in arboreal / bromelia”; label 2: “Encarnacion / Paraguay 15.6.27 / Shannon & Del Ponte””; label 3: “USNMENT / [barcode] / 01371103”. Coll USNM. With empty puparium mounted on same pin.

##### Additional specimens.

Brazil • 1 ♀; Jundiahy; 13 Aug. 1899; NHMUK.

Paraguay • 2 ♀; same label data as neotype; USNM.

##### Diagnosis.

Body length: male 16 mm (based on Sack 1921), female 16.5–18.5. The constricted abdomen, absence of a triangle of golden pile on the mesoscutum, and partly dark wings place *P.diaphanus* in a group with *P.elongatus*, *P.hermetia* and *P.hermetoides*. *Peradondiaphanus* differs from the other three species by tergite 2 being longer than wide, and also by the rufous golden pilose mesoscutum.

##### Notes.

According to Pape and Thompson (2013), Reemer and Ståhls (2013a) and Thompson et al. (1976), the type of *Microdondiaphanus* is deposited in the DEI (Müncheberg). However, attempts to find it in that collection failed (pers. comm. F. Menzel). The DEI is part of the Senckenberg research institute, which also holds entomological collections in Dresden (SNSD) and Frankfurt (SMF). Enquiries at these institutions did not result in finding the type either. As a large part of the collection of P. Sack was destroyed during World War II (Franz 1967), it seems probable that this has also happened to the type of *M.diaphanus*. In order to ensure the stability of this taxon, a neotype designation is deemed desirable. One female from Brazil (coll. NHMUK) and three females from Paraguay (coll. USNM) agree well with the original description and the figure in Sack (1921). As the species was originally described from Paraguay, one of the Paraguayan females is here designated as neotype.

The three females from Paraguay are mounted together with empty puparia and carry labels stating “ex ant colony in arboreal bromelia”. This is the first known record of an association of a *Peradon* species with ants. Unfortunately, the ants remain unidentified.

##### Distribution.

Known from the Brazilian state São Paulo and from Paraguay.

#### Peradon elongatus

Taxon classificationAnimaliaDipteraSyrphidae

(Hull)

AC9C638A-5197-5F0D-977B-05C5798C07FF

Figs 28 , 29 , 33 , 107 , 108 , 191–193 , 187–193 , 263

Microdon elongatus Hull, 1943: 706. Holotype ♂: Brazil, Pará (NHMUK) [examined].
Argentinomyia elongata (Hull): Thompson et al. 1976: 57.
Peradon elongatus (Hull): Reemer and Ståhls 2013a: 145.

##### Studied type specimens.

Brazil • 1 ♂, holotype of *Microdonelongatus* Hull; Pará, Santarem; NHMUK. Label 1 (small, round, red-bordered): “Holo- / type”; label 2: “Braz. / Santarem”; label 3 (red): “Holotype / Microdon /elongata / Hull”. Coll. NHMUK. • 1 ♀; Villa nova; NHMUK. Label 1 (small, round, yellow-bordered): “Para- / type”; label 2: “Villa / nova”; label 3 (yellow): “Paratype / Microdon / elongata / Hull”.

##### Additional specimens.

Brazil • 1 ♂; Amazonas, Novo Aripuanã, Malaise Igarapé, “Floresta úmida”; 05°15'53"S, 60°07'08"W; Sep. 2004; Henriques, Silva & Pena leg.; INPA (“INPA-DT / 0000080”) • 1 ♀; Amazonas, Parque Nacional Jaú, Arm. Malaise, Campinarana baixa; 8–16 Apr. 2001; 01.5427 S, 61.3510 W; Henriques & Vidal leg.; INPA (“INPA-DIP / 001569”).

##### Diagnosis.

Body length: male 10.5–11 mm, female 11–12 mm. The basally constricted abdomen, absence of a golden pilose triangle on the mesoscutum, and partially infuscated wings place this species in a group together with *P.diaphanus*, *P.hermetia* and *P.hermetoides*. Among these species, *P.elongatus* is the only one with yellow colouration on the wing: in the male, only the vena spuria is yellow, in the female the yellow parts are more extensive.

##### Distribution.

Brazil (Amazonas, Pará).

##### Notes.

The yellow wing colouration is much more extensive on the female than on the male. Similar sexual dimorphism in wing colouration also occurs in other *Peradon* species, e.g., *P.chrysopygus* and *P.flavofascium*. The male from the Brazilian state Amazonas was collected in humid forest along a small stream (“igarapé”). The female from Amazonas was collected in a “campinarana”: a type of vegetation occurring in flat, sandy soils prone to waterlogging, usually dominated by thin trees (pers. comm. G.F.G. Miranda).

#### Peradon fenestratus

Taxon classificationAnimaliaDipteraSyrphidae

(Hull)

AF0C7E5E-E9EE-53F1-A377-2F5749592ABF

Figs 16 , 99 , 194–198 , 256

Microdon fenestratus Hull, 1943: 712. Holotype ♂: Amazon (NHMUK) [examined]; Thompson et al. 1976.
Peradon fenestratus (Hull): Reemer and Ståhls 2013a: 146.

##### Studied type specimens.

Amazon Region • 1 ♂, holotype of *Microdonfenestratus* Hull; NHMUK.

Label 1 (small, round, red-bordered): “Holo- / type”; label 2: “Amazon / 66 53”; label 3 (red): “Holotype / Microdon / fenestratus / Hull”.

##### Additional specimens.

Brazil • Amazonas: 3 ♀; Barcelos, Rio Demeni Pirico; 01°19'30"S, 62°47'21"W; Aug. 2008; A. Silva & R. Machado leg.; INPA • 1 ♀; Barcelos, Rio Demeni Alubiá; 00°16'07"S, 62°44'45"W; Aug. 2008; A. Silva & R. Machado leg.; INPA • 1 ♀; Barcelos, Serrinha; 00°25'05"N, 63°23'05"W; Jul. – Aug. 2007; A.S. Filho & T. Krolow leg.; INPA. French Guiana • 1 ♂; St-Laurent du Maroni; 1909; E. Le Moult leg.; MNHN.

##### Diagnosis.

Body length: male 17 mm, female 17–19 mm. The triangle of golden pile on the mesoscutum place this species in a group with *P.aureoscutus*, *P.aureus*, *P.trilinea* and *P.trivittatus*. *Peradonfenestratus* differs from all four of those by the erect (instead of appressed) pile on the scutellum (Fig. 16).

##### Notes.

Label information of the studied Brazilian specimens in the INPA collection states that these were collected in “terra firme” (non-flooded) forest and at a small stream (“igarapé”).

##### Distribution.

Known from the Brazilian Amazon region and from French Guiana.

#### Peradon flavipennis

Taxon classificationAnimaliaDipteraSyrphidae

(Curran)

1CEB01B1-BA50-525B-BF24-D14B04CF5E68

Figs 41 , 125 , 199 , 200

Microdon flavipennis Curran, 1925: 342. Holotype ♀: Guyana, Bartica (MCZ) [examined]; Thompson et al. 1976: 64.
Peradon flavipennis (Curran): Reemer and Ståhls 2013a: 145.

##### Studied type specimens.

Guyana • 1 ♀, holotype of *Microdonflavipennis* Curran; Bartica; 5 Apr. 1901; C.W. Johnson leg.; MCZ. Label 1: “Bartica, BG / IV-5-1901”; label 2: “Collection / C.W. Johnson”; label 3: “M. / flavipennis Curran / Det. / C.H. Curran”; label 4 (red): “TYPE / Microdon / flavipennis / Curran”; label 5 (red): “Type / 7657”; label 6: “Jan.-July 2003 / MCZ Image / database”; label 7: “MCZ-ENT / 00007657”.

##### Diagnosis.

Body length: female 17 mm. The alula is largely bare and cell br is largely bare posteriad of the vena spuria. These characters separate *P.flavipennis* from *P.niger* and *P.pompiloides*, two other species with a contrasting wing pattern. However, *P.flavipennis* is most similar to morph SUR-17b of *P.normalis*, from which it differs by the more extensive yellow in the wing and the different position of the dark cloud in the wing, as described in the key. Theoretically, *P.flavipennis* might be yet another colour form of *P.normalis*. However, with only the type specimen available and DNA data lacking, it seems better to be conservative with regard to the specific status of *P.flavipennis*.

##### Distribution.

Only known from the type specimen from Guyana.

#### Peradon flavofascium

Taxon classificationAnimaliaDipteraSyrphidae

(Curran)

13D5CC08-85CB-572C-AF0F-7F30CAD61459

Figs 12 , 74 , 75 , 86 , 88 , 90 , 92 , 138 , 201–205 , 277

Microdon flavofascium Curran, 1925: 346. Holotype ♂: Brazil, Minas Gerais, Lassance (CU) [examined].
Not Microdonflavofascium Curran of Van Doesburg 1966: 80; see P.surinamensis sp. nov.
Not Peradonflavofascium (Curran) of Reemer 2014: 43; see P.surinamensis sp. nov.

##### Studied type specimens.

Brazil • 1 ♂, holotype of *Microdonflavofascium* Curran; Minas Garais, Lassance; 9–19 Nov. 1919; Cornell Univ. Expedition leg.; CU. Label 1: “Lassance, Min- / as Ger’s Brazil / 9–19 Nov. 1919”; label 2: “Cornell Univ. Ex- / pedition. Lot 569”; label 3 (red): “TYPE / *Microdon* / *flavofasciatum* [sic] / Curran / No.”; label 4 (pink): “HOLOTYPE / Cornell U. / No. 1737”; label 5: “Microdon / flavofascium / Curran / Det. / C.H. Curran”.

##### Additional specimens.

Brazil • 1 ♂ 1 ♀; Utiariti, Rio Papagaio, Mt; Oct. 1966; Lenko & Pereira leg.; MZUSP • 1 ♀; Utiariti, Rio Papagaio, Mt; Nov. 1966; Lenko & Pereira leg.; MZUSP • 1 ♂; Minas Gerais, Serra Caraça; Nov. 1961; Kloss, Lenko, Martins & Silva leg.; MZUSP • 1 ♀; Cáceres, MT; 9–11 Nov. 1984; C. Elias leg.; UFPR.

##### Diagnosis.

Body length: male 7.5–8.5 mm, female 8.5–9.0 mm. A rather small species of *Peradon* with a yellow macula in the wing, silvery white pile on tergites 4 (and 5 in the female) and a bare postpronotum. These three characters are only shared with *P.surinamensis* sp. nov., from which it differs as follows: alula bare for maximally 30% (only narrowly microtrichose in *P.surinamensis*), male with yellow wing macula extending to cell r_1_ anteriorly, and additional characters stated in the key.

##### Notes.

The genitalia of the male holotype are figured in Fig. 277. The genitalia of additional specimens from the Brazilian state of Minas Gerais (where the holotype is also from) were found to look more similar to those of *P.surinamensis* sp. nov. However, in external characters these specimens are very similar to the type of *P.flavofascium*. Re-examination of the latter type revealed that the appearance of the surstylus strongly depends on the viewing angle: from certain angles, the shape of the surstylus is similar in *P.flavofascium* and *P.surinamensis*. Despite this, the specimens are considered different enough in external characters to consider them as different taxa.

##### Distribution.

Known from the Brazilian states Mato Grosso and Minas Gerais.

#### Peradon hermetia

Taxon classificationAnimaliaDipteraSyrphidae

(Curran)

23517B56-A513-5118-B500-9815C2172F8B

Figs 34 , 105 , 206–209 , 261

Microdon hermetia Curran, 1936: 3. Holotype ♂: Panama (AMNH) [examined]; Thompson et al. 1976: 65.
Peradon hermetia (Curran): Reemer and Ståhls 2013a: 146.

##### Studied type specimens.

Panama • 1 ♂, holotype of *Microdonhermetia* Curran; Barro Colorado Island, Canal Zone; 23 Dec. 1928; C.H. Curran leg; AMNH. Label 1: “Barro Colo Isld. / Canal Zone / XII-23-1928”; label 2: “Collector / C.H. Curran”; label 3 (red): “Microdon / hermetia / Curran. [male sign] / Holotype”.

##### Diagnosis.

Body length: male 16 mm. The constricted abdomen, absence of a triangle of golden pile on the mesoscutum, and partly dark wings place this species in a group with *P.diaphanus*, *P.elongatus*, and *P.hermetoides*. From *P.diaphanus* it differs by tergite 2 being wider than long, from *P.elongatus* by the absence of yellow in the vena spuria, from *P.hermetoides* by the partly hyaline wing cell bm. The male genitalia are figured in Fig. 261.

##### Distribution.

Only known from the type specimen from Panama.

#### Peradon hermetoides

Taxon classificationAnimaliaDipteraSyrphidae

(Curran)

2675422E-878F-5206-ACCC-2EEF78AA00A5

Figs 35 , 106 , 210 , 211 , 262

Microdon hermetoides Curran, 1940: 8. Holotype ♂: Guyana (NHMUK) [examined]; Thompson et al. 1976: 65.
Peradon hermetoides (Curran): Reemer and Ståhls 2013a: 146.

##### Studied type specimens.

Guyana • 1 ♂, holotype of *Microdonhermetoides* Curran; Essequibo River, Moraballi Creek, dark forest; 31 Sept. 1929; Pxf. University Expedition leg.; NHMUK. Label 1 (small, round, red-bordered): “Holo- / type”; label 2: “Dark forest / British Guiana: / Essequibo R., / Moraballi Creek. / 31.IX.1929. / Pxf. Univ. Expedn. / B.M. 1929-485.”; label 3 (red): “Microdon / hermetoides / Curran [male sign] / Holotype”; label 4: “Microdon / hermetoides / Curran / Det. / C.H. Curran”; label 5: “note 392”; label 6: “2508.”.

##### Additional specimens.

French Guiana • 1 ♀; Roura, Kaw Road, PK37 (km 37), Relais Patawa; 04°32'42"N, 52°09'09"W; Nov. 2008; J.A. Cerda leg.; RMNH.

##### Diagnosis.

Body length: male 13.5 mm, female 12.5 mm. The constricted abdomen, absence of a triangle of golden pile on the mesoscutum, and partly dark wings place this species in a group with *P.diaphanus*, *P.elongatus*, and *P.hermetia*. From *P.diaphanus* it differs by tergite 2 being approx. as wide as long, from *P.elongatus* by the absence of yellow in the vena spuria, from *P.hermetia* by the entirely infuscated wing cell bm. The male genitalia are figured in Fig. 262.

##### Distribution.

Known from Guyana and French Guiana.

#### Peradon luridescens

Taxon classificationAnimaliaDipteraSyrphidae

(Walker)

A1CB79AC-A287-58CC-AD70-1DE42813B574

Figs 69 , 132 , 133 , 212–215 , 274

Ceratophya luridescens Walker, 1857: 151. Holotype ♀: Amazon (NHMUK). [examined]
Microdon luridescens (Walker): Thompson et al. 1976: 65.
Peradon luridescens (Walker): Reemer and Ståhls 2013a: 146; Reemer 2014: 44.

##### Studied type specimens.

Amazon Region • 1 ♀, holotype of *Ceratophyaluridescens* Walker; NHMUK. Label 1 (round, red-bordered): “Holo- / type”; label 2 (round, green-bordered): “Type”; label 3: “Amaz”; label 4: “luridescens Wlkr”; label 5: “Ceratophya / luridescens. / Wlk.”.

##### Additional specimens.

Brazil • 1 ♂; Acre, 15 km SE Rio Branco, Emprapa; 10°01'S, 67°41'W; 9 Jul.2008; G. Melo leg.; UFPR • 1 ♂; Roraima, Rio Uraricoera, Ilha de Maraca; 2–18 May 1987; Rafael leg.; INPA • 1 ♂; Amazonas, R. Campina; 22 Jan. 1987; F.J.A. Peralta leg.; INPA • 1 ♀; Rondonia, 62 km S Ariquemes, Fazenida Rancho Grande; 10.53°S, 62.80°W; 19–29 Sep. 1996; B. Harris leg.; LACM • 1 ♂; Pará, Canindé, Rio Gurupí; May 1963; B. Malkin leg.; MZUSP • 1 ♀; Amazonas, Manaus; 20 Sep. 2001; J.A. Rafael & J.F. Vidal leg.; INPA • 1 ♀; Amazonas, Resex Unini, Rio Unini, Lg. Galomanha, Terra Firme; 13–28 Jul. 2004; M.L. Oliveira, L. Aquino & A. Silva-Filho leg.; INPA.

Peru • 1 ♂; Madre de Dios, Rio Tambopata Reserve, 30 air km SW of Puerto Maldonado; 1–26 Nov. 1982; E.S. Ross leg.; CAS • 1 ♂; Madre de Dios, Rio Tambopata, Sachavacayoc centre, main trail, mal. trap; 12°51'46.4"S, 69°21'46.6"W; 16–24 Mar. 2011; J.T. Smit; JTS. SURINAME • 1 ♀; Brownsberg; 14 Sep. 1938; D.C. Geijskes leg.; RMNH • 1 ♂; Lely; 30 Oct. 1979; G.F. Mees leg.; RMNH • 2 ♂; Nason; 19 Mar. 2006; M. Reemer leg.; RMNH • 2 ♂; Nassau Mts.; 23 Mar. 2006; M. Reemer leg.; MZH & RMNH • 3 ♂; Nassau Mts.; 24 Mar. 2009; M. Reemer leg.; RMNH.

##### Diagnosis.

Body length: male 13–15 mm, female 16 mm. A large species with elongate, unconstricted abdomen. Tergite 2 has a pair of large yellowish maculae, while both tergites 3 and 4 are entirely dark brown (at least tergite 3 is yellowish brown in the otherwise very similar *P.angustiventris*). The wings are yellow anterobasally, the fascia of golden pile along the mesonotal transverse suture is complete (not interrupted medially), and the face is entirely yellow. Male genitalia as in Fig. 274.

##### Notes.

This species is closely related to *P.angustiventris* and the (colour) character used here to distinguish between them may not be sufficient. The male genitalia are very similar as well. For further notes see *P.angustiventris*. The colour of the scutellum seems to divide the available specimens of *P.luridescens* into two more or less discrete groups: in some the scutellum is entirely yellow, while in other specimens it is black with a greenish metallic hue, leaving only the margins narrowly yellow. In males with a dark scutellum the wings tend to be more extensively yellow towards the apex than in males with a yellow scutellum. In all studied females, the yellow colouration of the wing extends all the way to the wing apex, except in one specimen (Brazil, Manaus, 5–8.II.2005), in which it does not reach further than crossvein rm.

##### Distribution.

Known from the Brazilian states Acre, Amazonas, Pará, Rondonia, Roraima, from Amazonian parts of Peru, and from Suriname.

#### Peradon niger

Taxon classificationAnimaliaDipteraSyrphidae

(Williston)

9FA5C9DD-4C78-5167-AD11-E48DC2B7B36A

Figs 46 , 123 , 124 , 216 , 217 , 270

Microdon niger Williston, 1891: 4. Holotype ♂: Mexico (NHMUK) [examined]; Thompson et al. 1976: 66.
Microdon manni Shannon, 1923: 80. Holotype ♀: Bolivia (USNM) [examined].
Peradon niger (Williston): Reemer and Ståhls 2013a: 146.

##### Studied type specimens.

Mexico • 1 ♂, holotype of *Microdonniger* Williston; Pancina, Vera Paz., Champion”; 1903; F.D. Godman & O. Salvin leg.; NHMUK. Label 1 (small, round, red-bordered): “Holo- / type”; label 2: “Pancina, / Vera Paz. / Champion.”; label 3: “Sp. figured.”; label 4: “Microdon [male sign] / niger, Will.”; label 5: “Biol. Centr. Amer. / Dipt. - Syrphidae. / F.D. Godman, / O. Salvin. / 1903-51.”.

Bolivia • 1 ♀, holotype of *Microdonmanni* Shannon; USNM. Label 1: “Ivon Beni / Mann. Bol.”; label 2: “W.M. Mann / collector”; label 3 (red): “Type No. / 25951 / U.S.N.M.”; label 4: “Microdon / manni / Shannon”; label 5: “= niger Will. / C.T.G.”; label 6 (barcode): “USMM ENT 00250236”.

##### Additional specimens.

Mexico • 1 ♂; Chiapas, 6.0 km SW Ocosingo; 22 Sep. 1992; M. Wood leg.; CNC.

Peru • 1 ♀; Madre de Dios, Rio Tambopata Reserve, 30 air km SW Puerto Maldonado; 1–26 Nov. 1982; E.S. Ross leg.; CAS.

##### Diagnosis.

Body length: male 14.5–15 mm, female 15.5–16 mm. This is a species with a black body and blackish wings with a large whitish apical wing mark. From similarly coloured species (*P.bispina*, *P.normalis*, *P.pompiloides*) this species differs by the entirely microtrichose alula.

##### Notes.

The two specimens from Mexico (including the type of *P.niger*) are males, whereas the two specimens from Bolivia (i.e., the type of *P.manni*) and Peru are both females. Apart from usual sexual dimorphism, no morphological differences could be found. This supports the synonymization of *Microdonmanni* Shannon with *M.niger* Williston by Thompson et al. (1976). However, the type localities of these taxa (Bolivia and Mexico, respectively) are far apart, and the types are of opposite sexes. Support for this synonymy would be stronger if males and females of both areas could be compared with each other.

##### Distribution.

Known from southern Mexico and Amazonian parts of Bolivia and Peru.

#### Peradon normalis

Taxon classificationAnimaliaDipteraSyrphidae

(Curran)

3B4BA2A5-1D0E-5328-958E-0201F0188814

Figs 42 , 43 , 53 , 54 , 57–59 , 66 , 119–122 , 218–221 , 269

Microdon normalis Curran, 1925: 343. Holotype ♀: Guyana (AMNH) [examined]; Thompson et al. 1976: 66.
Not Microdonnormalis Curran of Van Doesburg 1962: 13, 1966: 83.
Peradon normalis (Curran): Reemer and Ståhls 2013a: 146.
Peradon SUR-17a of Reemer 2014: 47.
Peradon SUR-17b of Reemer 2014: 47.

##### Studied type specimens.

Guyana •1 ♀, holotype of *Microdonnormalis* Curran; Demara River, West Bank; 9 Feb. 1923; AMNH. Label 1: “W. Bank, Dem. R. / 9-ii-1923”; label 2 (red): “TYPE / Microdon / normalis / Curran”; label 3: “Microdon / normalis / Det. C.H. Curran”. Coll. AMNH. Type locality according to Curran (1925): West Bank Demarara River.

##### Additional specimens of typical morph.

Brazil • 1 ♀; Pará, Guama; 8 May 1956; E. Lobato leg.; MZUSP.

French Guiana • 1 ♀; Roura, Kaw Road, PK37 (km 37), Relais Patawa; 04°32'43"N, 52°09'09"W; Nov. 2008; J.A. Cerda leg.; RMNH.

Suriname • 1 ♀; Brownsberg; 04°56'45"N, 55°10'59"W; 2 Apr. 2006; M. Reemer leg.; RMNH [previously published as *Peradon* SUR-17a by Reemer 2014].

##### Additional specimens of red morph.

Brazil • 1 ♀; Pará; Baker leg.; LACM.

##### Additional specimens of SUR-17b morph.

Brazil • 1 ♀; Amazonas, Cepiac, Manaus; 3 Apr. 1977; INPA.

Suriname • 1 ♀; Brownsberg; 04°56'45"N, 55°10'59"W; 2 Apr. 2006; M. Reemer leg.; RMNH [previously published as *Peradon* SUR-17b by Reemer 2014].

##### Additional specimens of P.cf.normalis.

Brazil • 1 ♂; Rondonia, 62 km SE Ariquemes; 8–20 Nov. 1994; W.J. Hanson leg.; LACM.

##### Diagnosis.

Body length: male 13 mm (based on P.cf.normalis), female 11–17 mm. In the concept presented here, *Peradonnormalis* is a very variable species in colouration. In females, three colour morphs are recognized. In the typical morph the abdomen and legs are black and the wings are blackish with a subapical yellow marking (Figs 43, 120). In the red morph the abdomen and legs are red, and the wings are coloured as in the typical morph (Fig. 121). In morph SUR-17b the abdomen and legs are black, and the wings are yellow basally with a blackish subapical marking, almost a photo negative of the typical variation (Figs 42, 122). In all of these variations, the alula is largely bare and cell br is largely bare posteriad of the vena spuria. These characters separate *P.normalis* from the other species with blackish wings and subapical yellow wing markings: *P.bispina*, *P.niger* and *P.pompiloides*. Morph SUR-17b resembles *Peradonflavipennis* in wing colouration, but differs from that species by the more extensive yellow in the wing and the different position of the dark cloud in the wing, as described in the key. Additional characters distinguishing females of *P.normalis* from females of *P.pompiloides* and P.cf.sciarus are the absence of a basomedian patch of greyish pruinescence on tergite 4 (Figs 53, 54), and the presence of greyish pruinescence on the basal 1/3 of tergite 3 (Figs 57–59). These characters are only visible when viewing from a frontal angle. The male is not known with certainty. One male specimen from Brazil (Rondonia) is here preliminarily assigned to P.cf.normalis because of the partly bare alula and wing cell br, and the presence of small, not interconnected yellow marks in the wing apex (Fig. 119). Genitalia as in Fig. 269.

##### Notes.

The typical colour morph and the red morph are considered conspecific because of the identical morphology, including the patterns of the wing microtrichosity. The pattern of greyish pruinescence on tergite 3 is also similar in both colour forms (Figs 57–59). A similar case of colour variation is found in *Peradonbidens*. In the case of *Peradon* SUR-17b of Reemer (2014), the barcode is identical to that of *Peradon* SUR-17a (see paragraph *Barcoderesults*). *Peradon* SUR-17b does not agree in morphology and colouration with any described species, but *P.* SUR-17a corresponds with the type of *P.normalis* in all morphological and colour characters, except that it is smaller (body length 11 instead of 17 mm). The specimen of *Peradon* SUR-17a is identical in morphology and body size to *P.* SUR-17b. Both specimens of *P.* SUR-17a and *P.* SUR-17b were collected at exactly the same locality within five minutes on the same day (Reemer 2014). Combined with the identical barcodes and identical morphology this suggests that the specimens belong to the same species, so both are here assigned to *P.normalis*. Extra support for this conclusion is provided by the similar pattern of greyish pruinescence on tergite 3 (Figs 57–59).

In the only male specimen assigned to *P.normalis*, the yellow wing markings are not interconnected, so they do not form one large subapical macula as is found in the females of the typical variation. However, such sexual dimorphism in which the yellow wing colouration is less extensive in the male is also known from other species of *Peradon*, such as *P.chrysopygus*, *P.flavofascium* and *P.luridescens*. Whether this male specimen really belongs to *P.normalis* can only be resolved based on additional material, which is currently unavailable.

The only known specimen of *Peradonflavipennis* only differs from *P.normalis* in wing colouration. Therefore, it seems possible that *P.flavipennis* is merely a colour form of *P.normalis*. Without any further specimens or DNA data available, however, it seems premature to change the taxonomic status of *P.flavipennis*.

##### Distribution.

Known from the Brazilian states Pará and Rondonia, French Guiana, Peru, and Suriname.

#### Peradon notialus

Taxon classificationAnimaliaDipteraSyrphidae

Reemer sp. nov.

F2794491-101E-5B7D-9320-74FCC5ABC9C6

http://zoobank.org/219DD9AE-6614-4AEA-8D6C-36D4040982E3

Figs 82 , 83 , 94 , 146 , 147 , 222–227 , 279

##### Type material.

***Holotype*.** Argentina • 1 ♂, holotype of *Peradonnotialus* sp. nov.; Prov. Tucumán, N307 betw. Monteros and Tafi del Valle nr. km 16; 27°05.70'S, 65°36.93'W; 560 m a.s.l.; 13 Oct. 2003; S.M. Blank & C. Kutzscher; CSCA. Label 1: “Argentina: Prov. Tucumán, / N307 betw. Monteros and / Tafi del Valle nr km 16 / 27°05.70'S, 65°36.93'W / 560 m alt., 13.10.2003, / S.M. Blank & C. Kutzscher”.

***Paratypes*.** Brazil • 1 ♂, paratype of *Peradonnotialus* sp. nov.; Rio Grande do Sul, 60 km NE de Bagé, Palmas; 30°59'S, 53°37'W; 270 m a.s.l.; 17 Nov. 2007; E. & J. Almeida leg.; UPFR • 1 ♀; same data as previous paratype except leg. D. Parizotto.

##### Description

**(based on holotype). Adult male.** Body size: 6 mm.

***Head*.** Face occupying 0.48 of head width in frontal view; black; white pile. Gena black; white pilosity. Lateral oral margin weakly produced; black; white pilosity. Frons and vertex black; black pile. Occiput black; white pilosity. Eye bare. Antennal fossa approx. as wide as high. Antenna brown. Length ratio of scape:basoflagellomere approximately 1:1. Basoflagellomere parallel-sided with rounded apex. Arista slender, ca. 2/3 of length of basoflagellomere.

***Thorax*.** Mesoscutum dull black; short, pale yellowish pile, except for narrow fascia of silvery white pile across mesoscutum. Postpronotum brown; bare. Postalar callus brown; yellow pilosity. Scutellum black with faint metallic shine; yellowish white pilosity; with two apical calcars of ca. 1/4 of length of scutellum, with mutual distance slightly more than 1/2 length of scutellum. Pleura blackish brown. Anterior and posterior part of anepisternum separated by deep sulcus; white pilosity anteriorly and posteriorly, with wide bare area in between. Anepimeron entirely whitish pilosity. Katepisternum white pile dorsally, with very small patch of pile ventrally. Other pleurae bare (except for microtrichiae). Calypter and halter yellow.

***Wing*.** Hyaline, except cells bc, c, sc, and vena spuria yellow. Wing microtrichose, except cell bc largely bare. Legs: brownish black; white pilose, except tarsi ventrally golden yellow pile.

***Abdomen*.** Elongate, widest at apex of tergite 2; blackish brown, except posterior margin of tergite 4 yellow; tergite 1 white pile; tergite 3 with short black pilosity, except longer golden yellow pilosity along lateral and posterior margins; tergite 4 golden yellow pilose, with pile more dense laterally and medially, very sparse in between. Sternites brown; white pilosity, except sternite 1 bare. Genitalia as in Fig. 279.

**Female** As male, except for following differences. Body length 7.5 mm. Length ratio of scape:basoflagellomere approximately 1:1.1. Yellow wing colouration more extensive, including cell br entirely, most of bm, a small anterior part of cup, median parts of r_4+5_ and apex of wing. Pilosity of tergite 5 more or less like that of tergite 4 in the male, except there is a narrow median bare vitta.

##### Diagnosis.

Body length: male 6–8.5 mm, female 7.5 mm. This is the only species of *Peradon* in which the basoflagellomere is (slightly) shorter than the scape, and also one of the few with a fully microtrichose alula (this character is only shared with *P.manni* and *P.niger*, which have largely blackish wings and lack the golden pile on tergites 4 and 5).

##### Distribution.

The species is known from Tucumán province (Argentina) and the Brazilian state Rio Grande do Sul.

##### Etymology.

The specific epithet is an adjective derived from the Latin *notialis*, meaning southern, and refers to the distribution of this species in southern parts of South America.

#### Peradon oligonax

Taxon classificationAnimaliaDipteraSyrphidae

(Hull)

030C2603-CB73-5A7A-B087-0C632370EA7C

Figs 1 , 30 , 110 , 111 , 228–231 , 264

Microdon oligonax Hull, 1944: 35. Holotype ♀: Brazil (CU) [examined]; Thompson et al. 1976: 66 [type locality erroneously stated as Ecuador].
Peradon oligonax (Hull): Reemer and Ståhls 2013a: 146; Marín-Armijos et al. 2017: 185.

##### Studied type specimens.

Brazil • 1 ♀, holotype of *Microdonoligonax* Hull; Pto. America, R. Putumayo; 30 Aug. –2 Sep. 1920; Cornell University Expedition leg.; CU. Label 1: “Pto. America, R. Putumayo BRAZIL, Aug. 30 Sep. 2 ‘20”; label 2: “Cornell Univ. Expedition. Lot 569”; label 3 (red): “HOLOTYPE Cornell U. No. 2197”; label 4 (red): “Holotype oligonax Hull”; label 5:”Holotype Microdon oligonax Hull”.

##### Additional specimens.

Brazil • 1 ♂; Amazonas, Tabalinga; 11–14 Jul. 1991; Socorro & Vidal leg.; INPA • 1 ♂; Rondonia, 62 km SE Ariquemes; 7–18 Nov. 1995; W.J. Hanson; LACM.

Bolivia • 1 ♂; Songo; RMNH • 1 ♂; La Paz Prov., Mapiri Arroyo Tuhiri; 15°17'26"S, 68°15'46"W; 508 m a.s.l.; 13 Apr. 2004; M. Hauser leg.; CSCA.

Brazil • 1 ♂; Amazon “66.53”; NHMUK.

Colombia • 1 ♂; Vaupes, Mirafiores; 31 Jan. –5 Feb.1972; M. Cooper leg.; NHMUK.

Ecuador • 1 ♂; Pompeya, Napo R., Pastaza; 14–22 May 1965; L. Pena leg.; CNC •1 ♂; Napo, Tena; 9–14 Dec. 1971; M. Cooper leg.; NHMUK.

Peru • 1 ♀; Tingo Maria; 670 m a.s.l.; Weyrauch leg.; CNC • 1 ♂; Previsto; 8 Jun. 1965; J. Schunke leg.; NHMUK • 3 ♂; Previsto; 25 Jun. 1965; J. Schunke leg.; NHMUK • 1 ♂; Previsto; 26 Jun. 1965; J. Schunke leg.; NHMUK • 1 ♀; SAM, around San Roque de Cumbaza; 6°23'4.96"S, 76°25'53.47"W; 15–31 Jan. 2015; T. Faasen leg.; RMNH • 1 ♀; Tingo Maria; 670 m a.s.l.; Weyrauch leg.; CNC.

##### Redescription

**(based on holotype). Adult female.** Body size: 16 mm.

***Head*.** Face occupying slightly more than 1/3 of head width in frontal view; yellow; brown pile, except with white pilosity ventrolaterally. Gena brown; with white pilosity. Lateral oral margin strongly produced, brown. Frons black, with black pilosity. Vertex brown; with pale pilosity. Occiput black; with pale pruinescence; black pilosity dorsally, pale pilosity ventrally. Eye bare. Antennal fossa approx. as wide as high. Antenna dark brown, except basoflagellomere black. Antennal ratio approximately 4:1:6. Basoflagellomere parallel-sided with rounded apex. Arista slender, approx. 3/4 of length of basoflagellomere.

***Thorax*.** Mesoscutum dull black; short, appressed black pile. Postpronotum and postalar callus pale brown; mixed black and yellow pilose. Scutellum brown, yellow along posterior margin; brown pilose; with two yellow apical calcars of approx. 1/4 of length of scutellum, with mutual distance ca. four times their length. Pleura brown. Anterior and posterior part of anepisternum separated by deep sulcus; black pilose anteriorly, pale pilose posteriorly, with wide bare area in between. Anepimeron entirely pale pile. Katepisternum white pilose dorsally, bare ventrally. Katatergum and anatergum microtrichose. Other pleurae bare. Calypter and halter yellow.

***Wing*.** Hyaline, tinged yellow on anterior half and all veins yellow; microtrichose, except alula 90% bare leaving only margins microtrichose.

***Legs*.** Fore and mid legs (including coxae and trochanters) yellowish brown, yellow pilose. Hind coxa, trochanter and femur brown, yellow to white pilose. [Hind tibia and tarsus missing in type specimen

***Abdomen*.** Elongate, slightly constricted at segment 2, approx. as wide as thorax, with widest point at anterior part of tergite 4. Sternite 1 brown; bare. Sternite 2 yellow; yellow pile. Sternite 3 yellowish brown; yellow pilose. Sternites 4 and 5 dark brown; dark pilose.

**Male** (based on additional specimens). As female, except for following differences. Body size 14–15 mm. Antennal ratio approximately 4:1:5. Scutellar calcars shorter and less far apart. Abdomen more slender.

##### Diagnosis.

Body length: male 13–16 mm, female 16 mm. Based on the somewhat constricted abdomen, this species is here placed in the *trivittatus* species group. From all other species in this group, *P.oligonax* differs by the presence of a (medially interrupted) fascia of golden pile along the mesonotal transverse suture (similar to several species of the *bidens* species group).

##### Notes.

Colouration varies from reddish brown to blackish. The pale markings on tergite 2 are often fused into one large macula, but sometimes there is a narrow dark median line dividing them. In the specimen labelled ‘Amazon / 66.53’ (coll. NHMUK) the side margins of tergite 2 are slightly more convex in dorsal view than in the other specimens, in which tergite 2 is more parallel-sided.

Thompson et al. (1976) state Ecuador as type locality, but this seems to be incorrect. Both the label of the holotype and the description of Hull (1944) only mention Brazil (Rio Putumayo) as type locality. This is also discussed by Marín-Armijos et al. (2017).

In the key to the species, *Peradonoligonax* is included in the *trivittatus* species group because of its constricted abdomen. However, other characters suggest it may be more closely related to species of the *bidens* group. For instance, the fascia of golden pile along the mesonotal transverse suture is also found in several species of the *bidens* group, and there also are similarities in the male genitalia. Unfortunately, attempts to obtain a DNA barcode of this species failed.

##### Distribution.

Bolivia, Brazil (Amazon region), Colombia, Ecuador, Peru. Alt. 400–860 m.

#### Peradon palpator

Taxon classificationAnimaliaDipteraSyrphidae

Reemer sp. nov.

F1A509C1-3E9B-52E8-8451-3CF4009D5609

http://zoobank.org/24C26642-6D81-4EF6-834C-EB8B18CE887F

**Figures 9–12.**
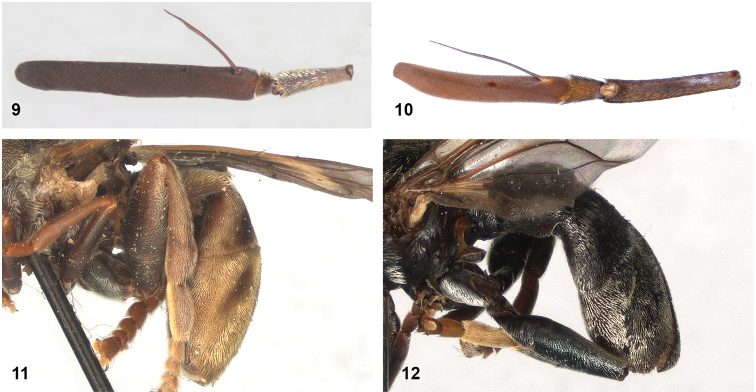
**9, 10** Antennal length ratios: **9***Peradonpalpator* male, basoflagellomere more than twice as long as scape **10***P.bidens* male, basoflagellomere less than twice as long as scape **11, 12** Tergites in lateral view: **11** with golden pilosity (*Peradonaurigaster* male) **12** with silvery white pilosity (*P.flavofascium* male).

Figs 9 , 136 , 137 , 232 , 234 , 276

##### Type material.

***Holotype*.** Argentina • 1 ♂, holotype of *Peradonpalpator* sp. nov.; Tucumán, Horco Molle, ca. 12 km W of Tucumán; 700 m a.s.l.; 17 Mar. 1974; C.R. Vardy leg.; NHMUK. Label 1: “Argentina: Tuc. / Horco Molle ca. 12 km. / W. of Tucuman. 700 m. / Malaise trap / 17.iii.1974. C.R. Vardy / B.M.1974-204”.

***Paratypes*.** Argentina • 1 ♂, paratype of *Peradonpalpator*; Tucumán, Horco Molle, ca. 12 km W of Tucumán; 700 m a.s.l.; 17 Mar. 1974; C.R. Vardy leg.; NHMUK • 5 ♂, paratypes of *Peradonpalpator*; Tucumán, Horco Molle, c. 12 km W of Tucumán; 700 m a.s.l.; 18–21 Mar. 1974; C.R. Vardy leg.; NHMUK • 1 ♂, paratype of *Peradonpalpator* sp. nov.; Tucumán, Horco Molle, ca. 12 km W of Tucumán; 700 m a.s.l.; 22–24 Mar. 1974; C.R. Vardy leg.; NHMUK • 1 ♀, paratype of *Peradonpalpator* sp. nov.; Catamarca Co., Trampasacha, 8 km W of Chumbicha, 650 m; 28°49.97'S, 66°18.29'W; 25 Oct.–12 Nov. 2003; F.D. Parker & M.E. Irwin leg.; LACM.

##### Additional specimens.

Argentina • 1 ♂; Salta Rosario de Lerma; Dec. 1982; Fritz leg.; CAS • 3 ♂; Salta Rosario de Lerma, INESALT yard, Malaise; 16–28 Feb. 1992; S.A. Marshall leg.; DEBU • 1 ♂; Salta Rosario de Lerma, pasture edge; 1 Mar. 1992; S.A. Marshall leg.; DEBU • 1 ♂; Tucuman Prov., 25 km S Tafi del Valle; 1320 m a.s.l.; 27°01.04'S, 65°39.33'W.

Brazil • 1 ♂; Goias, R Saia Velha, 30 km S Brasilia, on Brazilia-B.H. highway; 1 Oct. 1974; L. Knutson leg.; USNM • 1 ♀; D.F. Brasilia, L. Paranoa; 4–5 Oct. 1974; L. Knutson leg.; USNM.

##### Description

**(based on holotype). Adult male.** Body size: 9 mm.

***Head*.** Face occupying approximately 1/2 of head width in frontal view; black; with pale golden pilosity. Gena black; with white pilosity. Lateral oral margin weakly produced; black with white pilosity. Frons black and vertex black, with pale golden pilosity. Occiput black with whitish pilosity. Eye bare. Antennal fossa slightly wider than high. Antenna black, except scape brown. Ratio scape:basoflagellomere approximately 1:2.7. Basoflagellomere parallel-sided with rounded apex. Arista slender, ca. 1/2 of length of basoflagellomere.

***Thorax*.** Mesoscutum dull black; short, appressed black pilose, except for fasciae of pale golden pile along anterior margin, transverse suture (medially interrupted), and posterior margin. Postpronotum and postalar callus brown; pale pile. Scutellum black; pale golden pilose; with two apical calcars of ca. 1/4 of length of scutellum, with mutual distance approx. the same as length of scutellum. Pleura black. Anterior and posterior part of anepisternum separated by deep sulcus; golden pilose anteriorly, whitish pilose posteriorly, with wide bare area in between. Anepimeron entirely whitish pilose. Katepisternum white pile dorsally, bare ventrally. Katatergum and anatergum dark microtrichose. Other pleurae bare. Calypter whitish. Halter dark yellow.

***Wing*.** Hyaline, tinged yellow in costal and subcostal cells, and with yellow stripe over posterior half of br, ranging along vena spuria to apex of r_4+5_, posteriorly extending into dm. Wing microtrichose, except bare on posterobasal 2/5 of br, basal 3/4 of bm, anterobasal 1/4 of cup, and most of alula (only microtrichose along margins).

***Legs*.** Black; silvery white pile, except tarsi ventrally pale golden pilose.

***Abdomen*.** Elongate, widest at apex of tergite 2; black; mostly pale golden pilose but more silvery white laterally, with large triangular patches laterally on tergite 3. Sternites blackish brown; white pilose, except sternite 1 bare.

**Female** (based on 1 paratype from Argentina, Catamarca). As male, except for following differences. Body size: 9.5 mm. Ratio of scape:basoflagellomere apporximately 1:2. Mesoscutum with only very narrow fasciae of pale golden pile. Yellow wing markings more extensive, including basal parts of cells r_1_ and cup. Tergites mostly black pilose, except pale golden pilose on tergite 1, tergite 2 medially, lateral margins of tergites 3 and 4, and posterior triangular parts on tergites 3 and 4.

##### Diagnosis.

Body length: male 7–10 mm, female 9.5 mm. This is the only known *Peradon* species in which the basoflagellomere is more than twice as long as the scape. The body is entirely black and its pale golden and silvery white pilosity is not very conspicuous.

##### Notes.

The fascia of golden pile along the mesonotal transverse suture is either complete or medially interrupted. In the key, this species is included in the *flavofascium* species group, even though it lacks the conspicuous golden or silvery pile on tergite 4 characteristic of this group. Nevertheless, it is hypothesized to be related to this group because of similarities in the male genitalia, especially the shape of the surstylus.

##### Distribution.

The species is known from northwestern Argentina (provinces Catamarca, Salta, and Tucumán), and central Brazil (state of Goiás).

##### Etymology.

The specific epithet *palpator* (Latin: stroker, taster, feeler; noun in apposition) is inspired by the long antennae of this species.

#### Peradon pompiloides

Taxon classificationAnimaliaDipteraSyrphidae

Reemer sp. nov.

E922A463-330B-55AF-9F2A-F024B813C9D8

http://zoobank.org/91FB0D15-9A39-4D57-9FE7-E143A01D997F

Figs 44 , 45 , 55 , 60 , 65 , 126 , 127 , 235 , 237 , 272

Peradon SUR-18 of Reemer 2014: 47.

##### Type material.

***Holotype*.** Ecuador • 1 ♂, holotype of *Peradonpompiloides* sp. nov.; Napo, Jatun Sacha Res., 6 km E Misahualli; 1°4'S, 77°37'W; 450 m a.s.l.; 30 Apr.–8 May 2002; S.A. Marshall leg.; DEBU. Label 1: “ECU [Ecuador]: Napo, Jatun Sacha / Res., 6 km E. Misahualli, 450 / m. 1°4'S, 77°37'W, SOL / trail, 30 Apr–8 May 2002, / S.A. Marshall, / debu00179136”.

***Paratype*.** Brazil • 1 ♀; “Amazon 66 53”; NHMUK.

##### Additional specimens.

Suriname • 1 ♂; “Amer. mer. Surinam”; RMNH.

##### Description

**(based on holotype). Adult male.** Body size: 9.5 mm.

***Head*.** Face occupying 0.43 of head width in frontal view; yellow; with white pilosity. Gena black with white pilosity. Lateral oral margin weakly produced; black with white pilosity. Frons and vertex black, with pale golden yellow pilosity, except black pilosity at ocellar triangle. Occiput black with pale golden yellow pilosity dorsally, white pilosity ventrally. Eye bare. Antennal fossa slightly wider than high. Antenna black, except scape brown basally. Antennal ratio approximately 1:1.4. Basoflagellomere parallel-sided with rounded apex. Arista slender, ca. 2/3 of length of basoflagellomere.

***Thorax*.** Mesoscutum dull black with faint bronze hues on wide areas along all margins; short, appressed black pilose, except for fasciae of more erect golden yellow pile along anterior and posterior margins. Postpronotum pale brown; pale yellow pile. Postalar callus dark brown; black pile. Scutellum dark brown with faint bronze hue; pale yellow pilose anteriorly, black pilose posteriorly; with two apical calcars of approx. 1/4 of length of scutellum, with mutual distance ca. the same as length of scutellum. Pleura blackish brown. Anterior and posterior part of anepisternum separated by deep sulcus; golden yellow and black pile anteriorly, yellow pilose along posterior margin, with wide bare area in between. Anepimeron entirely white pilose. Katepisternum white pilose dorsally, bare ventrally. Katatergum and anatergum dark microtrichose. Other pleurae bare. Calypter grey. Halter yellow.

***Wing*.** Blackish anterobasally and dark grey otherwise, except for yellow subapical macula in parts of cells r_1_, r_2+3_, r_4+5_ and apex of dm. Wing microtrichose, except bare on basal 50% of alula.

***Legs*.** Blackish brown; yellow to white pile, except fore and mid femora black pile anteriorly, and hind femora black pilose posteriorly.

***Abdomen*.** Elongate, widest at apex of tergite 2; black; short black pilose, except longer yellowish to white pilose on following parts: posterior part of tergite 1, anterior margin and anterolateral corners of tergite 2, lateral margins of tergite 3 and most of tergite 4. Sternites blackish brown; black pilose, except sternite 1 bare.

**Female** (based on paratype from Brazil, Amazon region). As male, except for following differences. Body size: 12 mm. Tergite 5 with large anteromedian triangular patch of grey pruinescence (Fig. 55).

##### Diagnosis.

Body length: male 9.5–11.5 mm, female 12 mm. This is a slender, black species with infuscate wing and a subapical yellow wing mark. It can be separated from similar species by the partly bare alula, entirely microtrichose cell br, yellow wing marking situated in apical parts of cells r_4+5_ and r_2+3_.

##### Notes.

The male specimen from Suriname differs from the male holotype by its longer body (11.5 mm) and the less extensive yellow wing marking. This specimen is here considered as a colour variety of *P.pompiloides*, but this should be re-assessed if additional material becomes available.

##### Distribution.

The species is known from the Brazilian Amazon, Ecuador, and Suriname.

##### Etymology.

The specific epithet is an adjective derived from the noun *pompilus*, literally meaning ‘*pompilus*-like’. The name is inspired by the resemblance of this species to certain Neotropical spider wasps (Hymenoptera: Pompilidae).

#### Peradon satyricus

Taxon classificationAnimaliaDipteraSyrphidae

Reemer

D969F4A3-CDA9-5097-AF41-A9FA940D01B6

Figs 48 , 49 , 112 , 240–242 , 265

Peradon satyricus Reemer, 2014: 44. Holotype ♂: Surinam, Brownsberg (RMNH) [examined].

##### Studied type specimens.

Suriname • 1 ♂, holotype of *Peradonsatyricus* Reemer; Brownsberg; 04°56'45"N, 55°10'59"W; 2 Apr. 2006; M. Reemer leg.; RMNH. Label 1: “SURINAME. Brownsberg / 04°56'45"N, 55°10'59"W / 2.iv.2006. M. Reemer”. Coll. RMNH.

French Guyana • 1 ♂, paratype of *Peradonsatyricus* Reemer; Montagne de Kaw, Piste Lallane; C.M.T. Raper & A. Nelid leg.; RMNH.

##### Additional specimens.

Brazil • 1 ♂; Amazonas, Reserva Ducke, 26 km N of Manaus; 31 Aug. 1982; J.A. Rafael leg.; INPA.

##### Diagnosis.

Body length: male: 8 mm (female unknown). A sturdy, entirely black species without pale wing markings, without golden pile on thorax or abdomen, with a produced vertex. Male genitalia as in Fig. 265.

##### Notes.

In the specimen from Brazil the vertex is slightly less produced than in the type material, the median bulge on the face is somewhat less prominent, and the scutellar calcars are more or less straight (as opposed to curved and converging in the type specimens). The taxonomic value of these characters can only be assessed when further specimens become available.

##### Distribution.

Known from the Brazilian state Amazonas, French Guiana, and Suriname.

#### Peradon sciarus

Taxon classificationAnimaliaDipteraSyrphidae

Reemer

3E8EFA80-14ED-59E9-9878-8C9F3ECB5420

Figs 52 , 113 , 243–245 , 266

Peradon sciarus Reemer, 2014: 45. Holotype ♂: Surinam, Awarradam (RMNH). [examined]

##### Studied type specimens.

Suriname • 1 ♂, holotype of *Peradonsciarus* Reemer; Awarradam, along Gran Rio River; 03°50'41"N, 55°36'48"W; 13 Apr. 2006; M. Reemer leg.; RMNH. Label 1: “SURINAME. Awarradam / along Gran Rio river. / 03°50'41"N, 55°36'48"W / 13.iv.2006 / M. Reemer” • 1 ♂, paratype of *Peradonsciarus* Reemer; same data as holotype.

French Guiana • 1 ♂, paratype of *Peradonsciarus* Reemer; Roura, Kaw road, PK 37, Relais Patawa; 04°32'42"N, 52°09'09"W; Nov. 2008; J.A. Cerda leg.; RMNH.

##### Additional specimens.

Colombia • 1 ♂; Caqueta, 10 km south Florencia; 23 Jan. 1969; R.E. Dietz leg.; USNM.

Peru • 1 ♂; San Martin, around San Roque de Cumbaza; 7°23'4.96"S, 76°25'53.4"W; 15–31 Jan. 2015; T. Faasen leg.; RMNH (DNA voucher MR566 / CNC464839).

##### Studied specimens of *P.* cf. *sciarus*.

Peru • 1 ♀; San Martin Prov. 23 km S Picota, Concervacion Mun. Zona Barreal; 07°04.88'S, 76°18.89'W; 335 m a.s.l.; M.E. Irwin & J.D. Vasquez leg.; CSCA.

##### Diagnosis.

Body length: male 10–12 mm. Males are entirely black without pale wing markings, the face is black medially, and there is no fascia of golden pile along the mesonotal transverse suture. Males are clearly more slender than *P.satyricus*, which has a produced vertex.

The female is not known with certainty, but one specimen from Peru possibly belongs to this species. In contrast with the male, the female has large yellowish white subapical wing patches (Figs 244, 245), which make it look like *P.niger*, *P.normalis* and *P.pompiloides*. From *P.niger* it differs by the partly bare alula. From *P.normalis* it differs by the absence of a fascia of golden pile along the mesonotal transverse suture, the presence of a large basomedian patch of greyish pruinescence on tergite 4, and the absence of such pruinescence on the basal 1/3 of tergite 3. From *P.pompiloides* it differs by the partly bare wing cell br.

##### Notes.

The male specimens from Colombia and Peru are 1–2 mm larger than the male type specimens from Surinam and French Guiana, and their wings are a little darker.

The genitalia figured in Fig. 266 are those of the holotype. Reemer (2014) figures the genitalia of the paratype, but in less detail and also from a slightly oblique angle, instead from a proper lateral view. The figure in the present paper should be considered more accurate.

The female from Peru here identified as P.cf.sciarus is assigned to this species based on the COI barcode, which is almost identical to that of a Peruvian male of *P.sciarus*, from which it differs in one single nucleotide (Fig. 8). The most striking difference of this female with the males is the presence of a large yellowish white subapical wing patch (Figs 244, 245). Similar cases of sexual dimorphism in wing colouration occur in other *Peradon* species (e.g., *P.chrysopygus*), so this does not contradict the hypothesis that this female belongs to *P.sciarus*. This female is also similar to the males in other characters, such as the absence of a fascia of golden pile along the mesonotal transverse suture, the partially bare wing cell br and the partly bare alula.

##### Distribution.

Known from Colombia, French Guiana, Peru, and Suriname.

#### Peradon surinamensis

Taxon classificationAnimaliaDipteraSyrphidae

Reemer sp. nov.

24A81469-730A-51BA-A0DF-A32DE02C302D

http://zoobank.org/F769E98D-09A4-43F1-B201-95BCB12FD86B

Figs 76 , 77 , 87 , 89 , 91 , 93 , 139 , 140 , 246–251 , 278

Microdon flavofascium Curran: Van Doesburg 1966: 80.
Peradon flavofascium (Curran): Reemer 2014: 43.

##### Type material.

***Holotype*.** Suriname • 1 ♂, holotype of *Peradonsurinamensis* sp. nov.; Coppename River, Raleigh Falls; 16 Jul. 1963; P.H. van Doesburg Jr. leg; RMNH. Label 1: “Suriname / Coppename Riv. / Raleigh Falls / 16 July 1963 / P.H. v. Doesburg Jr.”; label 2: “Microdon [male symbol] / flavofascium Curr. / det. v. Doesburg”; label 3: “Peradon / cf. flavofascium / Det. M. Reemer 2016 / Voucher code MR033”.

Suriname • 1 ♀, paratype of *Peradonsurinamensis* sp. nov.; Distr. Brokopondo, Brownsberg N.P., Witti Kreek; 20 Jul. –3 Aug. 2001; N. Grol & N. Marseille leg.; RMNH •1 ♀, paratype of *Peradonsurinamensis* sp. nov.; Distr. Para, Colakreek, 5 km SE Zanderij; 05°27'58"N, 55°13'47"W; 1 Mar. 2006; M. Reemer leg.; RMNH.

##### Additional specimens.

Brazil • 1 ♀; Rondonia, Vilhena; 13 Nov. 1986; C. Elias leg.; UFPR; 1 ♀; Amazonas [“Amazon / 66 53”]; NHMUK.

##### Description

**(based on holotype). Adult male.** Body size: 8 mm.

***Head*.** Face occupying approximately 0.45 of head width in frontal view; black except for pale yellow, oblique yellow maculae ventrolaterally; with white pilosity. Gena black; with white pilosity. Lateral oral margin weakly produced; black; with white pilosity. Frons and vertex black; with pale golden yellow pile pilosity. Occiput black; with pale golden yellow pilosity dorsally, white pilosity ventrally. Eye bare. Antennal fossa ca. as wide as high. Antenna brown. Ratio of scape:basoflagellomere approximately 1:1.1. Basoflagellomere parallel-sided with rounded apex. Arista slender, approx. 2/3 of length of basoflagellomere.

***Thorax*.** Mesoscutum dull black; short black pile, except for narrow fasciae of short pale golden yellow pile along anterior and posterior margin and transverse suture. Postpronotum brown; bare. Postalar callus blackish brown; yellow pilose. Scutellum black with faint blue green metallic shine, posterior margin yellow; white pilose; with two apical calcars of approx. 1/5 of length of scutellum, with mutual distance approximately equal to length of scutellum. Pleura blackish brown. Anterior and posterior part of anepisternum separated by deep sulcus; white pilose anteriorly and posteriorly, with wide bare area in between. Anepimeron entirely whitish pilose. Katepisternum white pile dorsally, bare ventrally. Other pleura bare (except for microtrichia). Calypter and halter yellow.

***Wing*.** Hyaline, except for pale yellow macula in basal half of cell r_4+5_, which anteriorly extends into cell r_2+3_ and posteriorly into cell dm; somewhat infuscated around crossvein r-m and posterior appendix of vein R_4+5_. Wing microtrichose, except bare on basal 1/2 of cell c, basal 1/4 of r_1_, basal 3/4 of br, basal 4/5 of bm, anterobasal 1/3 of cup, and most of alula (only microtrichose along margins).

***Legs*.** Shining brown; white pilose, except tarsi ventrally golden yellow pilose.

***Abdomen*.** Elongate, widest at apex of tergite 2, tergite 3 parallel-sided; blackish brown, tergite 4 somewhat paler; tergite 1 white pile; tergites 2 and 3 short black pile, except for longer silvery white pile laterally; tergite 4 silvery white. Sternites brown; white pile, except sternite 1 bare. Genitalia as in Fig. 278.

**Female.** As male, except for following differences. Body length 9 mm. Vertex black pilose medially. Pale wing macula a little more extensive anteriorly and posteriorly, and infuscation around crossvein r-m and posterior appendix of vein R_4+5_ more pronounced. Tergite 5 silvery white pilose.

##### Diagnosis.

Body length: male 8 mm, female 9–10.5 mm. A rather small species of *Peradon* with a yellow macula in the wing, silvery white pile on tergite 4 (and 5 in the female) and a bare postpronotum. These three characters are only shared with *P.flavofascium*, from which it differs as follows: alula only narrowly microtrichose along margins (bare for maximally 30% in *P.flavofascium*), male with yellow wing macula extending to posterior part of cell r_2+3_ anteriorly, female with face occupying 0.45 of head width. See key for additional characters.

##### Distribution.

The species is known from Suriname, the Brazilian state Rondonia and an unknown locality in the Brazilian Amazon region.

##### Etymology.

The specific epithet is an adjective referring to Suriname, the country of origin of the type specimens.

#### Peradon trilinea

Taxon classificationAnimaliaDipteraSyrphidae

(Hull)

EFBC5E47-C7D3-5E60-BA7D-C2945F9C853E

Figs 17 , 23 , 25 , 27 , 100 , 101 , 252–254 , 259

Microdon trilinea Hull, 1943: 710. Holotype ♂: Amazon (NHMUK) [examined]; Thompson et al. 1976: 67.
Peradon trilinea (Hull): Reemer and Ståhls 2013a: 146.

##### Studied type specimens.

Amazon Region • 1 ♂, holotype of *Microdontrilinea* Hull; NHMUK.

Label 1 (small, round, red-bordered): “Holo- / type”; label 2: “Amazon / 66 53”; label 3 (red): “Holotype / Microdon / trilinea / Hull”.

##### Additional specimens.

Peru • 1 ♀; Pucallpa; 19 Apr. 1962; J. Schunke leg.; NHMUK.

##### Diagnosis.

Body size: male 13 mm, female 15 mm. The triangle of golden pile on the mesoscutum place this species in a group together with *P.aureus*, *P.aureoscutus* and *P.trivittatus*. From the first two species, *P.trilinea* differs by the presence of golden pile along the anterior and lateral margins of the mesoscutum. The male differs from *P.trivittatus* by tergite 2 being parallel-sided (widened posteriorly in *P.trivittatum*), and by the presence of a bulge-like, long pilose, median tubercle on the anterior 1/3 of sternite 4 (Fig. 25) (sternite 4 evenly convex and short pilose in *P.trivittatus*). Male genitalia as in Fig. 259. The female differs from *P.trivittatus* by the strongly arched sternite 3, with a wide yellowish membrane between its posterior margin and the straight anterior margin of tergite 4 (Fig. 27).

##### Notes.

The examined female from Peru is associated with the male holotype because of the shape of tergite 2 (flatter and more parallel-sided than in *P.trivittatus*), and because of the modified sternite 3 (unmodified in *P.trivittatus*).

##### Distribution.

Know from the Brazilian Amazon and eastern Peru.

#### Peradon trivittatus

Taxon classificationAnimaliaDipteraSyrphidae

(Curran)

5A6486F6-AFDB-5FEA-87DC-F084200D1219

Figs 15 , 24 , 26 , 97 , 98 , 255 , 260

Microdon trivittatus Curran, 1925: 344. Holotype ♂: Guyana (AMNH) [examined]; Thompson et al. 1976: 67.
Peradon trivittatus (Curran): Reemer and Ståhls 2013a: 146; Reemer 2014: 46.

##### Studied type specimens.

Guyana • 1 ♂, holotype of *Microdontrivittatus* Curran; Kartabo; AMNH.

##### Additional specimens.

Brazil • 1 ♀; Ouro Puerto[?]; 20 Jun. 1978; E.M. Bratel leg.; NHMUK • 1 ♂; Amazonas, Rio Jau, Meriti, Mun. Novo Airao; 4–10 Jun. 1994; J.A. Rafael leg.; INPA • 1 ♂’; Am. Manaus ZF-03, BR174 km 41 Res. 1501; 02°27'26"S, 59°45'00"W; 17–31 Jan. 1996; L.E.F. Roche e Silva leg.; INPA • 1 ♂; Am. Manaus, Res. Biol. do Cueiras (ZF-2), km-34, trilha em frente ao LBA; 02°35'37"S, 60°12'39"W; 22 Jul. 2012; G.F.G. Miranda leg.; INPA • 1 ♂; Amazonas, Reserva Ducke, 26 km N. of Manaus; 24 Sep. 1982; J.A. Rafael leg.; INPA • 1 ♂; Am. Borba, Rio Abacaxis, Paxiúba; 04°28'48"S, 58°34'24"W; 2–4 Jun. 2008; J.A. Rafael leg.; INPA • 1 ♂; Amazonas, Florest Canutama, Terra Firme; 7 May 2013; 6.5069S, 64.5515W; Oliveira & Somavilla leg.; INPA • 1 ♀; Am. Manaus, AM010, km 54 B12; 02°45'22"S, 51°31'03"W; 22–31 Sep. 1997; INPA • 1 ♀; Amazonas, Manaus; 30 Nov.1981; J.A. Rafael leg.; INPA.

Colombia • 1 ♂; Letitia, Amazonas Pr.; 185 m a.s.l.; 19–26 Feb. 1972; D. Ward & A. Forsyth leg.; CNC [BOLD barcode specimen CNCDB1893-11] • 1 ♂; Vaupes, Miraflores; 31 Jan. –5 Feb.1972; M. Cooper leg; NHMUK • 1 ♂ 1 ♀ (“in cop.”); Meta, La Macarena; 29 Oct. –7 Nov. 1976; M. Cooper leg.; NHMUK.

French Guiana • 1 ♂; Charvein; 1914; R. Benoist leg.; MNHN • 1 ♂; Roura, Kaw Road, PK 37, Relais Patawa; 4°32'42"N, 52°9'9"W; Jan. 2008; J.A. Cerda leg.; RMNH • 1 ♀; Roura, Kaw Road, PK 37, Relais Patawa; 4°32'42"N, 52°9'9"W; Jul. 2009; J.A. Cerda leg.; RMNH.

Suriname • 1 ♂; Brownsberg; 3 Apr. 2006; M. Reemer leg.; RMNH • 1 ♂; Carolinakreek; 30 Apr. 1962; P.H. van Doesburg Jr. leg.; RMNH • 1 ♀; Perica; 11–25 Jun. 1997; B. De Dijn leg.; RMNH • 1 ♂; Raleigh Falls; 11 Jul. 1963; P.H. van Doesburg Jr. leg.; RMNH.

Guyana • 1 ♂; Mazaruni-Potaro District, Takutu Mountains; 6°15'N, 59°5'W; 6 Dec. 1983; W.E. Steiner leg.; USNM.

Peru • 1 ♂; Pucallpa; 17 Jan. 1964; J. Schunke leg.; NHMUK • 1 ♂; SAM: around San Roque de Cumbaza; 6°23'4.96"S, 76°25'53.47"W; 15–31 Jan. 2015; T. Faasen leg.; RMNH (DNA voucher CNC464837).

Suriname • 1 ♂; Carolinakreek; 30 Apr. 1962; P.H. van Doesburg Jr. leg.; RMNH • 1 ♂; Raleigh Falls; 11 Jul. 1963; P.H. van Doesburg leg.; RMNH • 1 ♂; Raleigh Vallen-Voltzberg res.; 90 m a.s.l.; 29 Jan–13 Feb. 1982; J. Carpenter & D. Trail leg.; USNM • 1 ♀; Perica; 11–25 Jun. 1997; B. De Dijn leg.; RMNH • 1 ♂; Brownsberg; 3 Apr. 2006; M. Reemer leg.; RMNH.

Venezuela • 3 ♂; T.F. Zmaz., Cerro de la Neblina, basecamp; 140 m a.s.l.; 0°50'N, 66°10'W; 10–20 Feb. 1985; P.J. & P.M. Spangler, R.A. Faitoute & W.E. Steiner leg.; USNM.

##### Diagnosis.

Body size: male 7–13 mm, female 9–14 mm. The triangle of golden pile on the mesoscutum place this species in a group together with *P.aureus*, *P.aureoscutus* and *P.trilinea*. From the first two species, *P.trivittatus* differs by the presence of golden pile along the anterior and lateral margins of the mesoscutum. The male differs from *P.trilinea* by tergite 2 being widened posteriorly (parallel-sided in *P.trilinea*), and by sternite 4 being evenly convex and short pilose (with bulge-like, long pilose tubercle in *P.trilinea*). Male genitalia as in Fig. 260. The female differs from *P.trilinea* by sternite 3 being more or less flat and only narrowly separated from sternite 4 (instead of strongly arched and with wide intermediate membrane in *P.trilinea*).

##### Notes.

Body size variation is considerable in this species. The smallest males (known from Venezuela and the Brazilian state of Amazonas) measure only 7 or 8 mm, which at first sight gives the impression that they belong to a different species. However, many intermediates occur between these ‘dwarfs’ and the largest specimens, and all specimens are very similar in morphology and colouration.

##### Distribution.

Known from Amazonian parts of Brazil, Colombia, Peru, Suriname, and Venezuela.

## Plates

**Figures 13–18.**
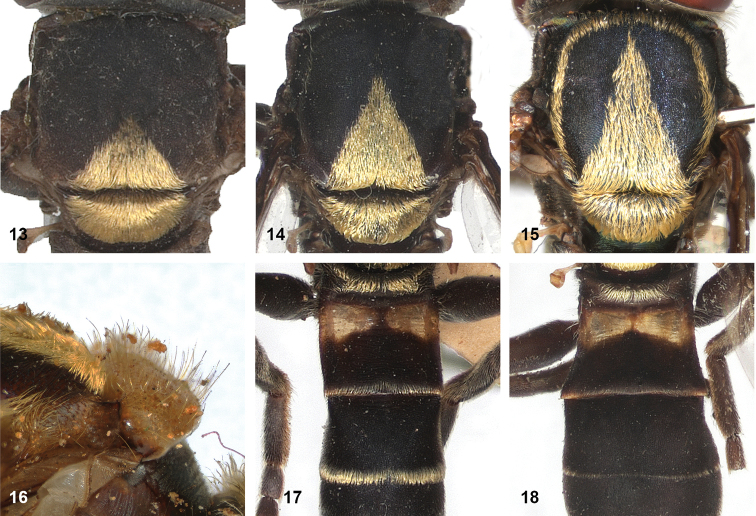
**13–15** Mesoscutum: **13***Peradonaureoscutus* male holotype **14***P.aureus* male Ecuador CNC**15***P.trivittatus* male French Guiana RMNH**16***Peradonfenestratus*, scutellum lateral **17, 18** Tergites 2 and 3: **17***Peradontrilinea* male holotype **18***P.aureus* male holotype.

**Figures 19–24.**
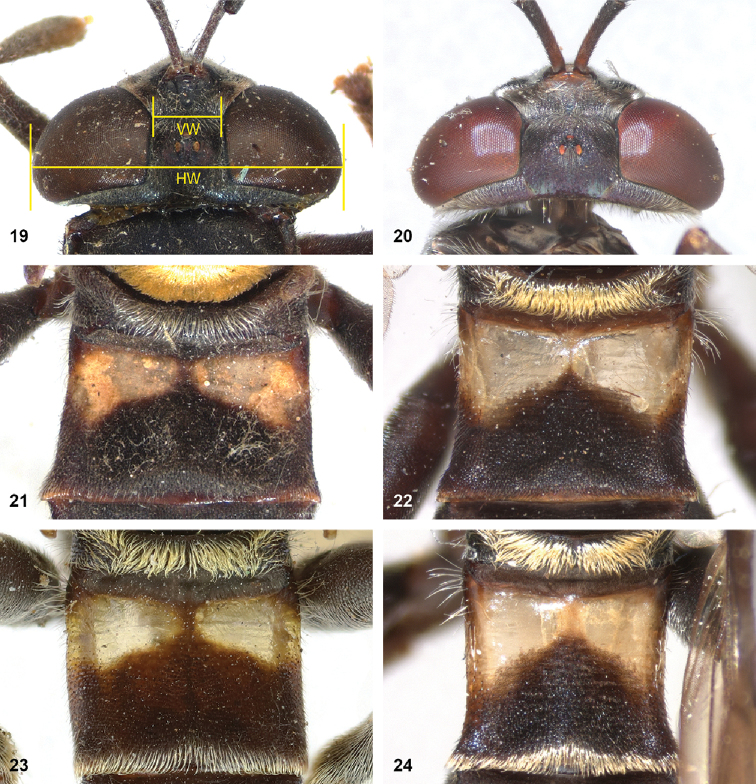
**19, 20** Head dorsal: **19***Peradonaureoscutus* male holotype (HW = head width, VW = vertex width) **20***P.aureus* male Ecuador CNC**21–24** Tergite 2 dorsal: **21***Peradonaureoscutus* male holotype **22***P.aureus* male Ecuador CNC**23***P.trilinea* male holotype **24***P.trivittatus* male French Guiana RMNH.

**Figures 25–27.**
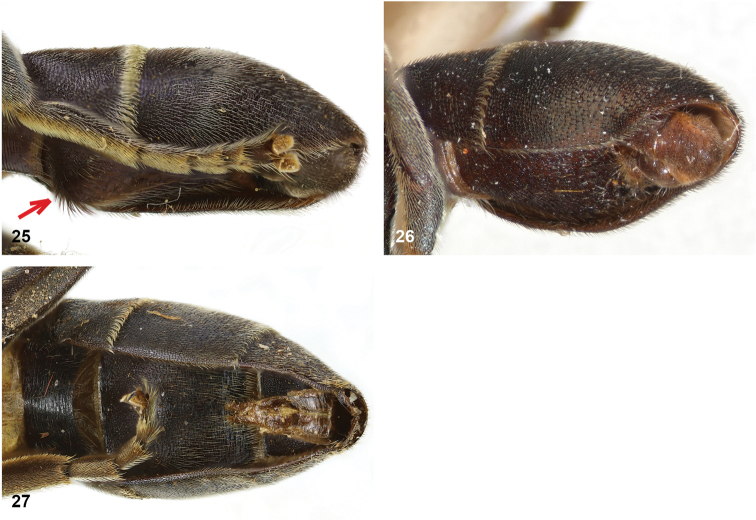
Sternite 4 ventrolateral **25***Peradontrilinea* male holotype (note anterior bulge) **26***P.trivittatus* male French Guiana RMNH**27***Peradontrilinea* female, sternites in ventral view (note wide membranous part between sternites 3 and 4).

**Figures 28–31.**
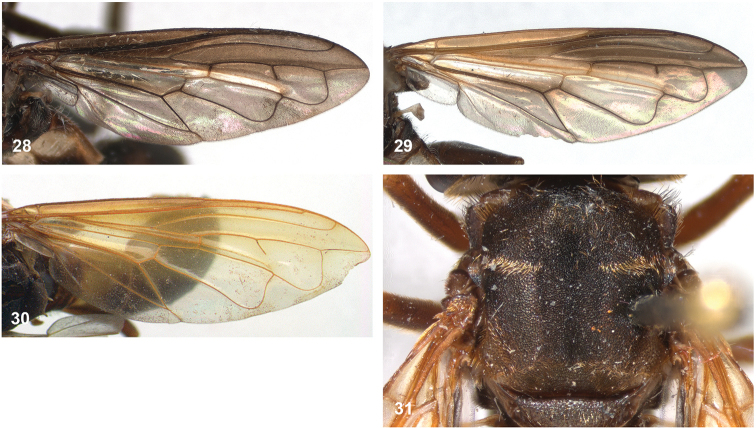
**28–30** Wings of *Peradon* species: **28***P.elongatus* male (Brazil INPA) **29***P.elongatus* female (Brazil INPA) **30***P.oligonax* female (holotype) **31***Peradonoligonax* male (Bolivia RMNH) mesonotum.

**Figures 32–35.**
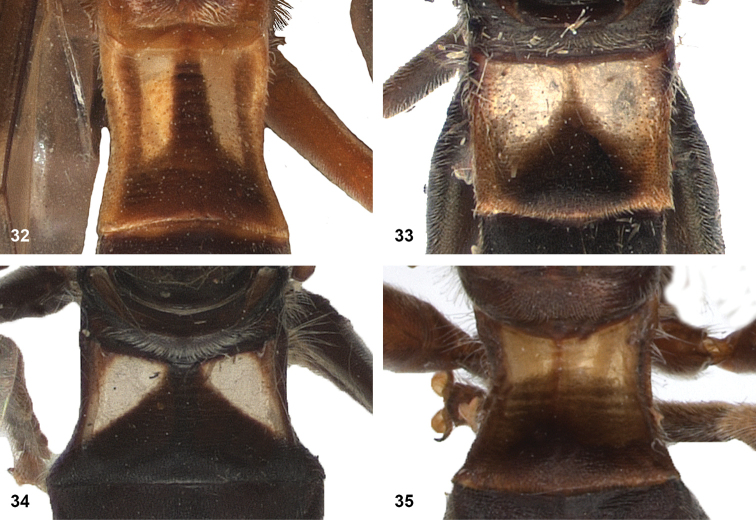
Tergite 2 dorsal **32***Peradondiaphanus* female (neotype) **33***P.elongatus* male (Brazil INPA) **34***P.hermetia* male (holotype) **35***P.hermetoides* male (holotype).

**Figures 36–47.**
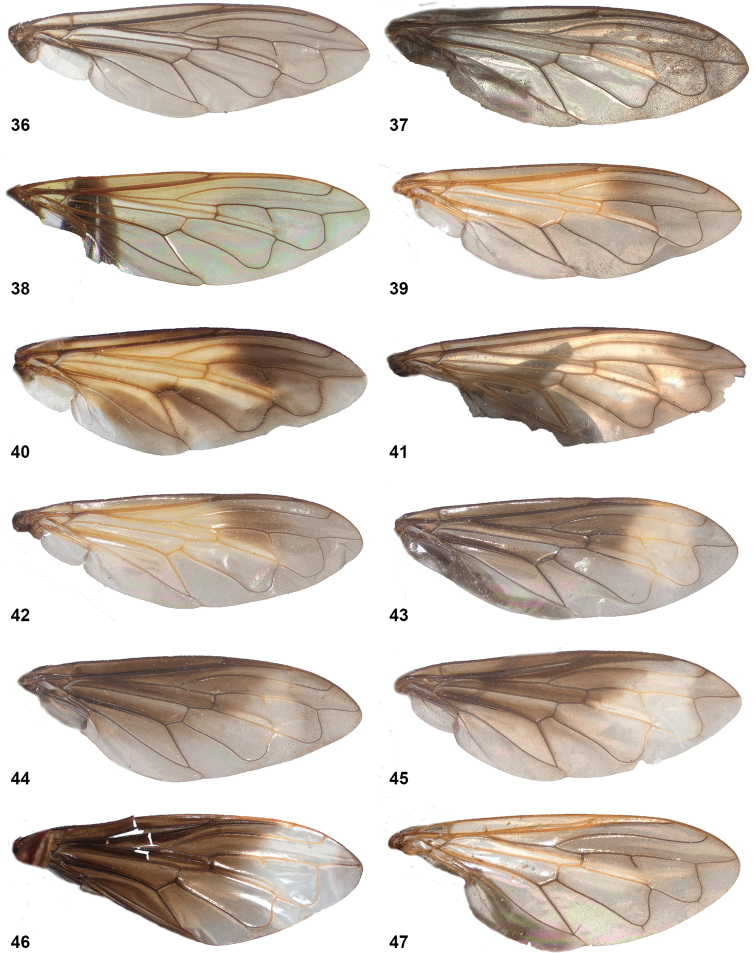
**36–46** Wings of *Peradon* species of the *bidens*-group: **36***P.bidens* ♂ Suriname RMNH**37***P.bispina* ♂ holotype **38***P.aurifascia* ♂ holotype **39***P.angustiventris* ♂ Suriname RMNH**40***P.angustus* ♀ neotype **41***P.flavipennis* ♀ holotype **42***P.normalis* var. SUR-17B ♀ Suriname RMNH**43***P.normalis* typical var. ♀ Suriname RMNH**44***P.pompiloides* ♂ holotype **45***P.pompiloides* ♀ paratype **46***P.niger* ♂ holotype **47** Wing of *P.palpator* ♂ holotype (*flavofascium* group)

**Figures 48–52.**
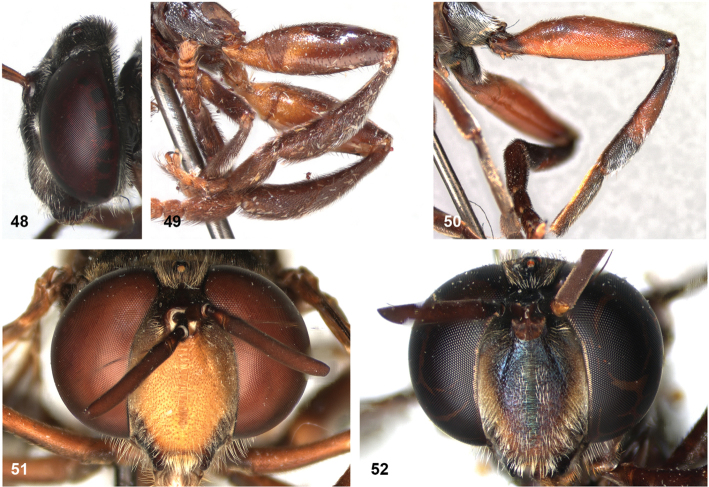
**48***Peradonsatyricus*, head profile **49, 50** Hind legs of *Peradon*: **49***P.satyricus***50***P.bidens***51, 52** Head in frontal view: **51***P.costaricensis***52***P.sciarus*.

**Figures 53–56.**
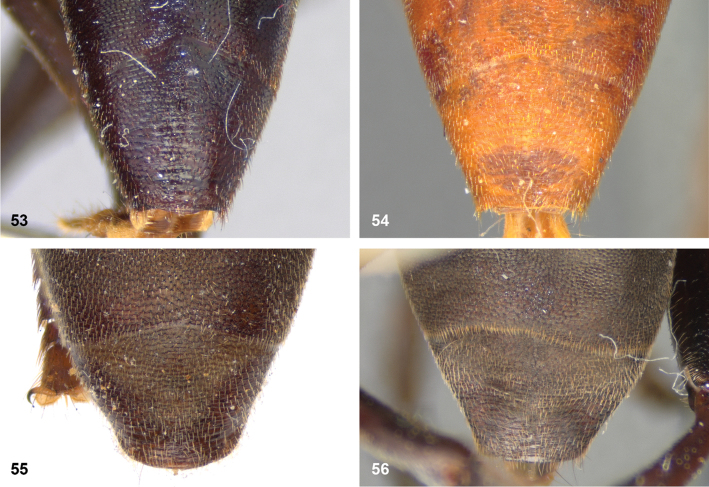
Tergite 4 of *Peradon* females **53***P.normalis* typical variation (without grey pruinescence), French Guiana RMNH**54***P.normalis* red variation (without grey pruinescence), Brazil LACM**55***P.pompiloides* paratype (with large basomedian patch of pruinescence) **56**P.cf.sciarus (with large basal area of pruinescence) Peru CSCA.

**Figures 57–60.**
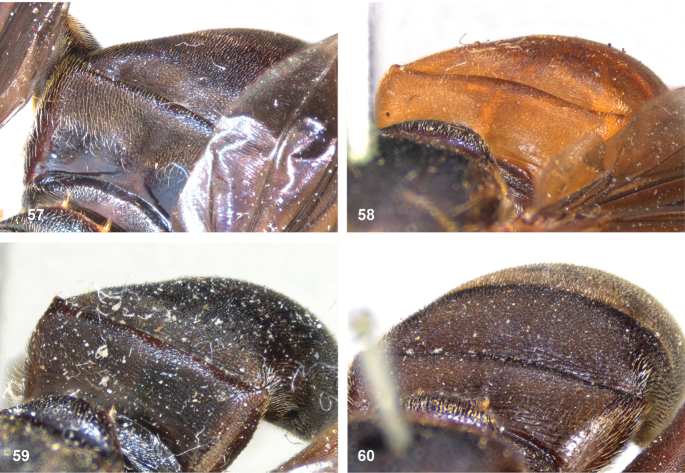
Tergite 2–3 of *Peradon* females in frontodorsal view **57***P.normalis* typical morph, French Guiana RMNH**58***P.normalis* red morph Brazil LACM**59***P.normalis* SUR-17b morph Suriname RMNH**60***P.pompiloides* paratype. Note the similar pattern of grey pruinescence on tergite 3 in all three colour morphs of *P.normalis*, with pruinescence limited to anterior 1/3 of tergite 3. In contrast, the grey pruinescence in *P.pompiloides* extends over a large part of tergite 3.

**Figures 61–66.**
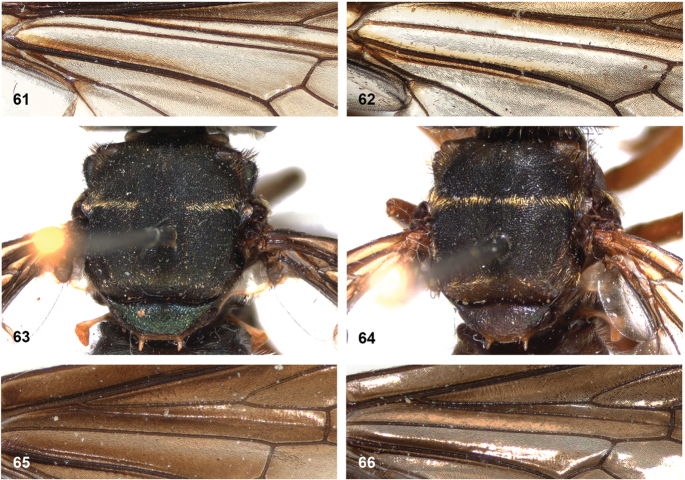
**61, 62** Wing cell bm: **61***P.bidens***62***P.costaricensis***63, 64** Mesonotal transverse fascia: **63** medially interrupted (*P.bidens*) **64** complete (*P.costaricensis*) **65, 66** Wing cell br: **65***P.pompiloides* (holotype) **66***P.normalis* (Suriname RMNH).

**Figures 67–73.**
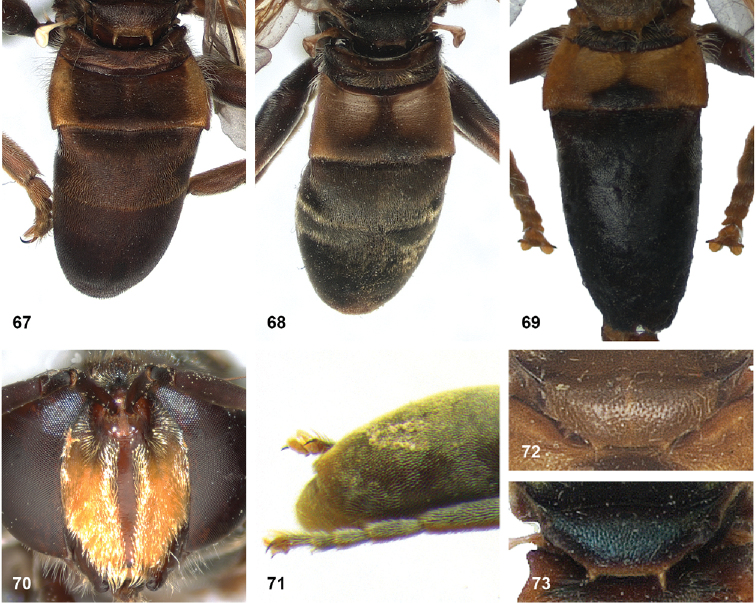
**37–39** Abdomen in dorsal view **67***P.costaricensis* male (holotype) **68***P.aurifascia* male (Brazil CSCS) **69***P.luridescens* male (Suriname RMNH) **70** Face of *P.aurifascia* male (holotype). Note smooth and bare median vitta **71** Tergite 4 of *P.aurifascia* in lateral view. Note reddish apical margin **72, 73** Scutellum: **72***P.angustiventris* female (Brazil INPA) **73***P.angustus* male (neotype).

**Figures 74–85.**
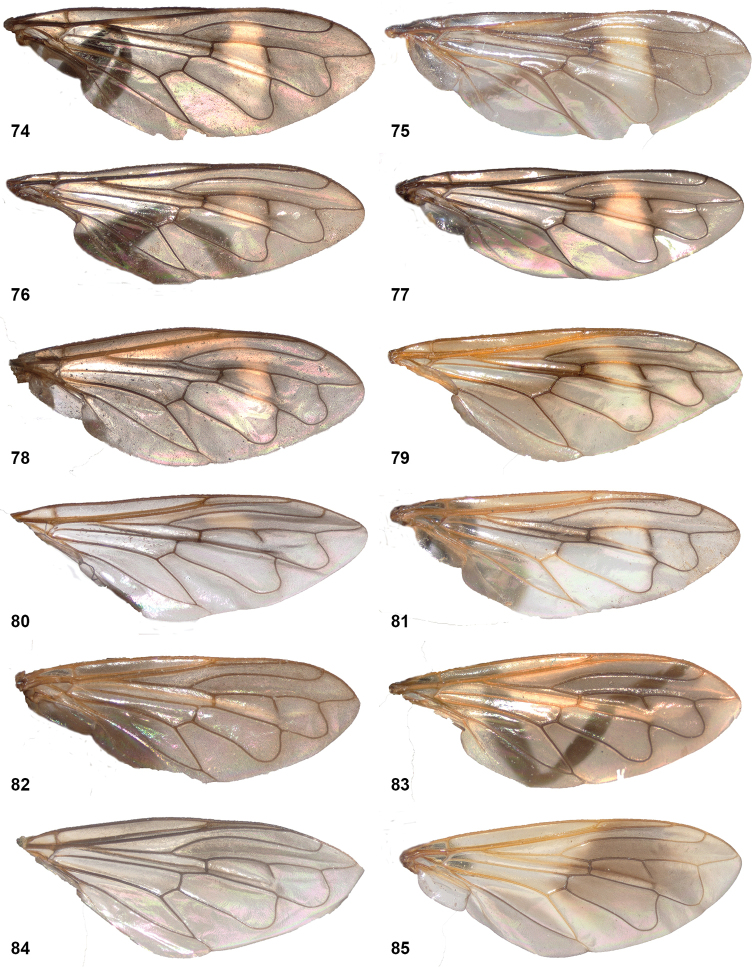
Wings of *Peradon* species of the *flavofascium*-group **74***P.flavofascium* ♂ holotype **75***P.flavofascium* ♀ **76***P.surinamensis* ♂ holotype **77***P.surinamensis* ♀ Suriname RMNH**78***P.aurigaster* ♂ holotype **79***P.aurigaster* ♀ Peru CNC**80***P.ballux* ♂ holotype **81***P.brevis* ♂ holotype **82***P.notialus* ♂ holotype **83***P.notialus* ♀ paratype **84***P.chrysopygus* ♂ Costa Rica RMNH**85***P.chrysopygus* ♀ Belize MZH.

**Figures 86–89.**
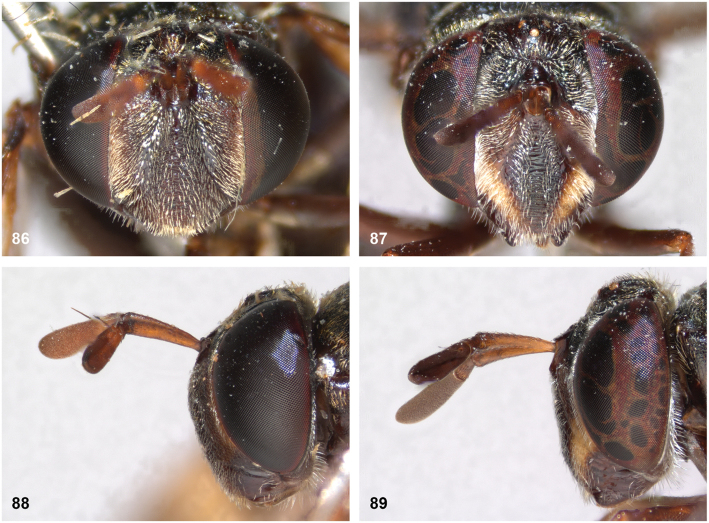
**86, 87** Heads in frontal view: **86***P.flavofascium* female (Brazil MZUSP) **87***P.surinamensis* female (paratype) **88, 89** Heads in lateral view: **88***P.flavofascium* female (Brazil MZUSP) **89***P.surinamensis* female (paratype).

**Figures 90–96.**
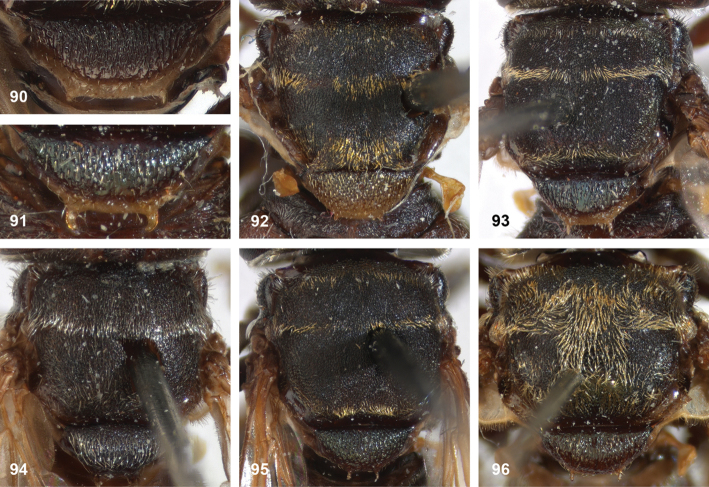
**90, 91** Scutellum: **90***P.flavofascium* male (holotype) **91***P.surinamensis* male (holotype) **92–96** Mesonotum: **92***P.flavofascium* female (Brazil MZUSP) **93***P.surinamensis* female (paratype) **94***P.notialus* male (holotype) **95***P.ballux* male (holotype) **96***P.brevis* male (holotype).

**Figures 97–104.**
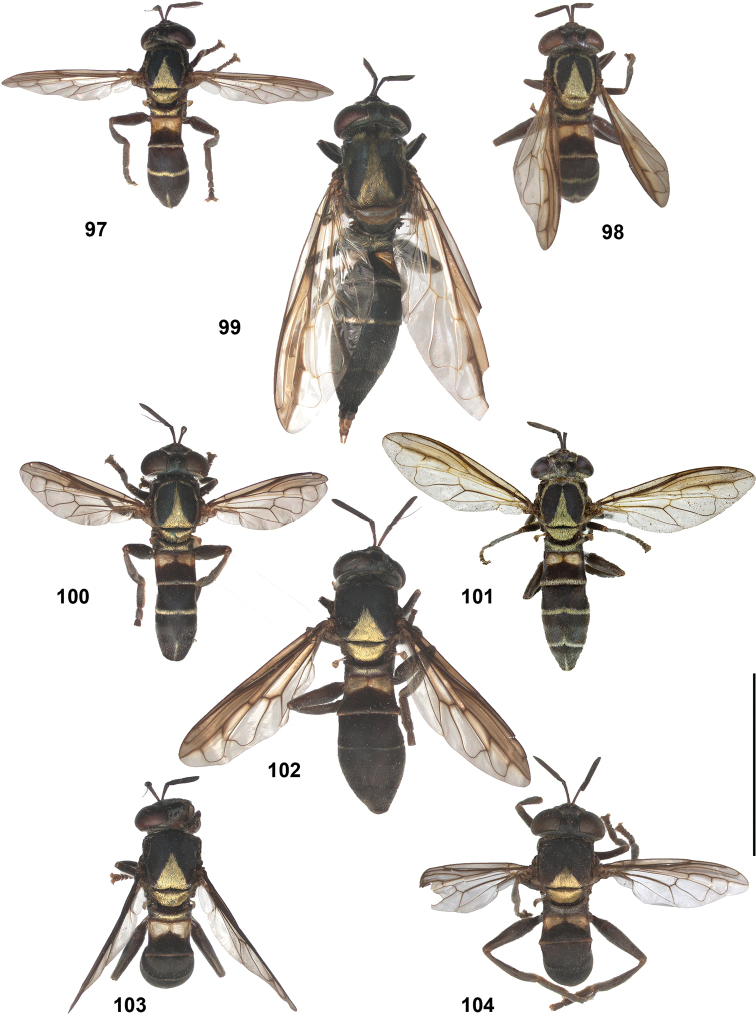
Dorsal habitus of *Peradon* species (*trivittatus* group) **97***P.trivittatus* ♂ Suriname RMNH**98***P.trivittatus* ♀ Suriname RMNH**99***P.fenestratus* ♀ Brazil INPA**100***P.trilinea* ♂ holotype **101***P.trilinea* ♀ Peru NHMUK**102***P.aureus* ♀ holotype **103***P.aureus* ♂ Ecuador CNC**104***P.aureoscutus* ♂ holotype. Scale bar: 10 mm.

**Figures 105–111.**
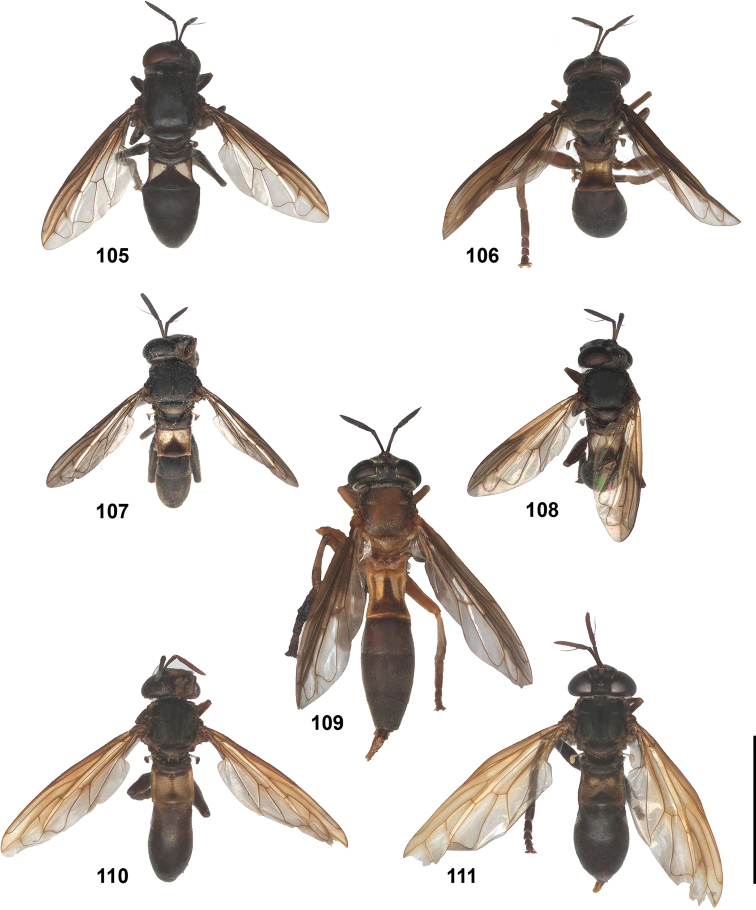
Dorsal habitus of *Peradon* species (*trivittatus* group) **105***P.hermetia* ♂ holotype **106** ♂ *P.hermetoides* holotype **107***P.elongatus* ♂ Brazil INPA**108***P.elongatus* ♀ Brazil INPA**109***P.diaphanus* ♀ neotype **110***P.oligonax* ♂ Brazil LACM**111***P.oligonax* ♀ Peru RMNH. Scale bar: 10 mm.

**Figures 112–118.**
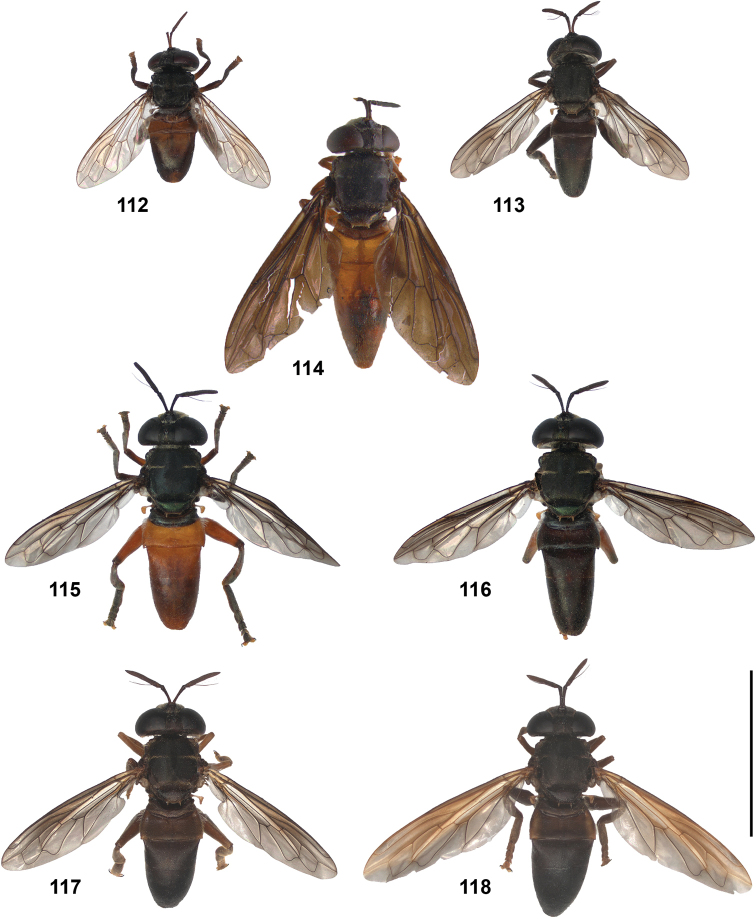
Dorsal habitus of *Peradon* species (*bidens* group) **112***P.satyricus* ♂ holotype **113***P.sciarus* ♂ holotype **114***P.bidens* typical variation ♀ (holotype *M.flavomarginatum* Curran) **115***P.bidens* typical morph ♂ Suriname RMNH**116***P.bidens* morph *langi* ♂ Suriname RMNH**117***P.costaricensis* ♂ paratype **118***P.costaricensis* ♀ paratype. Scale bar: 10 mm.

**Figures 119–128.**
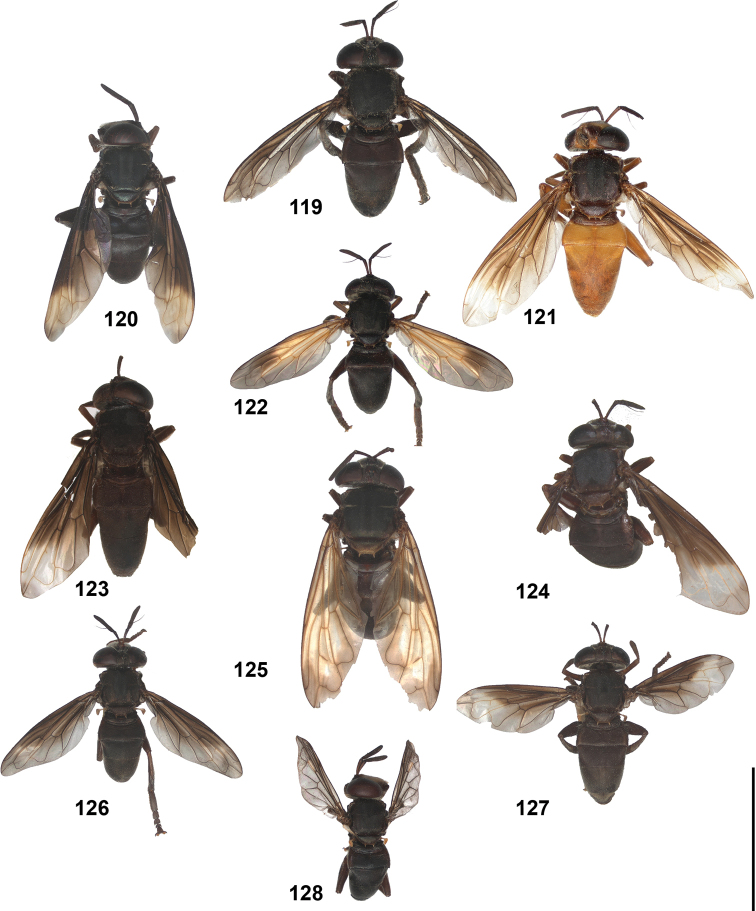
Dorsal habitus of *Peradon* species (*bidens* group) **119**P.?normalis ♂ Brazil Rondonia LACM**120***P.normalis* typical morph ♀ French Guiana RMNH**121***P.normalis* red morph ♀ Brazil Pará LACM**122***P.normalis* SUR-17b morph ♀ **123***P.niger* ♂ holotype **124***P.niger* ♀ (holotype *M.manni* Shannon) **125***P.flavipennis* ♀ holotype **126***P.pompiloides* ♂ holotype **127***P.pompiloides* ♀ paratype **128***P.bispina* ♂ Brazil CNC. Scale bar: 10 mm.

**Figures 129–135.**
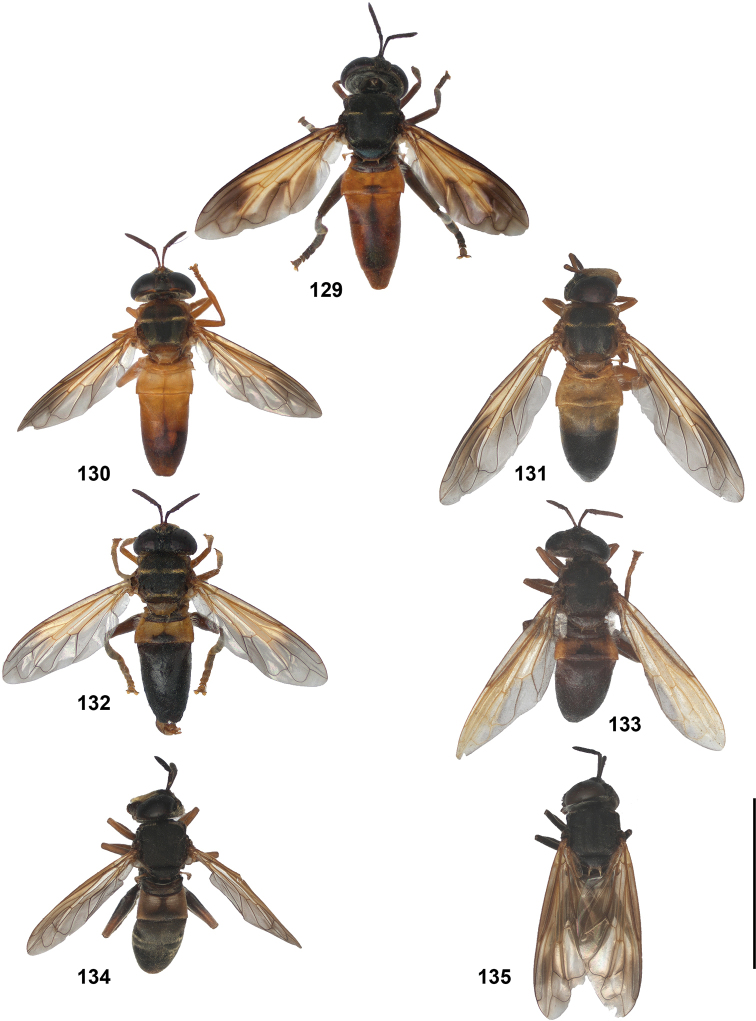
Dorsal habitus of *Peradon* species (*bidens* group) **129***P.angustus* ♀ neotype **130***P.angustiventris* ♂ Suriname RMNH**131***P.angustiventris* ♀ Brazil Manaus INPA**132***P.luridescens* ♂ Suriname RMNH**133***P.luridescens* ♀ Suriname RMNH**134***P.aurifascia* ♂ Brazil CSCA**135***P.aurifascia* ♀ Brazil MZUSP. Scale bar: 10 mm.

**Figures 136–142.**
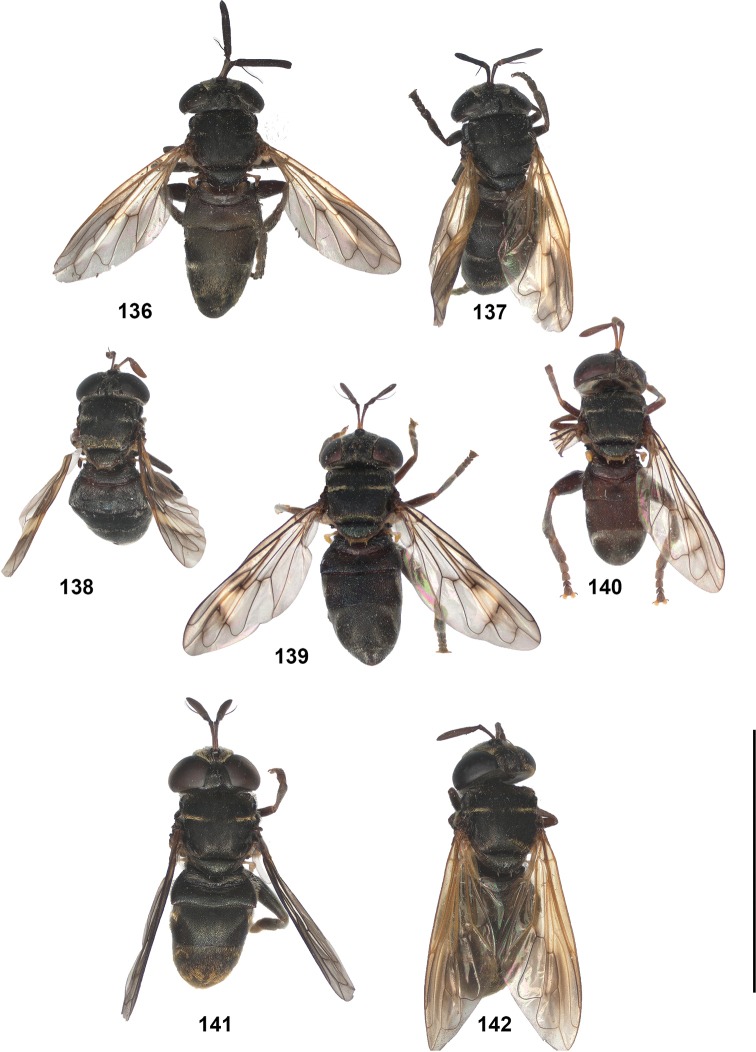
Dorsal habitus of *Peradon* species (*flavofascium* group) **136***P.palpator* ♂ holotype **137***P.palpator* ♀ paratype **138***P.flavofascium* ♂ Brazil MZUSP **139***P.surinamensis* ♀ paratype **140***P.surinamensis* ♂ holotype **141***P.chrysopygus* ♂ Costa Rica LACM**142***P.chrysopygus* ♀ Costa Rica LACM. Scale bar: 10 mm.

**Figures 143–148.**
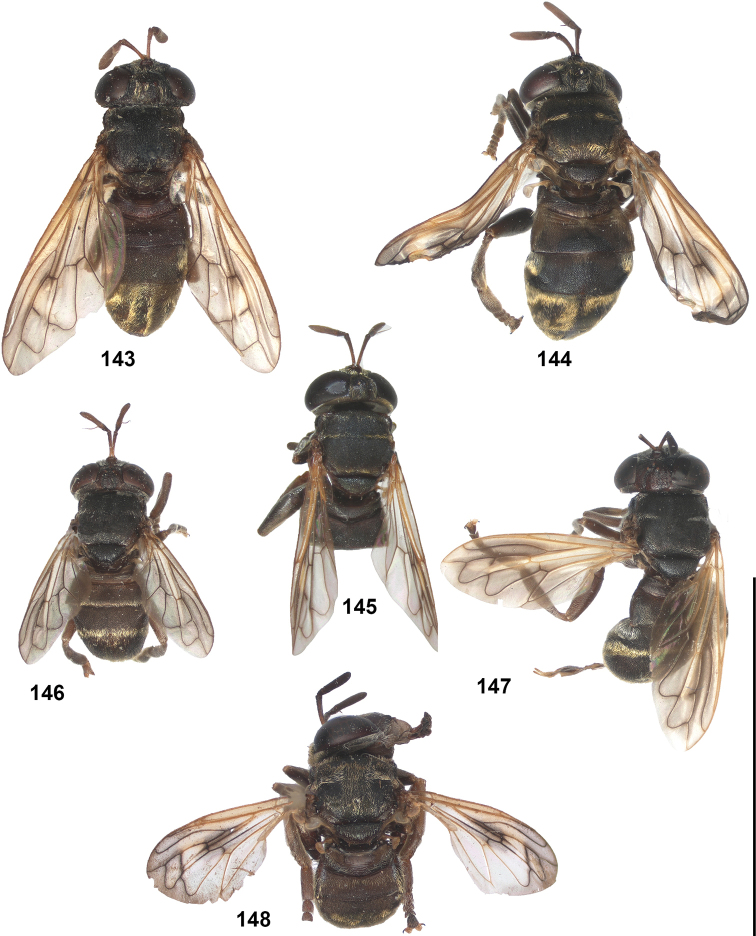
Dorsal habitus of *Peradon* species (*flavofascium* group) **143***P.aurigaster* ♂ Bolivia RMNH**144***P.aurigaster* ♀ Peru RMNH**145***P.ballux* ♂ holotype **146***P.notialus* ♂ holotype **147***P.notialus* ♀ paratype **148***P.brevis* ♂ holotype. Scale bar: 10 mm.

**Figures 149, 150.**
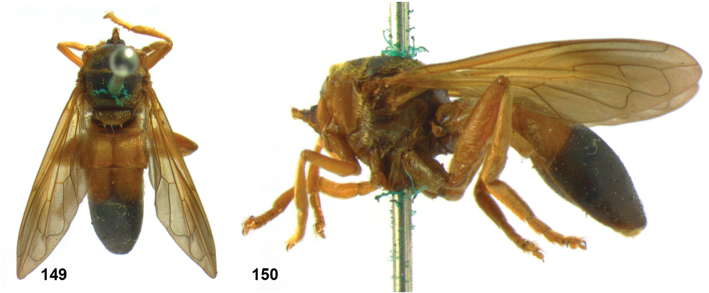
*Peradonangustiventris* male, holotype **149** habitus dorsal **150** habitus lateral.

**Figures 151, 152.**
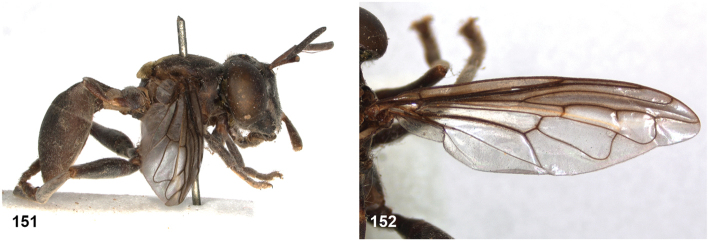
*Peradonaureoscutus* male, holotype **151** habitus lateral **152** wing.

**Figures 153–156.**
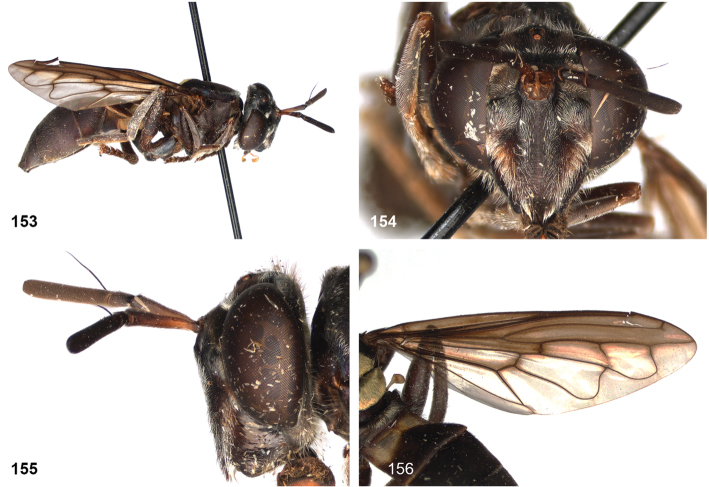
*Peradonaureus* female, holotype **153** habitus lateral **154** head frontal **155** head lateral **156** wing.

**Figures 157–160.**
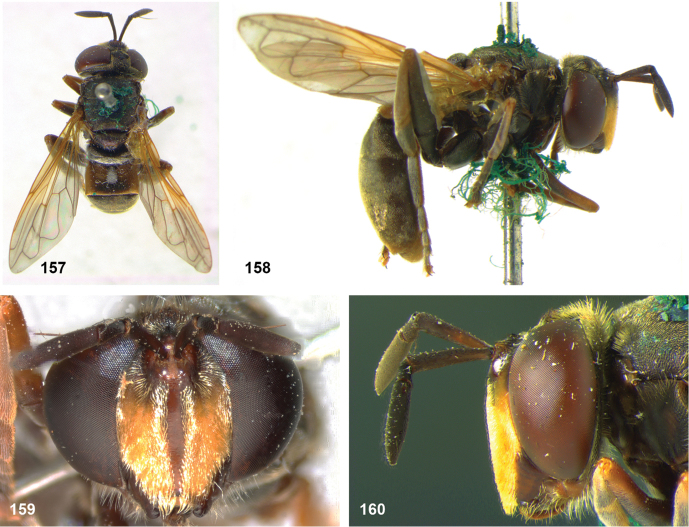
*Peradonaurifascia* male, holotype **157** habitus dorsal **158** habitus lateral **159** head frontal **160** head lateral.

**Figures 161–164.**
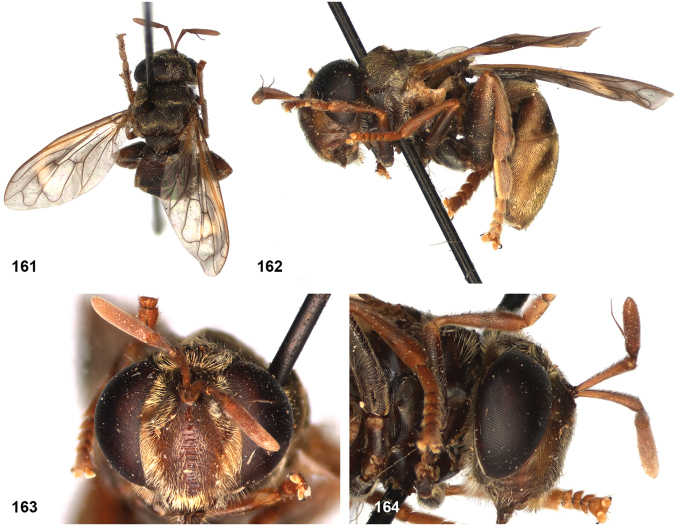
*Peradonaurigaster* male holotype **161** habitus dorsal **162** habitus lateral **163** head frontal **164** head lateral.

**Figures 165–170.**
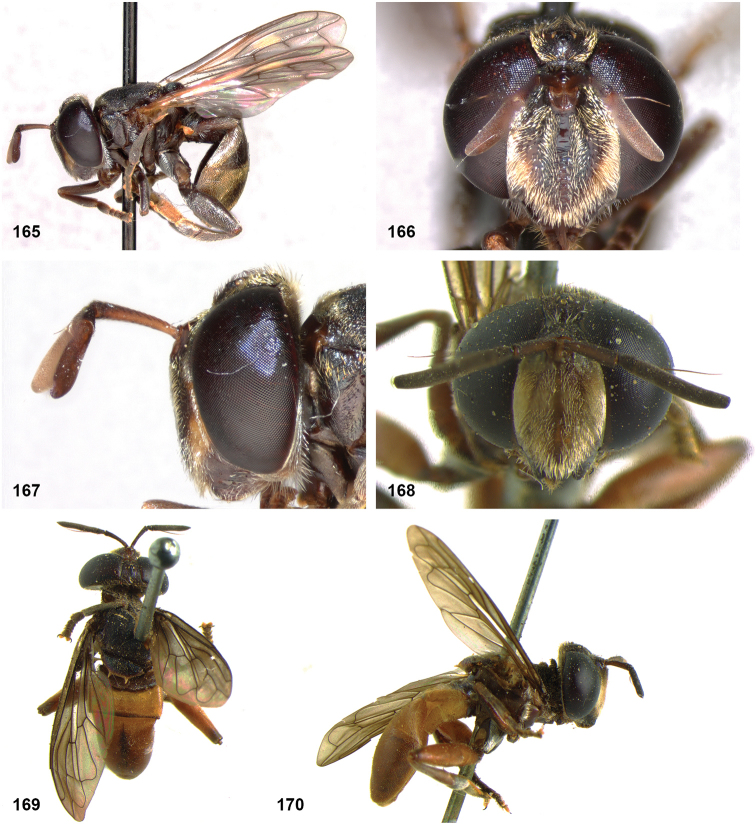
*Peradonballux* male, holotype: **165** habitus lateral **166** head frontal **167** head lateral **168–170***Peradonbidens* male, holotype **168** head frontal **169** habitus dorsal **170** habitus lateral

**Figures 171–176.**
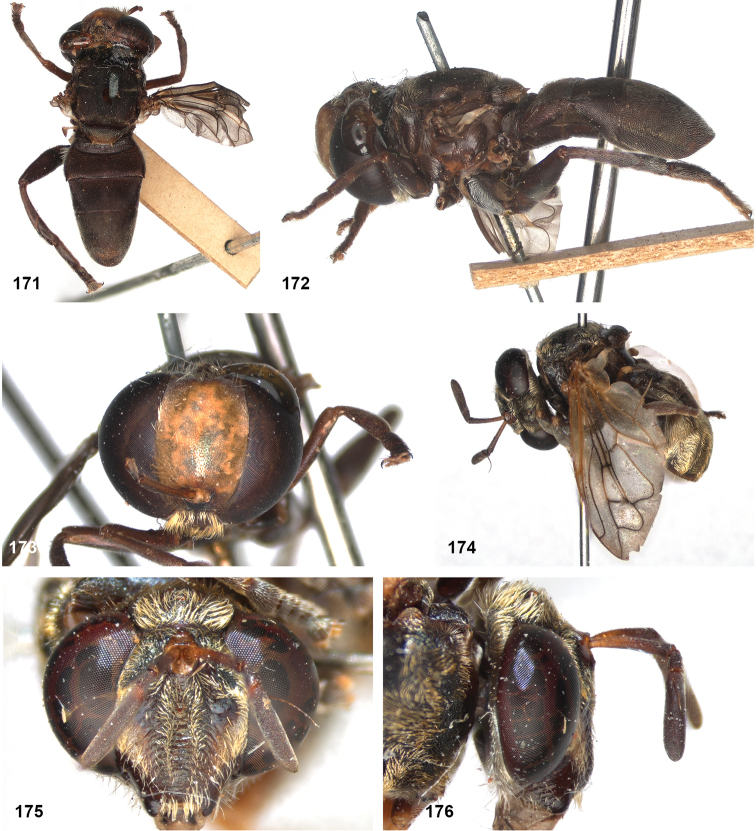
**171–173***Peradonbispina* male, holotype: **171** habitus dorsal **172** habitus lateral **173** head frontal (the head belongs to a species of another microdontine genus, see text) **174–176***Peradonbrevis* male, holotype: **174** habitus dorsal **175** head frontal **176** head lateral.

**Figures 177–179.**
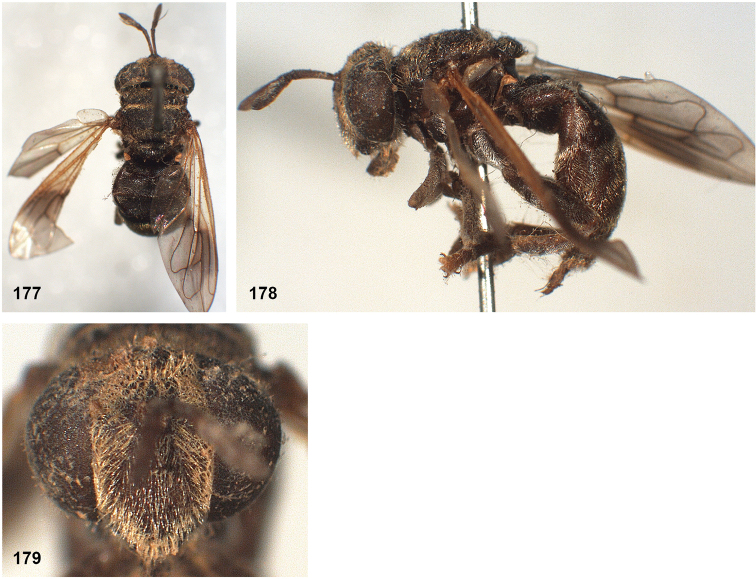
*Peradonchrysopygus* female, holotype **177** habitus dorsal **178** habitus lateral **179** head frontal.

**Figures 180–185.**
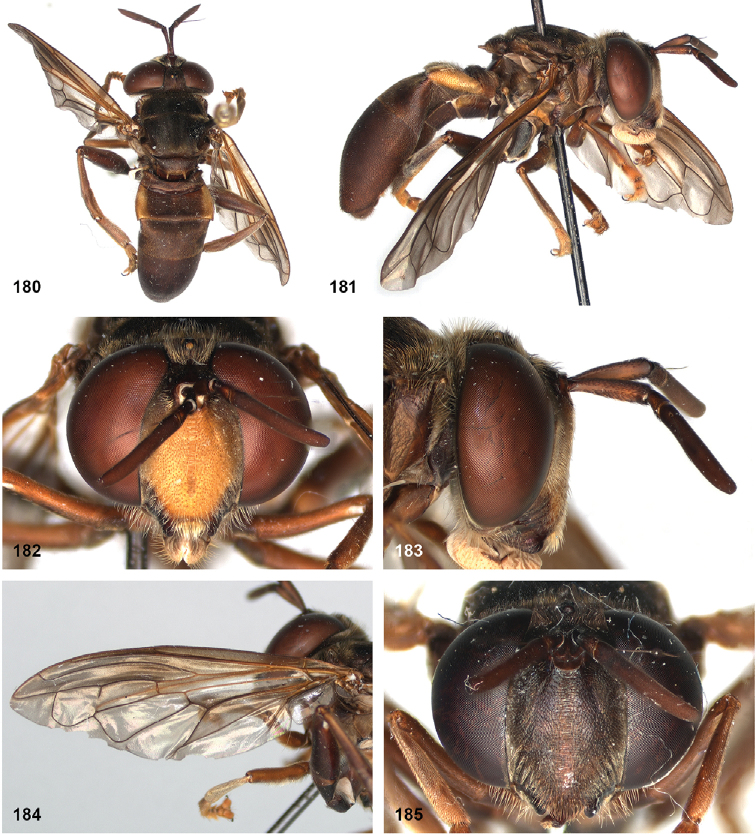
**180–184***Peradoncostaricensis* male, holotype: **180** habitus dorsal **181** habitus lateral **182** head frontal **183** head lateral **184** wing **185***Peradoncostaricensis* female, paratype: face frontal.

**Figures 186–193.**
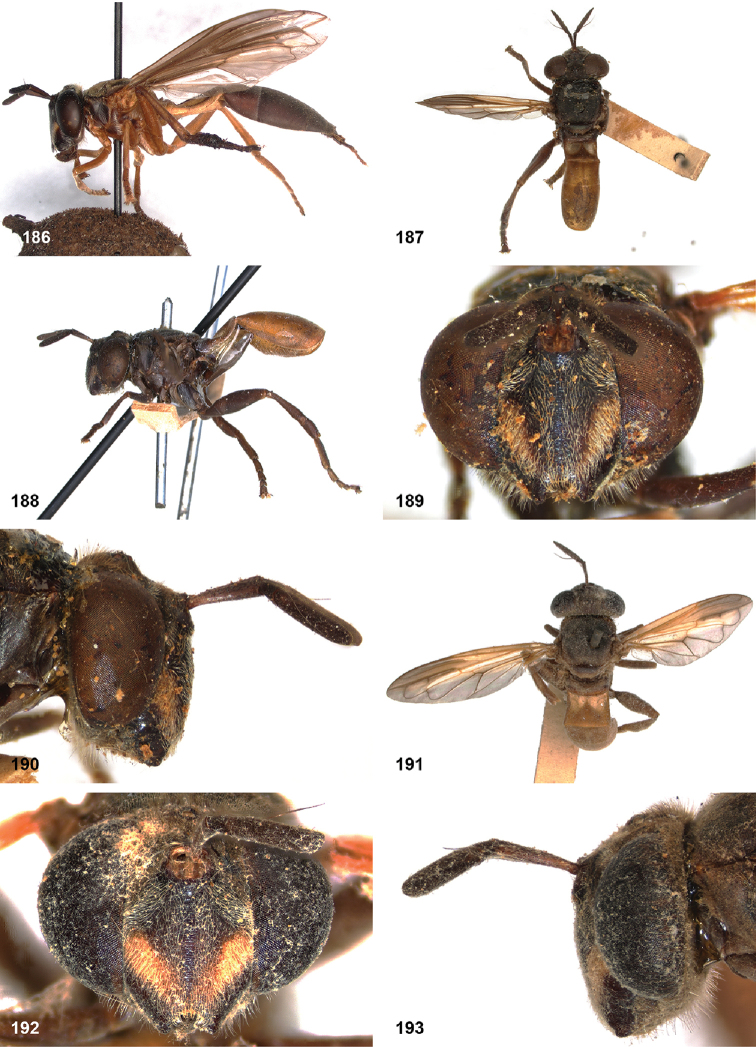
**186***Peradondiaphanus* female, neotype: habitus lateral **187–190***Peradonelongatus* male, holotype: **187** habitus dorsal **188** habitus lateral **189** head frontal **190** head lateral **191–193***Peradonelongatus* female, paratype: **191** habitus dorsal **192** head frontal **193** head lateral.

**Figures 194–198.**
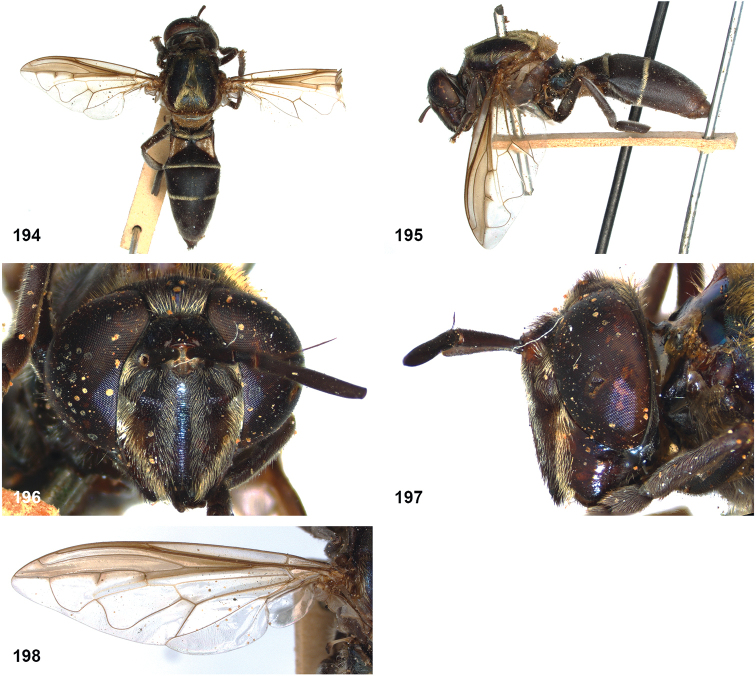
*Peradonfenestratus* male, holotype **194** habitus dorsal **195** habitus lateral **196** head frontal **197** head lateral **198** wing.

**Figures 199–205.**
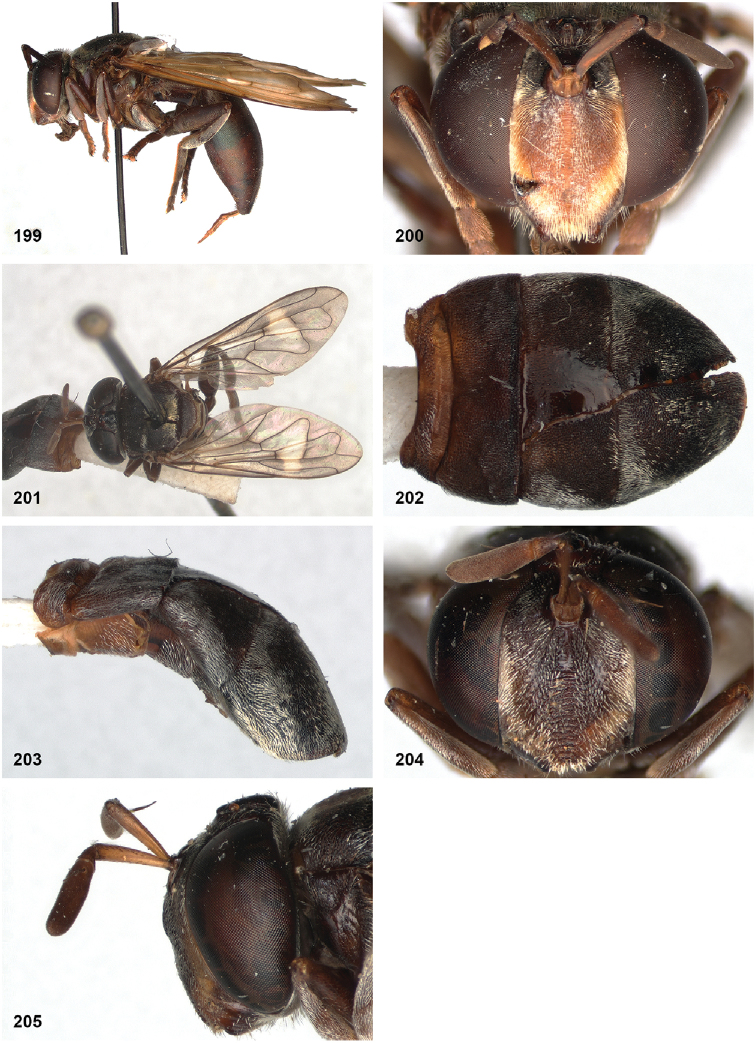
**199, 200***Peradonflavipennis* female, holotype: **199** habitus lateral **200** head frontal **201–205***Peradonflavofascium* male, holotype: **201** habitus dorsal **202** abdomen dorsal **203** abdomen lateral **204** head frontal **205** head lateral.

**Figures 206–211.**
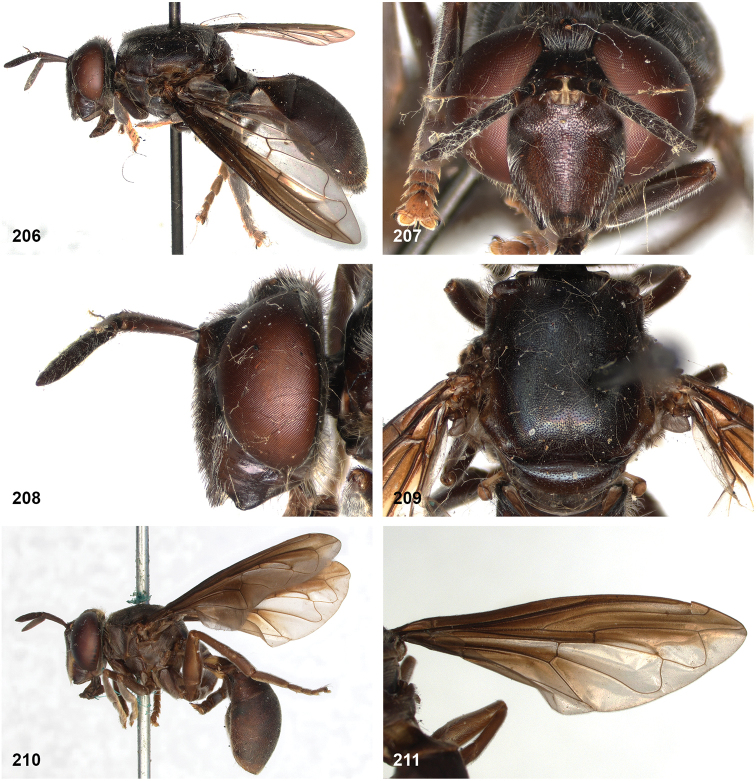
**206–209***Peradonhermetia* male, holotype: **206** habitus lateral **207** head frontal **208** head lateral **209** thorax dorsal **210, 211***Peradonhermetoides* male, holotype: **210** habitus lateral **211** wing.

**Figures 212–217.**
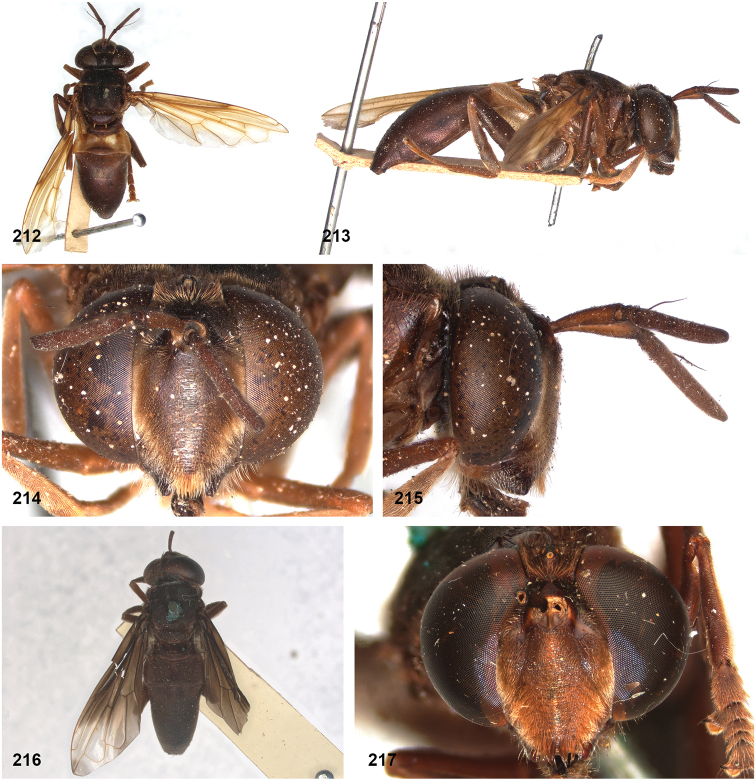
**212–215***Peradonluridescens* female, holotype: **212** habitus dorsal **213** habitus lateral **214** head frontal **215** head lateral **216, 217***Peradonniger* male, holotype: **216** habitus dorsal **217** head frontal.

**Figures 218–221.**
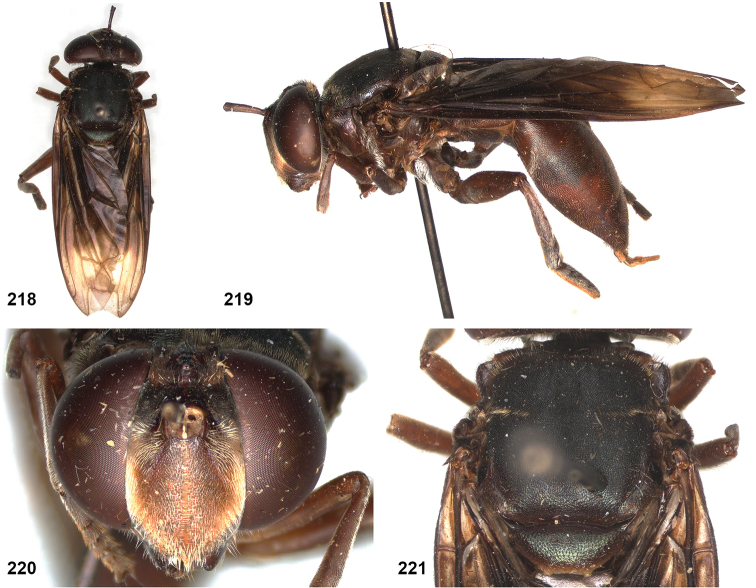
*Peradonnormalis* female, holotype **218** habitus dorsal **219** habitus lateral **220** head frontal **221** thorax dorsal.

**Figures 222–227.**
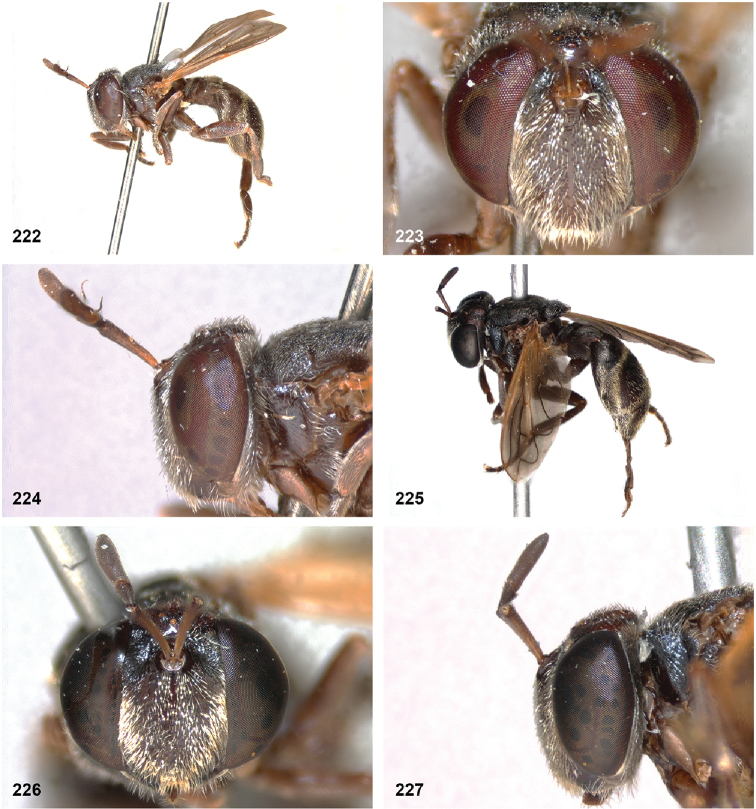
**222–224***Peradonnotialus* male, holotype: **222** habitus lateral **223** head frontal **224** head lateral **225–227***Peradonnotialus* female, holotype **225** habitus lateral **226** head frontal **227** head lateral.

**Figures 228–231.**
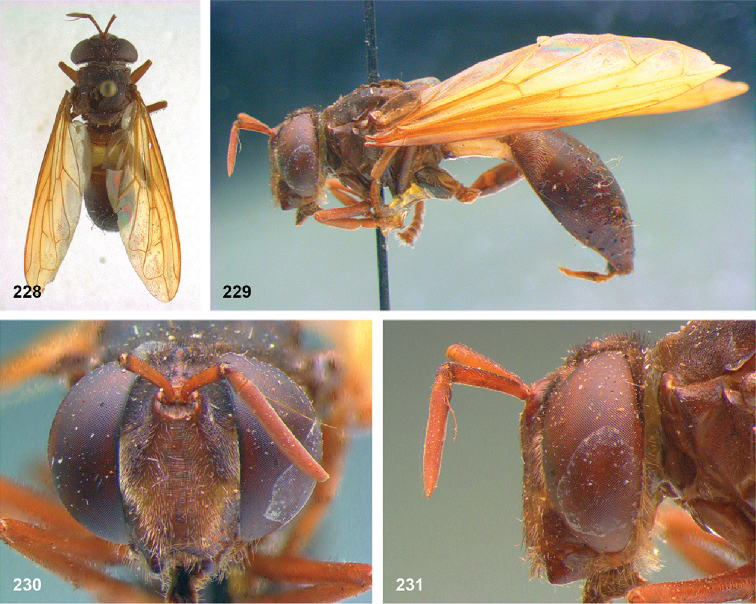
*Peradonoligonax* female, holotype **228** habitus dorsal **229** habitus lateral **230** head frontal **231** head lateral.

**Figures 232–234.**
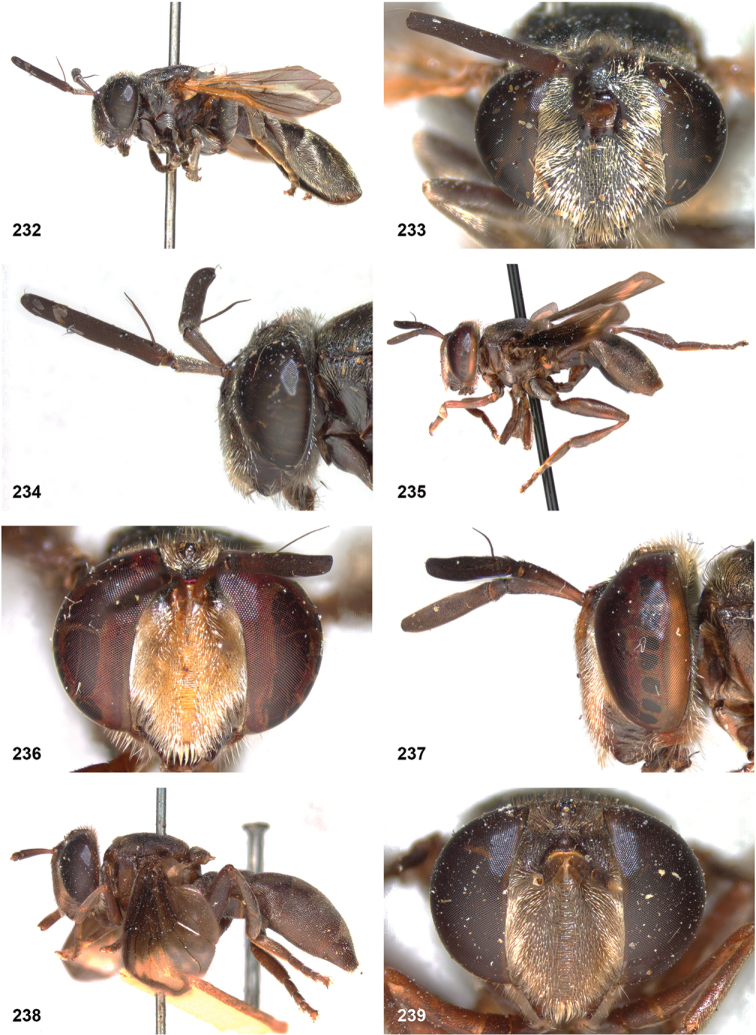
**232–234***Peradonpalpator* male, holotype **232** habitus lateral **233** head frontal **234** head lateral **235–237***Peradonpompiloides* male, holotype **235** habitus lateral **236** head frontal **237** head lateral **238–239***Peradonpompiloides* female paratype **238** habitus lateral **239** head frontal.

**Figures 240–245.**
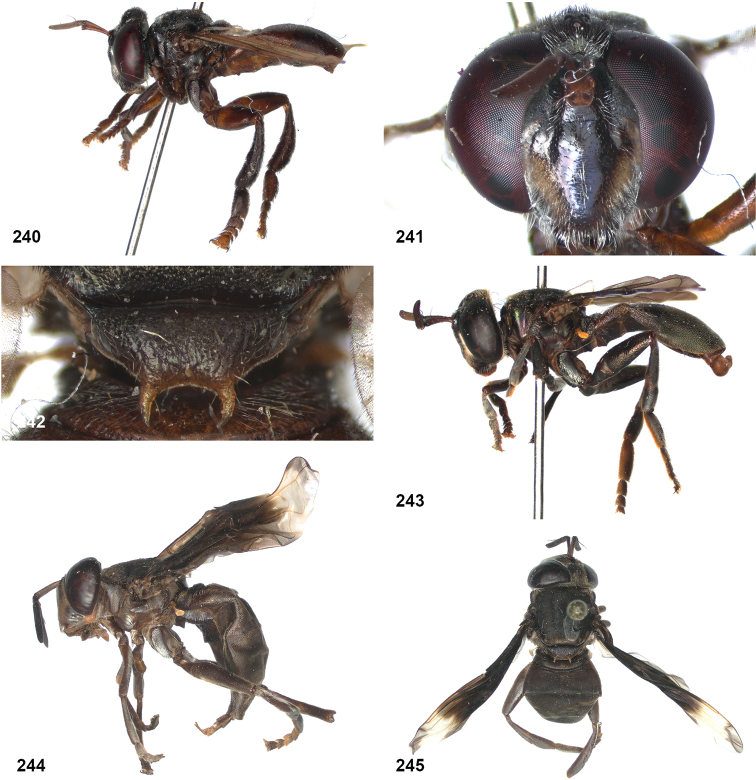
**240–242***Peradonsatyricus* male, holotype: **240** habitus lateral **241** head frontal **242** scutellum dorsal **243***Peradonsciarus* male holotype: habitus lateral **244, 245**Peradoncf.sciarus female: **244** habitus lateral **245** habitus dorsal.

**Figures 246–251.**
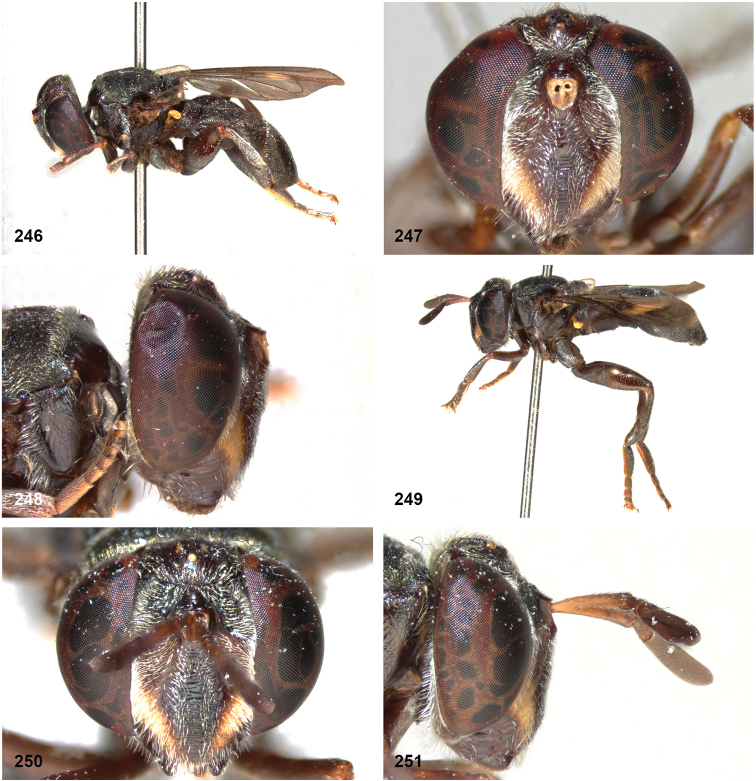
**246–248***Peradonsurinamensis* male, holotype: **246** habitus lateral **247** head frontal **248** head lateral **249–251***Peradonsurinamensis* female paratype: **249** habitus lateral **250** head frontal **251** head lateral.

**Figures 252–255.**
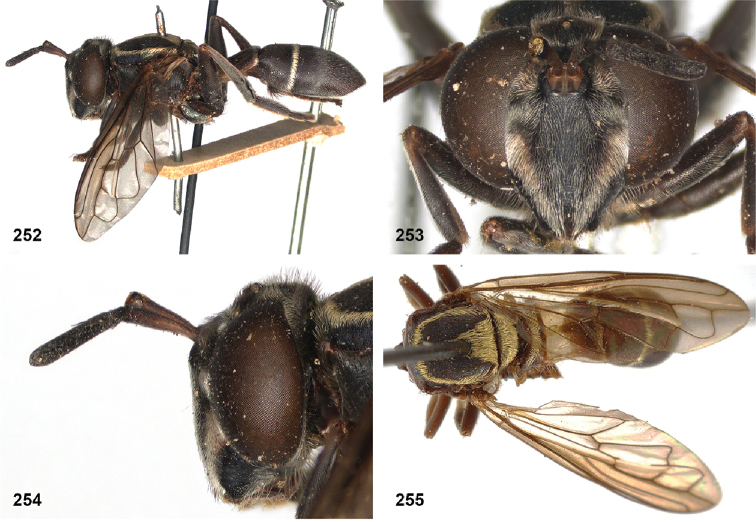
**252–254***Peradontrilinea* male, holotype: **252** habitus lateral **253** head frontal **254** head lateral **255***Peradontrivittatus* male holotype: habitus dorsal.

**Figures 256–261.**
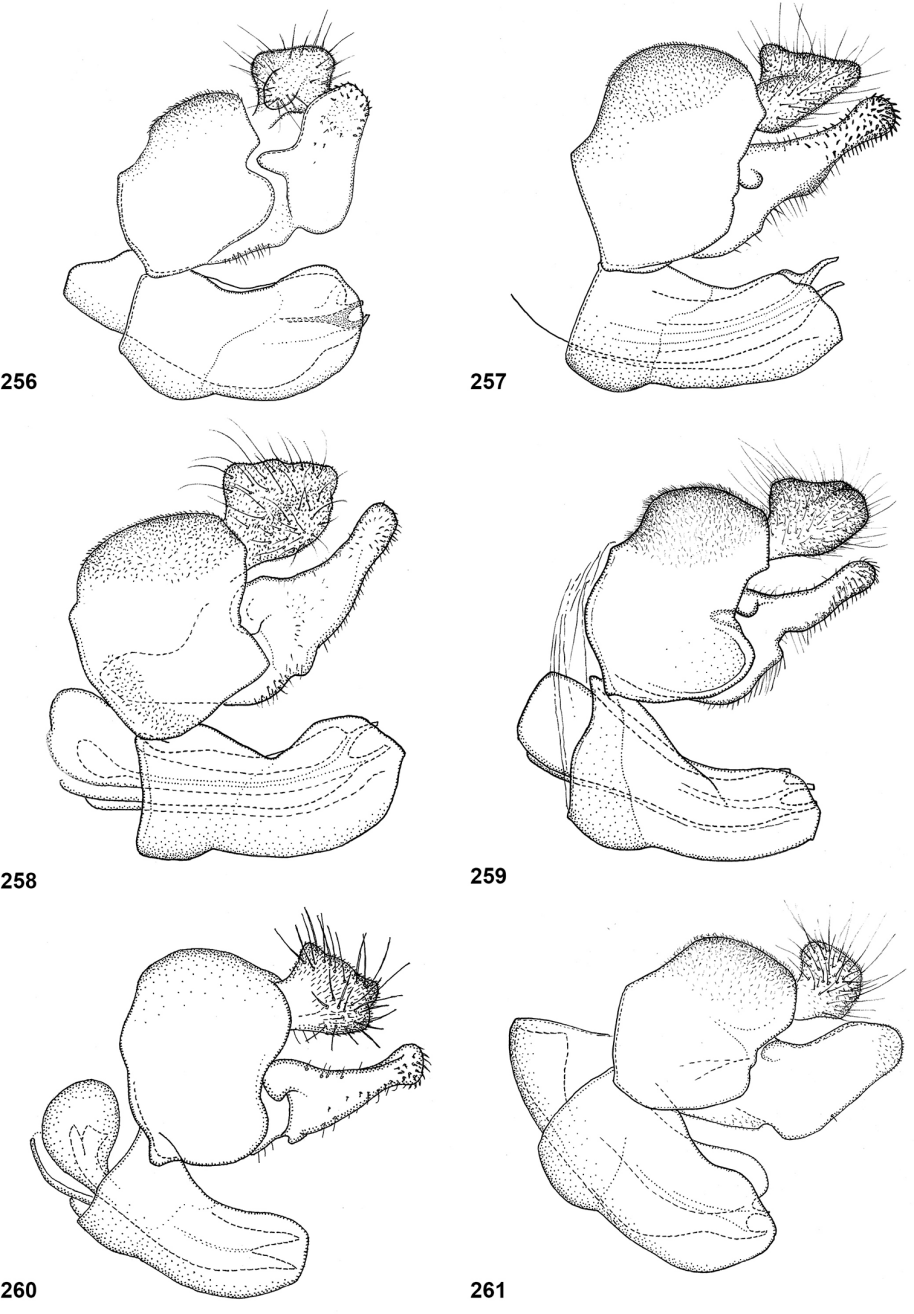
Male genitalia of *Peradon* species **256***P.fenestratus* French Guyana MNHN**257***P.aureoscutus* holotype **258***P.aureus* Ecuador CNC**259***P.trilinea* holotype **260***P.trivittatus* Suriname RMNH**261***P.hermetia* holotype.

**Figures 262–267.**
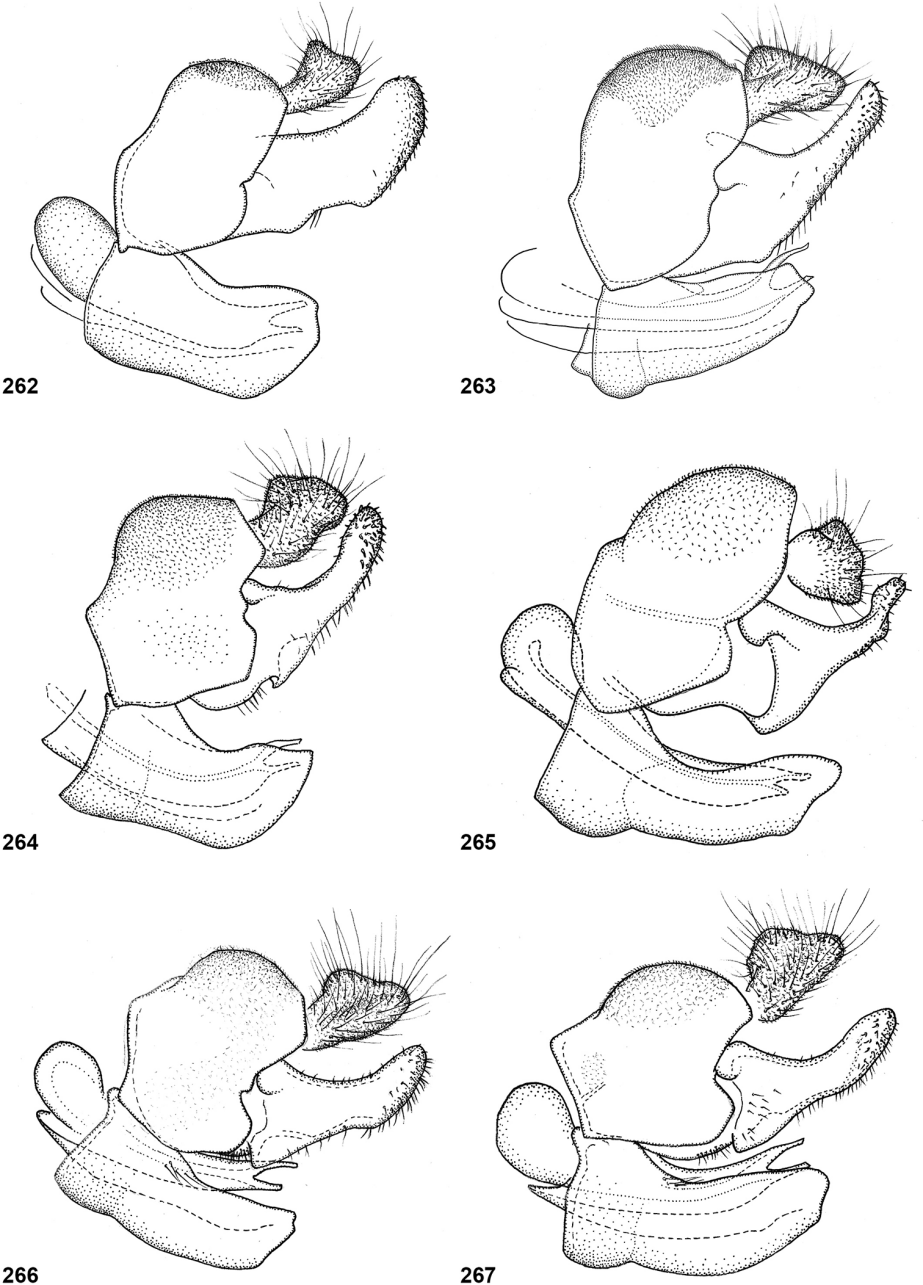
Male genitalia of *Peradon* species **262***P.hermetoides* holotype **263***P.elongatus* holotype **264***P.oligonax* Colombia NHMUK**265***P.satyricus* holotype **266***P.sciarus* paratype **267***P.bidens* Suriname RMNH.

**Figures 268–273.**
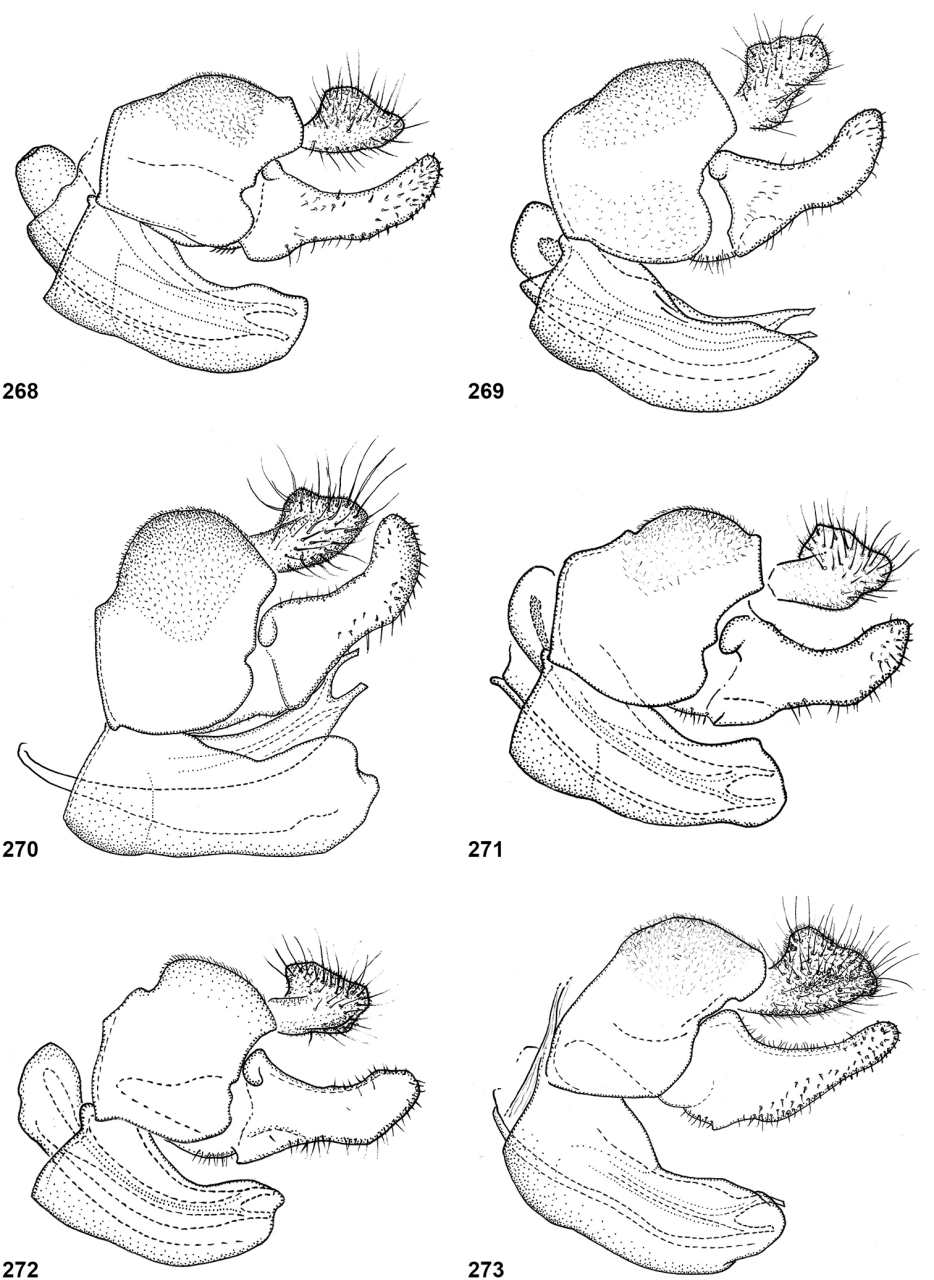
Male genitalia of *Peradon* species **268***P.costaricensis* paratype **269**P.?normalis Brazil Rondonia LACM**270***P.niger* holotype **271***P.bispina* holotype **272***P.pompiloides* holotype **273***P.aurifascia* Sao Paulo CNC.

**Figures 274–279.**
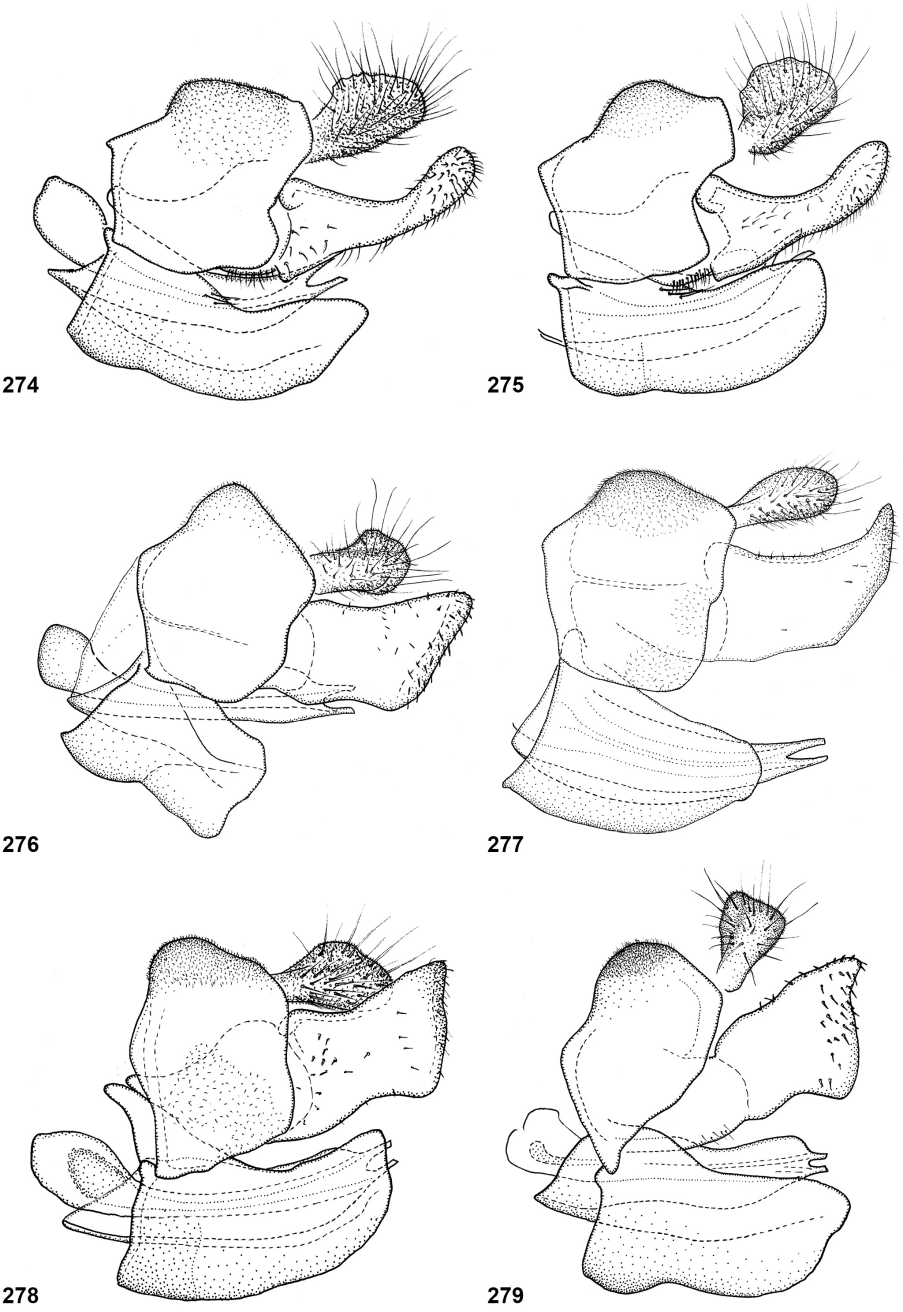
Male genitalia of *Peradon* species **274***P.luridescens* Suriname RMNH**275***P.angustiventris* Suriname RMNH**276***P.palpator* holotype **277***P.flavofascium* holotype **278***P.surinamensis* holotype **279***P.notialus* holotype.

**Figures 280–283.**
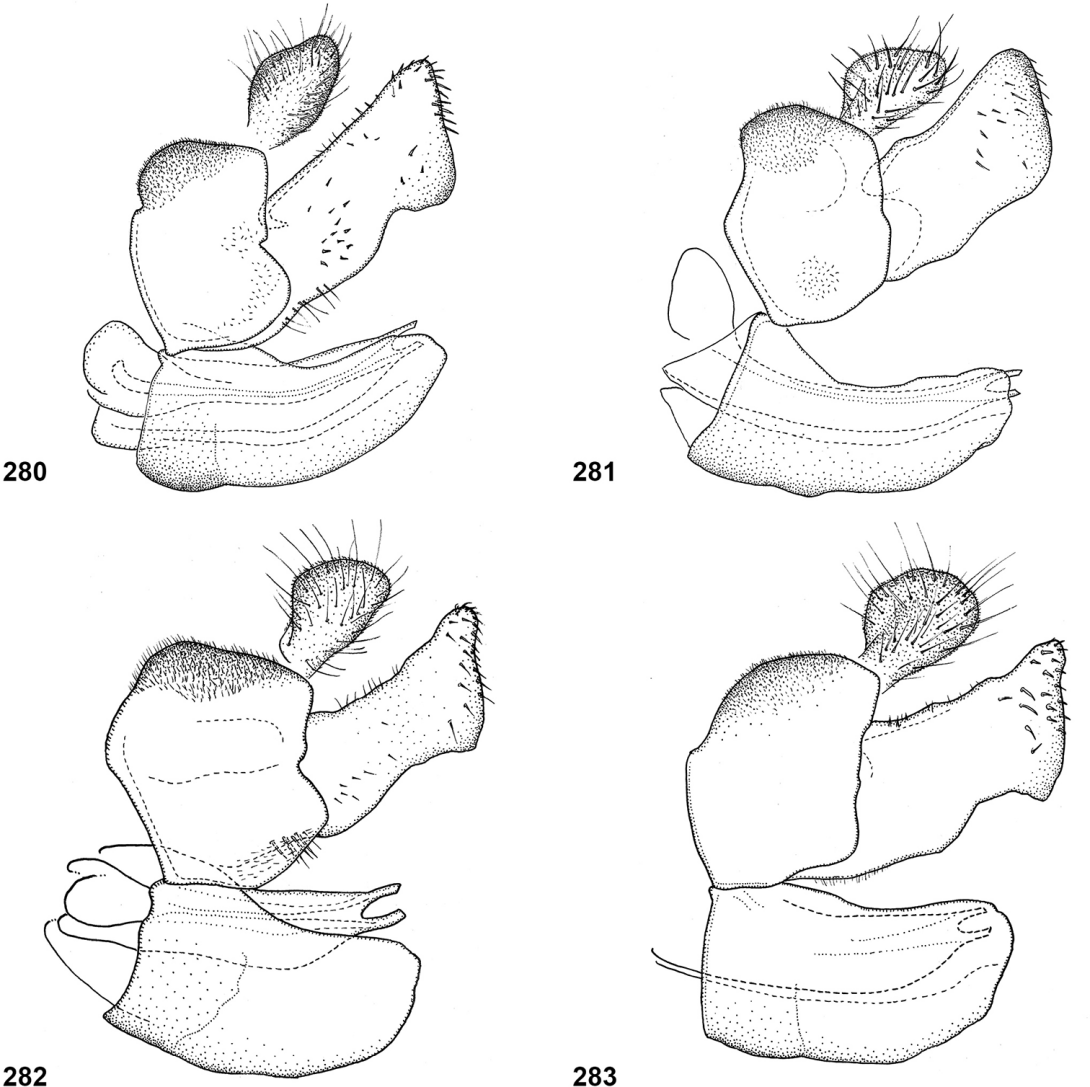
Male genitalia of *Peradon* species **280***P.brevis* holotype **281***P.chrysopygus* Costa Rica RMNH**282***P.ballux* holotype **283***P.aurigaster* Bolivia RMNH.

## Discussion

With the description of seven new species and the establishment of two new synonyms, the number of valid species of *Peradon* now reaches 31. Colour variation is considerable in several species of *Peradon*. Especially in cases where available specimens are few, the taxonomic significance of such variation is hard to assess. In some taxa, discrete colour morphs are recognized, most notably in *P.bidens* and *P.normalis*. Discrete colour polymorphism has been described in a few other species of Microdontinae: *Microdoncothurnatus* Bigot, 1883 (Akre et al. 1973), *Microdonlanceolatus* Adams, 1903 (Thompson 1981) and *Microdonmyrmicae* Schönrogge et al., 2002 (Wolton 2017). In these cases, the polymorphism is restricted to the colour of the pilosity. The cases presented in the present paper are the first ones known in Microdontinae which concern the colour of the integument. The ecological significance of this colour variation is unknown, but possibly it is associated with the selective advantages of mimicry.

Reemer and Ståhls (2013a, b) distinguished three morphological species groups within *Peradon*, whilst recognizing that these groups may not be monophyletic. The main structure of the identification key to the *Peradon* species in the present paper is based on these three species groups as they were still found to be useful for this purpose, although the present work did not try to resolve the monophyly of these species groups.

Within the *bidens* group, the intraspecific divergence of COI barcodes is greater than the interspecific divergence. So, these barcode sequences are not suitable for distinguishing between species within the species group. Among the three included species of the *flavofascium* group divergences are much larger. Whether this is also the case for the other species of the *flavofascium* group or for the *trivittatus* group is hard to say, because only few species were sampled from these groups (onyl one from the *trivittatus* group).

There are several other known instances in Syrphidae in which barcodes of morphologically distinct species are highly convergent and cannot be used for species delimitation. One example is found in *Melanostoma* Schiner, 1860, in which multiple haplotypes are shared between species (Haarto and Ståhls 2014). Other examples are known from the genus *Merodon* Meigen, 1803 (e.g., Milankov et al. 2008, Popović et al. 2015). Sometimes, as in *Melanostoma*, additional molecular markers may help in solving the species taxonomy. Whether this will also be the case in the *bidens* group of *Peradon* would be interesting to find out.

## Supplementary Material

XML Treatment for Peradon angustiventris

XML Treatment for Peradon angustus

XML Treatment for Peradon aureoscutus

XML Treatment for Peradon aureus

XML Treatment for Peradon aurifascia

XML Treatment for Peradon aurigaster

XML Treatment for Peradon ballux

XML Treatment for Peradon bidens

XML Treatment for Peradon bispina

XML Treatment for Peradon brevis

XML Treatment for Peradon chrysopygus

XML Treatment for Peradon costaricensis

XML Treatment for Peradon diaphanus

XML Treatment for Peradon elongatus

XML Treatment for Peradon fenestratus

XML Treatment for Peradon flavipennis

XML Treatment for Peradon flavofascium

XML Treatment for Peradon hermetia

XML Treatment for Peradon hermetoides

XML Treatment for Peradon luridescens

XML Treatment for Peradon niger

XML Treatment for Peradon normalis

XML Treatment for Peradon notialus

XML Treatment for Peradon oligonax

XML Treatment for Peradon palpator

XML Treatment for Peradon pompiloides

XML Treatment for Peradon satyricus

XML Treatment for Peradon sciarus

XML Treatment for Peradon surinamensis

XML Treatment for Peradon trilinea

XML Treatment for Peradon trivittatus

## Footnotes

No females of Peradon ballux are known, so its placement in this key is based on characters of the male and should be considered tentative.

## Supplementary materials

Supplementary material 1 Figure S1. Strict consensus of the ten most parsimonious trees based on COI barcodes of *Peradon* specimens Data type: phylogenetic data Explanation note: Values above branches indicate branch lengths, values below branches (in italic) are bootstrap support values (left) and GC frequency differences (right). https://binary.pensoft.net/file/362319This dataset is made available under the Open Database License (http://opendatacommons.org/licenses/odbl/1.0/). The Open Database License (ODbL) is a license agreement intended to allow users to freely share, modify, and use this Dataset while maintaining this same freedom for others, provided that the original source and author(s) are credited.Menno Reemer, Jeffrey H. Skevington, Scott Kelso

Supplementary material 2 Figure S2. Neighbour Joining tree based on the COI barcodes of *Peradon* specimens Data type: phylogenetic data https://binary.pensoft.net/file/362320This dataset is made available under the Open Database License (http://opendatacommons.org/licenses/odbl/1.0/). The Open Database License (ODbL) is a license agreement intended to allow users to freely share, modify, and use this Dataset while maintaining this same freedom for others, provided that the original source and author(s) are credited.Menno Reemer, Jeffrey H. Skevington, Scott Kelso
