# Supplementary figures and images for: Revision of the Neotropical hoverfly genus Peradon Reemer (Diptera, Syrphidae, Microdontinae)
Source: Zookeys. 2019 Dec 5;896:1–93. doi: 10.3897/zookeys.896.36493 (PMC6908515; doi:10.3897/zookeys.896.36493)

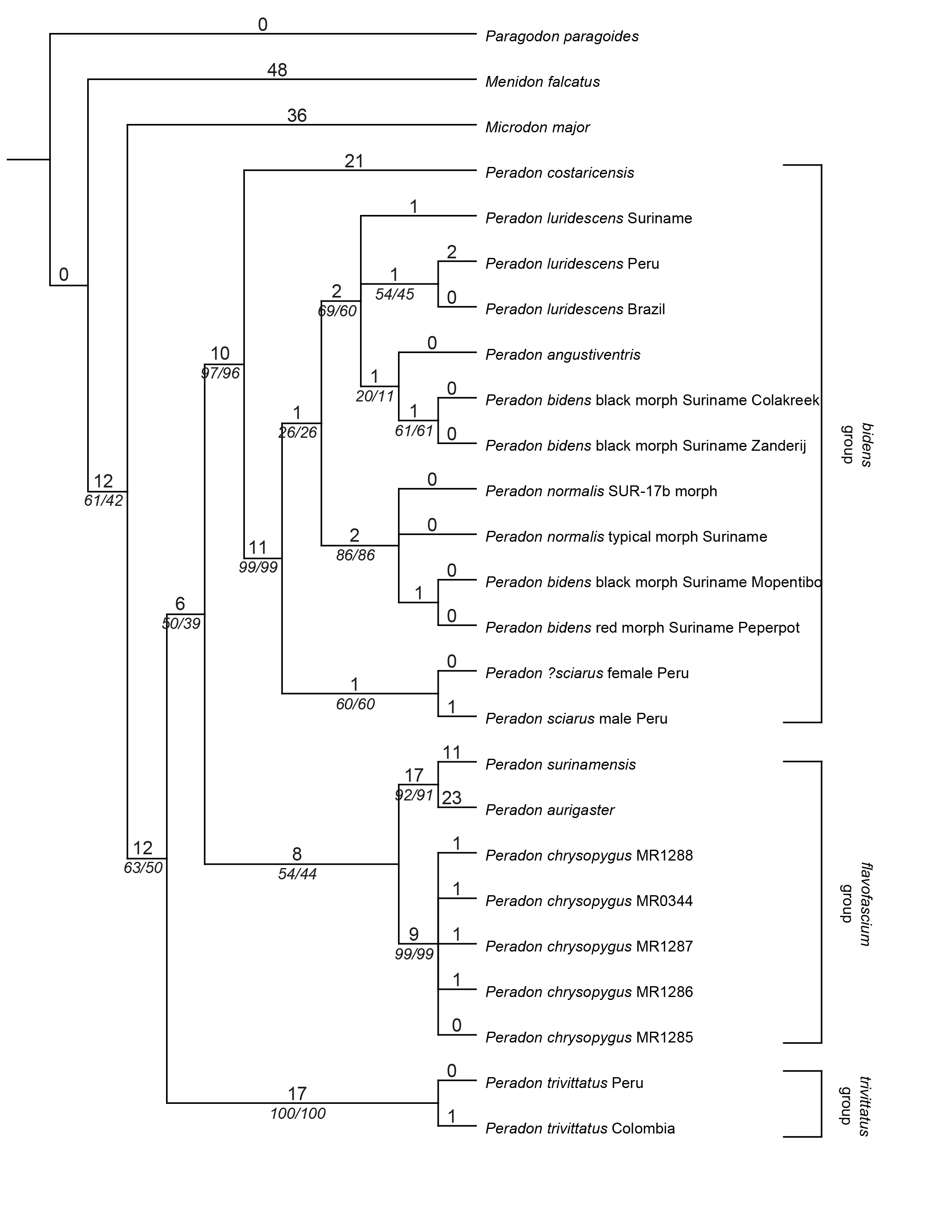

Supplement: Supplementary material 1 [file zookeys-896-001-s001.jpg]

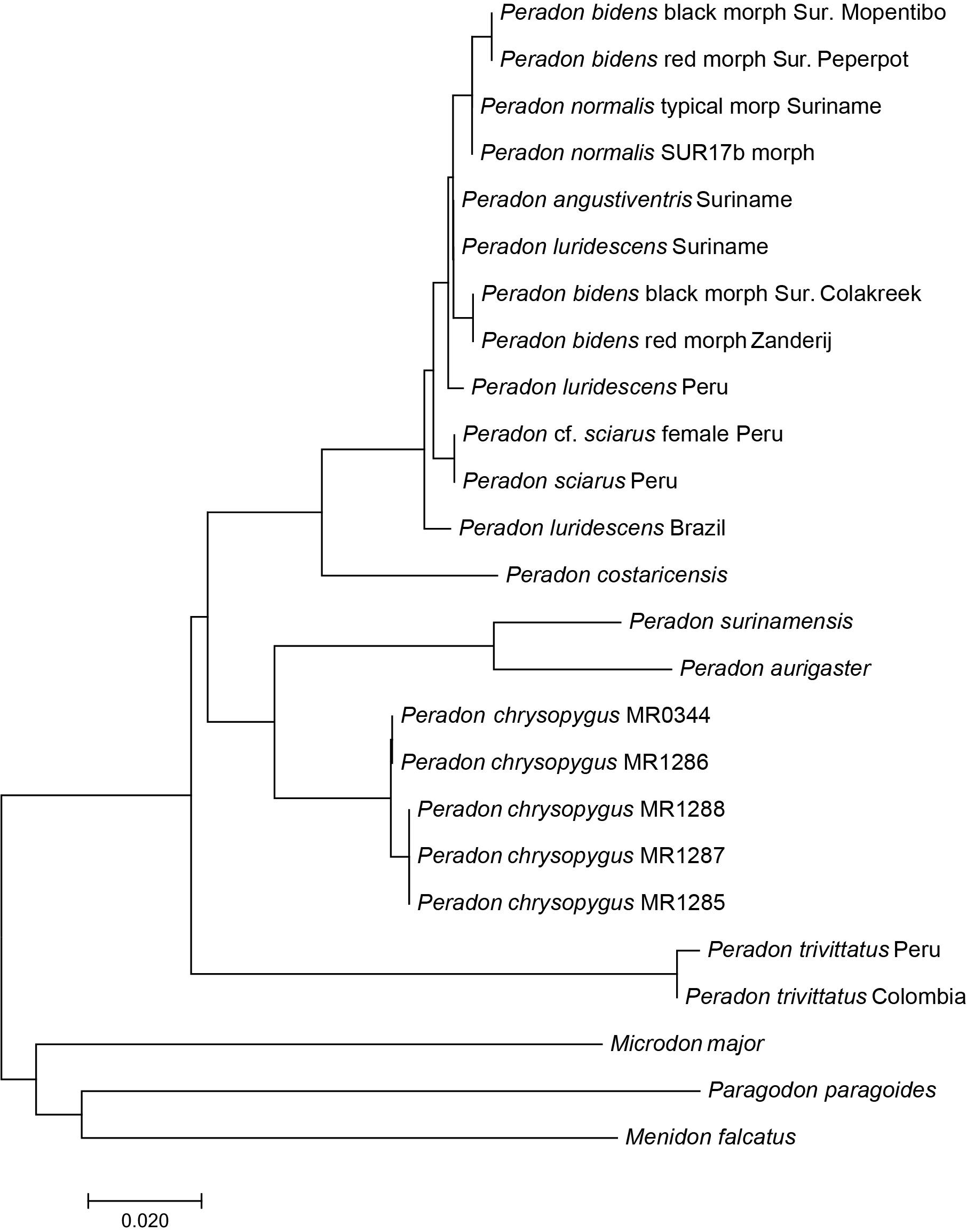

Supplement: Supplementary material 2 [file zookeys-896-001-s002.jpg]
